# *In Silico* Analysis of Gene Expression Network Components Underlying Pigmentation Phenotypes in the Python Identified Evolutionarily Conserved Clusters of Transcription Factor Binding Sites

**DOI:** 10.1155/2016/1286510

**Published:** 2016-09-06

**Authors:** Kristopher J. L. Irizarry, Randall L. Bryden

**Affiliations:** ^1^The Applied Genomics Center, Graduate College of Biomedical Sciences, Western University of Health Sciences, 309 East Second Street, Pomona, CA 91766, USA; ^2^College of Veterinary Medicine, Western University of Health Sciences, 309 East Second Street, Pomona, CA 91766, USA

## Abstract

Color variation provides the opportunity to investigate the genetic basis of evolution and selection. Reptiles are less studied than mammals. Comparative genomics approaches allow for knowledge gained in one species to be leveraged for use in another species. We describe a comparative vertebrate analysis of conserved regulatory modules in pythons aimed at assessing bioinformatics evidence that transcription factors important in mammalian pigmentation phenotypes may also be important in python pigmentation phenotypes. We identified 23 python orthologs of mammalian genes associated with variation in coat color phenotypes for which we assessed the extent of pairwise protein sequence identity between pythons and mouse, dog, horse, cow, chicken, anole lizard, and garter snake. We next identified a set of melanocyte/pigment associated transcription factors (CREB, FOXD3, LEF-1, MITF, POU3F2, and USF-1) that exhibit relatively conserved sequence similarity within their DNA binding regions across species based on orthologous alignments across multiple species. Finally, we identified 27 evolutionarily conserved clusters of transcription factor binding sites within ~200-nucleotide intervals of the 1500-nucleotide upstream regions of AIM1, DCT, MC1R, MITF, MLANA, OA1, PMEL, RAB27A, and TYR from* Python bivittatus*. Our results provide insight into pigment phenotypes in pythons.

## 1. Introduction

Color and pattern variation within, and across, species provide a unique opportunity to investigate the genetic basis of evolution and selection. Pigmentation variation in snakes and lizards serves a variety of ecological roles. Pigmentation patterns on dorsal surfaces can provide camouflage from visual predators, such as birds. Pigmentation can also provide warnings to potential predators regarding specific toxins or venoms. Additionally, sexually dimorphic pigmentation may facilitate communication among conspecifics relating to reproduction and territorial control [1].

To date, the mouse has been the most studied organism in the dissection of genes modulating coat color and pattern. Although more than 100 genes have been identified which contribute to pigmentation and color phenotypes, the emerging view is that although some genes specifically control color while other genes affect patterns, the tremendous diversity in color and patterns arises through the epistatic interaction between color and pattern genes [2].

Unlike many phenotypes, in which mammals exhibit greater complexity than amphibians, reptiles, and fishes, mammalian color determination is simpler because mammals exhibit much less color variation than other classes of vertebrates. In contrast to mammals, in which color is determined by a single cell type (melanocyte), color in reptiles, amphibians, and fishes arises from three cell layers: the melanophore, the xanthophore, and the iridophore [3].

Color and pattern phenotypes are valuable provided they can be perceived. Eye development in squamates involves formation of the retinal pigmented epithelium (RPE), which is required for normal physiological function of the eye [4]. In mammals, the RPE is contiguous with the iris pigment epithelium (IPE), both of which contain the pigment melanin [5]. Most snakes have three distinct photoreceptors exhibiting wavelength absorption peaks of 357–365 nm (UV), 482–495 nm (blue), and LWS 535–560 nm (green). Pythons in particular, such as the ball python (*Python regius*), have rods with peak absorption at 495 nm representing 90% of their photoreceptors while the remaining 10% of photoreceptors are comprised of two types of single cones absorbing at 360 nm and 550 nm [6].

**Figure 1:**
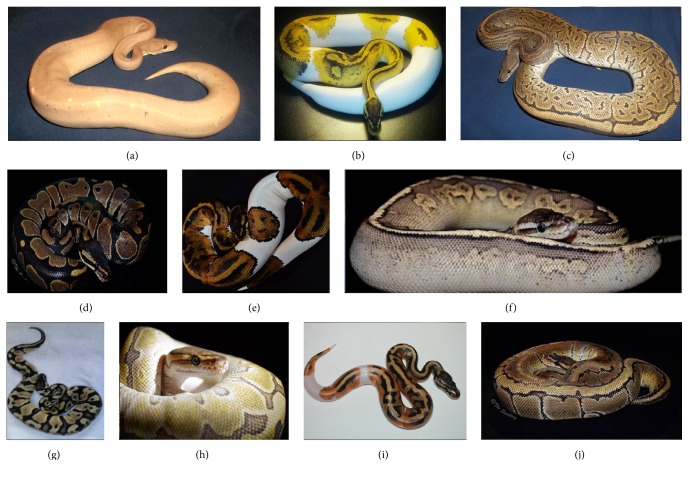
Color and pattern phenotypes in ball pythons (Part 1). Observed variation in pigmentation and color morphological phenotypes provides evidence for genetic modulation of these phenotypes. (a) Homozygous cinnamon (codominant), heterozygous banana (codominant). (b) Homozygous piebald (recessive), heterozygous pastel (codominant). (c) Heterozygous black pastel (codominant), heterozygous pinstripe (dominant). (d) Wild type. (e) Piebald (recessive). (f) Heterozygous cinnamon (codominant), heterozygous pastel (codominant). (g) Heterozygous pastel (codominant). (h) Heterozygous Lesser (codominant), heterozygous pinstripe (dominant). (i) Homozygous piebald (recessive). (j) Pinstripe (dominant).

As of 2016, ball python breeders have produced over 4600 distinct genetic strains of color and pattern morph variants (http://www.worldofballpythons.com/morphs/). Many of these color and pattern phenotypes exhibit Mendelian modes of inheritance such as simple dominant, incomplete dominant, codominant, and recessive modes. Moreover, the breeding data from some color and pattern morphs suggests sex linked modes of inheritance are also represented [7]. Altogether, the phenotypic variation of ball pythons represents a valuable resource to explore the genetic basis of morphological variation in snakes and other reptiles. Some examples of ball python morphs are depicted in Figure 1.

Color phenotypes in vertebrates arise through specific type of pigments deposited within the ectodermal appendage (hair, scales, or feathers) and the spatial distribution of those pigment types throughout the organism's body. Genetic factors influencing the types of pigments produced include two genes of main effect, the melanocortin receptor (*Mc1R*) and* Agouti*, the receptor's antagonist.* Mc1R* normally produces dark brown to black pigment called eumelanin, while binding of* Agouti* results in production of the yellow-red pigment pheomelanin [8].

A recent study investigating the mechanistic basis for the extensive color variation observed in* Phelsuma* lizards described two distinct melanophores which give rise to lateral spots and stripes: one, called a chromatophore unit, composed of light dark brown dendritic cells associated with iridophores and the other, a larger darker brown cell located deep in the dermis. The chromatophore unit is able to translocate melanin granules in response to hormones which results in darker skin coloration. Additionally, red erythrophore pigments and yellow xanthophore pigments from the pteridine class (which are similar xanthopterin found in squamates), as well as biopterin (found in both the yellow and red chromatophores), contribute to the red and green colors of the skin [9]. Additionally, the lizard's skin contains iridophores, with guanine nanocrystals, that exist either (a) as well-organized parallel layers or (b) in a highly disorganized state. These nanocrystals are capable of producing blue or green color via a multilayer interference mechanism when they are in the ordered state while the disorganized pattern causes a red skin color resulting from scattering of light.

Indeed, the cellular, molecular, and biochemical mechanisms contributing to color and pattern variation in reptiles are complex and may utilize processes that are distinct from those found in mammals. Nevertheless, computational approaches aimed at analyzing the python genes known to modulate pigmentation in other species, such as horses, dogs, and mice, and people can provide additional insight into those mechanisms that might be present in mammals and conserved across vertebrates.

In mammals, melanocytes synthesize the pigment melanin (eumelanin and pheomelanin) via the action of three main enzymes: tyrosinase (TYR), TYR-related protein (TRP-1), and dopachrome tautomerase (DCT). Once produced, melanin accumulates in melanosomes which ultimately are transferred to keratinocytes. Numerous factors control melanocyte differentiation and proliferation, including coat color genes (*Agouti*,* Tryp-1*,* Myo5a*,* Mc1R*,* Oca2*,* DCT*, and* Hps5*), the keratinocyte derived factors* endothelins 1, 2, and 3* (Edn1, Edn2, and Edn3), leukemia inhibitory factor (LIF), and kit ligand (kitlg), as well as ultraviolet light [10]. Microphthalmia-associated transcription factor (MITF) is a key regulator of the gene expression network modulating melanocyte development, survival, and proliferation, as well as melanin production. MITF transactivates a number of melanocyte gene products including* Mc1R, EDNRB*,* Rab27A*,* OA1*,* PMEL*,* MLANA*, and* AIM1* [11].

Cells, such as melanocytes, derived from the neural crest are short-lived progenitors that migrate outwards along predefined trajectories to a variety of locations in the developing embryo. These neural crest cells differentiate into numerous cell types including neurons, glia, melanocytes, iridophores, xanthophores, and erythrophores and as well as cells associated with connective tissue such as chondrocytes, odontoblasts, osteoblasts, and osteoclasts. The neural crest is regulated by Bmp and Wnt families of growth factors which are expressed in nonneural ectoderm during gastrulation and are responsible for induction of the neural crest [12]. A complex repertoire of transcription factors provide genetic control of neural crest development, with Pax, Msx, and Dlx members modulating neural folds and neural plate formation, while Twist, Snail, Sox9, Sox10, and FoxD3 are expressed in premigratory neural crest during segmentation in which they regulate neural crest specification and neural crest cell delamination [13]. The transcription factors Pax3, Pax7, and Sox10 are required for melanocyte differentiation whereby they regulate expression of MITF, which further facilitates melanocyte differentiation through the transcriptional activation of DCT, TYR, and PMEL [14].

The genes underlying melanocyte biology and pigment formation are associated with numerous phenotypes and disorders affecting sensory organs and nerves, fitness and reproduction, behavior, melanoma, immune function, and metabolism [15]. Skin pigmentation arises through the activity of melanin localized to membrane bound organelles that are transferred from melanocytes to keratinocytes. Melanosomes are transported outward from the center of the melanocyte to its periphery via motor proteins and microtubules. RAB27A is involved in the attachment of melanosomes to myosin and may also participate in the docking of melanosomes at the plasma membrane of melanocytes along with SNARE proteins. Interestingly, genetic defects associated with disruptions of lysosomal transport and secretion can give rise to immunological, coagulation, and pigmentation phenotypes [16]. The transfer of melanin, from the melanocyte to keratinocytes, occurs through filopodia extensions that create a cellular bridge between cells [17].

Although relatively few genomic resources are available for investigating genetics in the ball python, the Burmese python (*Python molurus bivittatus*) has genomic sequence publicly available along with 20,392 genes encoding 25,943 protein sequences [18]. Because the genomic scaffold sequences are available in the NCBI nucleotide repository, it is possible to obtain upstream sequences for the genes which represent transcriptional regulatory regions.

The Burmese python is a semiaquatic, invasive, apex predator in the Florida Everglades. Its unique cryptic coloration pattern provides camouflage which makes it difficult to detect. Subsequently, techniques for environmental sampling of DNA from water sources in the Everglades provide a means of assessing the presence and numbers of Burmese pythons in the absence of direct visual observation [19]. Our choice to investigate pigmentation genetics in the Burmese python was based on its evolutionary and phylogenetic relationship with the ball python, which makes it an ideal genetic model for the ball python. Because the much smaller and more docile ball python has been selectively bred specifically for color and pattern phenotypes; the genomic resources available for the Burmese python provide a genomic framework for investigating the pigmentation biology in pythons.

Most importantly, the identification and characterization of genes underlying color and pattern phenotypes in the Burmese python may provide tools for tracking the subsequent evolution of this invasive species. Specifically, genetic variation within genes associated with pigmentation and coloration may be useful in molecularly assessing environmental DNA samples in order to quantify phenotypic changes that may contribute to increased fitness of this species over time.

In an attempt to assess the extent of the shared molecular biology underlying pigmentation physiology between the Burmese python and mammals, we utilized the following hypothesis.

*Shared pigmentation biology between species can be identified through the detection of genomic fingerprints associated with conserved transcriptional regulatory networks underlying melanocyte biology and melanin formation*.

Our rationale for this approach is that the successful identification of conserved transcriptional regulatory motifs between python promoters and mammalian promoters would provide evidence of common regulation of genes involved in pigmentation and coloration phenotypes. Specifically, the application of bioinformatics to infer conserved cellular genetic programs in melanocytes based on comparative genomics, supervised literature mining, and regulatory motif analysis provides an initial step to identify conserved regulatory modules between vertebrates that are involved in pigmentation.

## 2. Materials and Methods

### 2.1. Identification of* Python bivittatus* Orthologs of Mammalian Coat Color Genes

The set of mammalian coat color genes were identified from Table 1 in the publication “Pleiotropic Effects of Coat Color-Associated Mutations in Humans, Mice, and Other Mammals” [15]. The choice to use this set of genes was based, in part, on the fact that this gene set included phenotypic/disease information for each of the pleiotropic genes included in the table. Additional consideration was given for the relatively small number of genes in the table for which each gene has a specific pigmentation phenotype, specific allele, and disease/undesirable phenotype association.

A total of 24 genes were listed in the table. A set of 23 genes were identified for which python protein sequences were available in NCBI protein repository (see Table 1). Each gene symbol listed in the table was used to search the NCBI protein database along with the search string* Python bivittatus*. For each list of resulting proteins, the potential orthologs were manually searched for a single protein with an identical name as listed in the table (which may have included the term “-like” after the protein name).

### 2.2. Human, Mouse, Dog, Horse, Cow, Chicken, Lizard, and Garter Snake Orthologs of Coat Color Genes

Protein orthologs for each python coat color gene were identified using the NCBI protein BLASTP server (http://blast.ncbi.nlm.nih.gov/Blast.cgi?PAGE=Proteins) [20]. For each FASTA formatted python protein query sequence, BLASTP was used to search NCBI for all nonredundant GenBank CDS translations + PDB + SwissProt + PIR + PRF excluding environmental samples. Additional constraints placed on the search included limiting the results to the following specific taxonomy identifiers: 9606, 10088, 9615, 9796, 9913, 9031, 28376, and 35019 (corresponding to the following species:* Homo sapiens, Canis lupus familiaris, Mus musculus, Equus caballus, Bos taurus, Gallus gallus, Anolis carolinensis,* and* Thamnophis sirtalis*). In some cases when the an ortholog was not identified for horse or cow, a subsequent search using a related species in the same genus occurred, for example,* Equus przewalskii* in place of* Equus caballus*.

The BLAST results reported by NCBI were manually reviewed to identify the true ortholog and exclude any paralogs. When a specific species lacked the true ortholog, a false positive result corresponding to a paralog was typically provided by BLAST. Visual inspection of results helped exclude paralogs and other undesired proteins during the ortholog detection process. The percent identity for each species specific ortholog pair was obtained from the results. The reported percent identity for each query-subject species pair corresponds to the best blast hit produced during the BLAST search. In some cases, the percent identity was calculated for an alignment length less than the actual length of the protein sequence.

### 2.3. Construction of Orthologous Coat Color Protein Identity Heat Map

The multispecies, pairwise, ortholog, percent identity data was formatted as displayed in Table 1 and saved as a tab delimited text file. The gene names were modified to include the pleiotropic effects contained in parentheses. The file was uploaded to the Morpheus heat map building tool (http://www.broadinstitute.org/cancer/software/morpheus/) available from the Broad Institute. The file was formatted to conform with the expected column headings as described in the input file documentation. Custom colors were selected to represent high sequence identity (red), moderate sequence identity (magenta), and low sequence identity (blue). The heat map is displayed in Figure 3.

### 2.4. Construction of Python Pigmentation Gene Expression Network Components

The pigmentation gene expression network components were identified by reviewing the relevant literature covering transcriptional regulation of melanocyte differentiation and melanin production. In total, thirteen papers spanning 20 years (from 1994 to 2014) provided detailed information about the specific transcription factors, promoters, regulatory elements, transcription factor binding sites, and target genes (Table 2).

After reviewing the relevant information contained in the references, the following pigmentation gene expression network components were selected for inclusion in subsequent analysis:

1. Transcription factors: CREB, FOXD3, LEF-1, MITF, POU3F2, and USF-1.
2. Target gene promoters: AIM1, DCT, MC1R, MITF, MLANA, OA1, PMEL, RAB27A, and TYR.
3. Upstream regions: 1500 bases upstream of the transcription start site.

### 2.5. Protein Alignments of Python Pigmentation Associated Transcription Factors

Alignments for each python pigmentation associated transcription factor were generated in order to assess the extent of amino acid similarity across a variety of vertebrates. Multiple sequence alignments were generated using CLC Sequence Viewer 6 (http://www.clcbio.com/products/clc-sequence-viewer/). Phylogenetic trees were produced using the tree constriction algorithm in CLC Viewer 6. Alignments were colored to reflect regions of conservation (blue) and regions of divergence (red); some alignments depict moderate sequence similarity (magenta).

### 2.6. Protein Domain Analysis of Python Pigmentation Associated Transcription Factors

The python transcription factor proteins were analyzed to identify the start and end positions of functional domains within each protein sequence. The online InterPro 54.0 server was used to identify protein domains. FASTA formatted protein sequences were submitted to the InterPro domain server (http://www.ebi.ac.uk/interpro/) [21].  Table 3 contains domain annotations for these proteins.

### 2.7. Identification of Transcription Factor Binding Sites in Pigmentation Gene Upstream Regions

Transcription factor binding sites (TFBS) were identified in the 1500 nucleotide upstream sequences from the target genes (AIM1, DCT, MC1R, MITF, MLANA, OA1, PMEL, RAB27A, and TYR) using the PROMO virtual laboratory (http://alggen.lsi.upc.es/cgi-bin/promo_v3/promo/promoinit.cgi?dirDB=TF_8.3) [22, 23]. The transcription factor binding site data was based on TRANSFAC version 8.3 (http://www.biobase-international.com/product/transcription-factor-binding-sites) [24].

Transcription factor binding sites were identified in each 1500-nucleotide upstream sequence separately and also within the group of all nine upstream regions. When the upstream regions were analyzed as a group, the following parameter settings were used: (1) sites found in one or more sequences and (2) maximum matrix dissimilarity rate = 15%. The decision to use 15% dissimilarity rate was based on the fact that the python regulatory regions and transcription factor DNA binding regions may diverge from the mammalian counterparts. Subsequently, stringent matrix (dissimilarity ≪ 15%) may result in false negatives. It is also true that the higher dissimilarity rate may result in false positives within the data set. Since these are computational predictions of protein-nucleic acid binding sites and potential macromolecular complexes, the predicted molecular binding results should be treated as candidate binding sites until being experimentally verified.

### 2.8. Detection of Evolutionarily Conserved Clusters of Transcription Factor Binding Sites

Conserved clusters of transcription factor binding sites located within the pigmentation associated target genes were identified using sliding windows of variable size to identify intervals within the upstream regions that were enriched for two or more distinct transcription factor binding sites. Although TRANSFAC represents each instance of a particular transcription factor's binding site as a unique entry in the binding site database, we collapsed the numerous versions of each binding site into a single class of binding site that was associated with each unique transcription factor. The criteria requiring at least two distinct transcription factors provide a mechanism for identifying regulatory regions that potentially function combinatorically to modulate transcription synergistically. In order to maximize the significance of the binding site clusters, we chose to identify relatively small intervals, of approximately 200 nucleotides for which the greatest number of pigmentation transcription factor binding sites occurred. We employed an algorithm in which the interval containing the greatest number of binding sites was identified first, and then the process was repeated two more times, for each upstream region. Correspondingly, we identified three ~200-nucleotide intervals within each upstream region.

### 2.9. Visualization of Candidate Enhancer Modules in Python Pigmentation Gene Promoter Sequences

To better visualize the candidate pigmentation enhancer modules within each upstream region, we developed a simple schema for representing the evolutionarily conserved transcription factor binding sites within each identified promoter interval. First, each transcription factor binding site was color coded with a single color mapping to each distinct transcription factor such that* CREB* binding sites are green,* FOXD3* binding sites are magenta, *LEF-1* binding sites are orange,* MITF* binding sites are blue,* POU3F2* binding sites are red, and* USF-1* binding sites are purple.

Next, we chose to visualize the candidate enhancer modules such that the following information would be quickly and easily extracted from each interval's visual representation: (1) the type and frequency of binding sites within the interval; (2) the* total number* of binding sites within the interval; (3) the* linear arrangement* of binding sites within the interval; (4) the* specific transcription factor* that binds to each site; (5)* binding sites that overlap* with binding sites for a different transcription factor; (6) the* relative location of the candidate enhancer* modules within each upstream region; (7) the* relative density of binding sites* within each candidate enhancer module; and (8) the* relative nucleotide length* for each candidate enhancer module.

In order to develop visualization schema that accomplished those eight goals, we recognized that other types of information would not be easily extracted from the visualization. We willingly accepted these trade-offs in information content related to the visualization. Specifically, the following types of information are not easily inferred from our enhancer module visualization method: (a) the orientation of the transcription, (b) the strand that the transcription factor binds to, (c) the relative spacing between the binding sites within each interval, (d) the exact distribution of binding sites within the candidate enhancer module, (e) the length in nucleotides of each binding site, (f) the linear nucleotide distance between specific binding sites within the candidate module, and (g) the nucleotide composition of the binding sites and/or module.

### 2.10. Public Release of Study Data Sets

The nucleotide and amino acid sequences described in this study are publicly available in the form of supplemental files associated with this paper (in Supplementary Material available online at http://dx.doi.org/10.1155/2016/1286510). Additionally, all of the raw transcription factor binding site data is also freely available to the research community. The table describing the gene expression network components (along with the associated information extracted from each reference) is included in this paper in order to facilitate public access to resources needed to further investigate the role of these candidate regulatory enhancers and their associated transcription factor binding sites.

Supplemental File 1 contains the transcription factor binding site data produced by PROMO.

Supplemental File 2 contains the FASTA formatted nucleotide sequence corresponding to the 1500-base pair upstream region for each gene analyzed for transcription factor binding sites.

Supplemental File 3 contains the FASTA formatted protein sequences (including orthologs from other species) for the transcription factors described in this study.

## 3. Results

### 3.1. Pigmentation Phenotypes in Pythons

**Figure 2:**
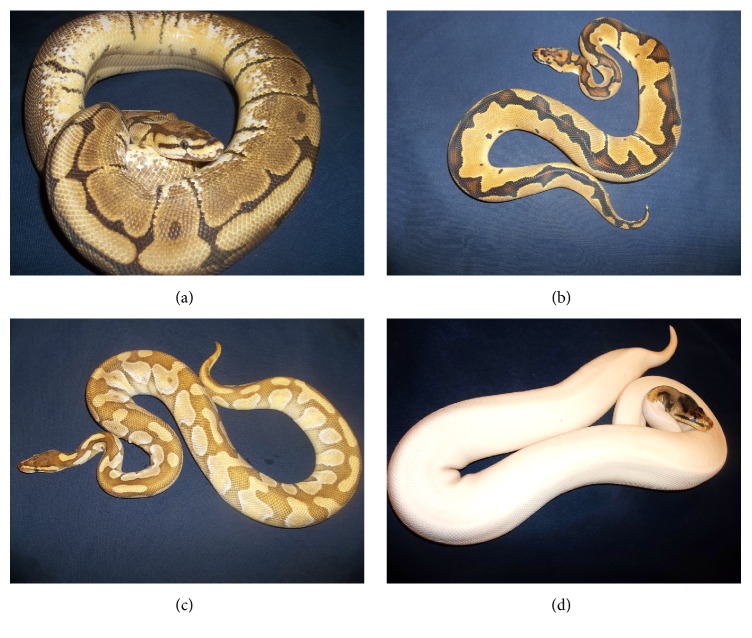
Color and pattern phenotypes in ball pythons (Part 2). Observed variation in pigmentation and color morphological phenotypes provides evidence for genetic modulation of these phenotypes. (a) Heterozygous spider (dominant). (b) Homozygous Clown (recessive). (c) Heterozygous Mojave (codominant), heterozygous Enchi (codominant). (d) Homozygous piebald (recessive), heterozygous spider (dominant). The examples of ball python (*Python regius*) morphological phenotypes are used to illustrate phenotypic diversity in pythons and are not meant to suggest any direct causative relationship between the color and pigmentation phenotypes illustrated and the resulting analysis of* Burmese python* genomic sequence.

Ball pythons exhibit a large number of color and pattern phenotypes (Figures 1 and 2). The wild type ball python (Figure 1(d)) is dark brown with black and yellow highlights. Some phenotypes affect only the coloration, while other phenotypes do not alter color but instead modify the pattern. Some phenotypes affect both color and pattern. For example, the pastel phenotype (Figure 1(g)) is a codominant dilution phenotype which lightens the color without altering the pattern. Homozygous pastel phenotype (not shown) is considerably lighter than the heterozygous phenotype.

The piebald phenotype modifies both the pattern and the color (Figures 1(e) and 1(i)). Note that the lighter coloration observed in the homozygous piebald phenotype is accompanied by a dramatically altered pattern in which the normal patterning observed in the wild type is replaced by patches of white interspersed with blotches of orange, brown, and/or black. Piebald is a recessive trait. Crossing a spider, het piebald with either a homozygous piebald or a heterozygous piebald is capable of producing a pastel piebald (Figure 1(b)) in which the piebald pattern and color are further diluted by pastel.

Considering other morphs besides piebald (Figures 1(b), 1(e), and 1(i)), one can observe that the wild type pattern (Figure 1(d)) can be modified by a number of morphs, as seen in Figures 1(a), 1(f), 1(c), 1(j), and 1(h). Similarly, the relatively dark pigments of the wild type coloration and pattern are lightened (as in Figures 1(g), 1(h), and 1(f)) whereby the darker colors are replaced with lighter browns, yellows, oranges, and grays.

The spider morph (Figure 2(a)) causes a loss of lateral pigment, as shown in the figure. Interestingly, when spider is crossed onto piebald (Figure 1(d)), the resulting animal is almost entirely white and only retains the spider type coloration and pattern most cranially with some animals only having nonwhite coloration and pattern on top of their heads. The interaction of spider and piebald appears to produce animals that are almost entirely white. Figure 1(b) shows the Clown morph, which is recessive and affects both color and pattern. Note the yellow pigments and reduced dark regions which are localized dorsally. This particular animal exhibits considerable reduction in its pattern compared to other Clown morphs. It may be the case that this animal also contains allelic variation in another gene which may contribute to the reduced pattern.

The animal in Figure 2(c) is heterozygous for Enchi and heterozygous for Lesser. Altogether, this combination lightens the color and also modifies the pattern. Notice how the wild type animal (Figure 1(d)) has the pattern laterally but fails to exhibit the shaped pattern crossing over the spine. In contrast, the Enchi morph results in the lateral pattern crossing over the spine and forming a continuous shape with the lateral patterns.

The phenotypic plasticity of color and pattern morphs has contributed substantially to the rapid popularity of the ball python as a pet. Interestingly, some morphs are associated with an increased risk for neurological signs, such as the spider morph. Other morphs have produced offspring with ocular abnormalities and craniofacial phenotypes. Additionally, reports of animals with abnormal spinal morphology underscore the importance in gaining a better understanding of the genetics underlying color and pattern variability in this species. Currently, it is not clear which specific undesirable traits are caused by the alleles responsible for the color and pattern variation and which undesirable traits are the result of linked genes,

Another consideration in ball python breeding programs is the decreased time needed to produce double or triple homozygous recessive morphs when breeders choose to back cross offspring and breed siblings together. Such practices can simultaneously decrease the time to produce complex morphs and also increase the frequency of desired genotypes in the offspring. However, continued inbreeding results in a loss of heterozygosity and over time, without intending to, breeders may be faced with triple or quadruple recessive morphs which suffer from a number of undesirable traits resulting from autosomal recessive disease alleles acquired through multiple generations of inbreeding.

Unfortunately, attempting to breed out such disease alleles in ball python lines bred specifically for homozygosity at three, four, or even five loci will prove quite challenging, unless the animals are outbred, and the resulting multilocus homozygous recessive phenotypes will be lost.

Increasing knowledge of the specific genetic mechanisms underlying ball python development and coloration will inevitably lead to enhanced genetic tools and resources for improving breeding programs and actively reducing the frequency of undesirable phenotypes in subsequent generations. Although the identification of alleles responsible for encoding specific color and pattern phenotypes in ball pythons is of interest to breeders and the research community, the goal of this paper is to utilize a bioinformatics based comparative genomics approach to identify genomic sequences likely to underlie aspects of wild type python pigmentation phenotypes. As a first step in investigating the genetic regulation of pigmentation in pythons, it is worthwhile to try and determine whether some of the same genes and transcriptional regulatory elements are conserved between pythons and mammals.

### 3.2. Python Orthologs of Mammalian Coat Color Genes

A set of mammalian coat color associated genes with known pleiotropic effects were identified from the literature [15] and used to obtain the corresponding python orthologous genes. In total, 23 python protein coding genes were identified (Table 1). These genes are known to modulate stripping, darkening, white spotting, color dilution, hypopigmentation, albinism, diluted skin, silver hair, merle coat patterns, leopard complex spotting, and modification of spot size. Additionally, these genes effect eye pigmentation in mammals.

Eye-related phenotypes include dark eyes, ocular hypopigmentation, and iridis, as well as black, blue, hazel, red, and dilute eye colors. Numerous genes within this set shift the balance between light and dark pigmentation within the eye. It is not surprising that phenotypic variation in ocular pigmentation can give rise to the undesirable phenotypes of pigment disturbances, heterochromia, reduced pigmentation patches, and both transparent and dark iris phenotypes.

Because we ultimately want to understand the relationship between color variation and health versus disease, we next explored the pleiotropic effects these genes exert on the rest of the body. Because these genes are widely expressed in multiple cell types, tissues, organs, and body systems, the phenotypic impact of mutations within this gene set is not limited to color variation. In fact, the phenotypes affect sensory organs and nerves, behavior, immune function, tumorigenesis, fitness, reproduction, and metabolism.

Clinically relevant phenotypes have been described in mice exhibiting diluted pigmentation phenotypes. Ocular hypopigmentation is associated with altered ocular development and visual physiology. Diluted eye color contributes to altered iris patterns which correlate with eye diseases. Oculocutaneous albinism (OCA) occurs when mutations in the tyrosinase gene (TYR) lead to oculocutaneous albinism type 1 (OCA1) giving rise to nystagmus and hypoplasia as well as decreased visual acuity.

Phenotypes caused by the genes endothelin 3 (END3), endothelin receptor B (EDNRB), microphthalmia-associated transcription factor (MITF), paired box 3(PAX3), SRY-box 10 (SOX10), and snail homolog 2 (SNAI2) can result in decreased melanocytes, white coat color, and ultimately deafness. Because these genes play important roles in cell differentiation and development, homozygous white animals are frequently nonviable. Horses that are homozygous for the overo mutation in EDNRB exhibit a white coat phenotype and an absence of ganglia of the myenteric plexus. Foals typically develop colic at birth and die within 48 hours.

Reproductive phenotypes are associated with genetic variation in both the KIT receptor and the KIT ligand. Decreased function of these genes results in deficiencies of melanogenesis, hematopoiesis, and gametogenesis. The KIT genes are associated with the piebald phenotype in mammals. Male mice exhibiting a dominant white spotting coat color phenotype are sterile. Similarly, homozygous roan cattle have a white coat color and females lack Mullerian ducts.

An autosomal recessive disease, Chediak-Higashi syndrome (CHS), occurs in humans, American mink, fox, mouse, rat, cattle, cats, orca, tiger, and American bison. The disease is associated with oculocutaneous hypopigmentation, photophobia, increased susceptibility to infections, and bleeding disorders. Immune related phenotypes can arise through mutations in lysosomal trafficking regulator (LYST) and RAS oncogene family member 27A (RAB27A).

Metabolic disorders are also associated with pigmentation phenotypes. The dalmatian urinary metabolism phenotype may have been maintained in the breed through selection for the dalmatian's spots. Another example of metabolic disease arising from these genes was discovered in yellow mice produced from a dominant mutation in agouti signaling protein (ASIP) which is responsible for controlling the relative amounts of eumelanin (dark pigment) and pheomelanin (yellow pigment) produced via signaling through the melanocortin 1 receptor (MC1R). Mice with the ASIP mutation had a yellow coat color but also suffered from obesity, hyperglycemia, hyperinsulinemia, an increased susceptibility to hyperplasia and carcinogenesis, and ultimately lethality.

It is important to note that this relatively small set of important genetic pigmentation mediators are also implicated in a large number of clinically important phenotypes ranging from sensory defects to immune dysfunction and encompassing reproduction, cancer susceptibility, and metabolism. Unlike mammals, which are relatively well studied and well cared for as pets, reptiles in general, and snakes in particular, are likely to be significantly underdiagnosed for the types of undesirable phenotypes associated with these genes. Very few snake owners routinely run clinical pathology tests to assess their snake's health during routinely scheduled wellness exams. Furthermore, ball pythons evolved to be solitary subterranean creatures that, accordingly, spend the majority of their lives in isolation from their owners/breeders. Subsequently, even if one were aware of the presenting signs associated with python metabolic, sensory, reproductive, or immunological disorders, it would be a truly rare occasion when an owner or breeder brought his snake to the veterinarian to address such a disorder.

### 3.3. Analysis of Orthologous Coat Color Protein Sequence Identity across Species

Since our goal in this project is to identify genomic signals associated with conserved transcriptional regulatory networks between mammals and reptiles, we next wanted to assess the extent of conservation between pythons and mammals within these pigmentation associated genes. We were curious to know if these represent highly conserved proteins or fairly divergent ones.

On the one hand, it seems likely that pigmentation genes might represent a highly divergent set of genes. Many animals, including ball pythons, exhibit unique and diverse coloration patterns. However, with the association these genes have with key biological processes, such as nerve formation, reproductive viability, and sensory perception, it is also likely that these genes may be highly conserved across vertebrates.

In order to assess the extent of conservation between mammals and pythons, we identified the orthologous proteins for the 23 python pigmentation genes for human, mouse, dog, horse, cow, chicken, anole lizard, and the garter snake. We were able to identify all 23 orthologs for each species except for the garter snake, for which the orthologs for RAB38 and SLC45A2 were present in the NCBI protein database.

**Figure 3:**
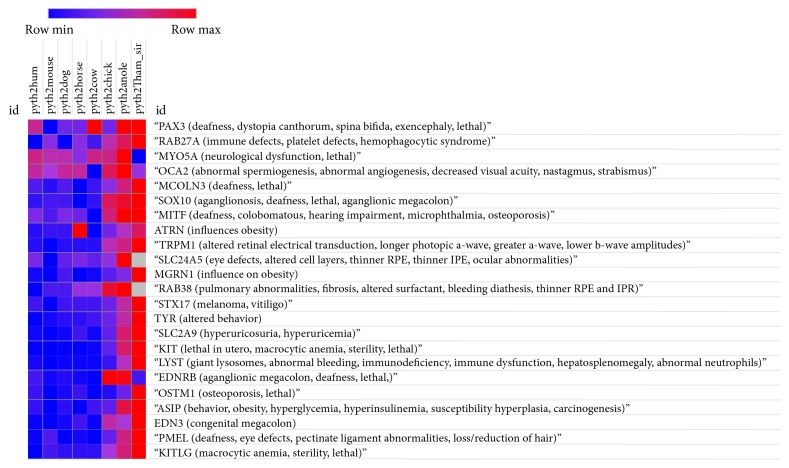
Heat map of orthologous coat color genes illustrating pairwise percent identity. Twenty-three orthologous genes modulating pigmentation and coat color in mammals are shown as a heat map illustrating pairwise protein sequence percent identity based on BLASP best hit between the listed species. The highest percent identity is shown in red, moderate percent identity is shown in magenta, and the lowest percent identity is shown in blue. The pleiotropic effects associated with each gene are included within the parentheses immediately following each gene name. Note: gray boxes (RAB38 and SLC24A5) under pyth2Tham_sirt indicate missing sequences; in this case, the garter snake orthologs of these two genes were not available in the NCBI database. pyth2hum: python and human; pyth2mouse: python and mouse; pyth2dog: python and dog; pyth2horse: python and horse; pyth2cow: python and cow; pyth2chick: python and chicken; pyth2anole: python and anole lizard; pyth2Tham_sirt: python and garter snake.

Pairwise sequence analysis was performed for each orthologous protein between* Python bivittatus* and each of the other species. All percent identity values were recorded (Table 1). Overall, the garter snake exhibits the most pairwise sequence conservation with the python. In order to better visualize the relationships between the orthologous proteins and the species, a heat map was constructed with highly conserved sequences identity values represented in red, while moderate similarity is represented in magenta and divergence is represented by blue (Figure 3).

The most conserved genes among all the species are PAX3 (96%–99%), RAB27A (90%–95%), MYO5A (89%–92%), and OCA2 (83%–93%). In contrast, the most divergent proteins were extracellular secreted ligands for receptors, including ASIP (46%–86%), KITLG (40%–85%), and EDN3 (42%–78%). The extracellular transmembrane glycoprotein, PMEL, exhibited sequence similarity comparable to the extracellular ligands (39% to 77%).

Compared to the secreted ligands, the corresponding membrane bound receptors display considerable more identity. For example, KIT (64%–89%) and EDNRB (64%–79%) exhibit approximately 64% identity between python and mammals but 71% between python and chicken and 83% to 89% among reptiles. EDNRB exhibits comparable patterns of identity with approximately 61% identity between python and mammals and 80% between python and chicken.

The tyrosinase enzyme exhibits slightly lower identity (71%–87%) than the most conserved proteins (RAB27A, PAX3, MYO5A, and OCA2) and is more similar to the transporter SLC2A9 (71% to 87%) than to either ligands or receptors. Based on the pattern we observed across different classes of protein, such as ligands, versus receptors, we were curious to determine if a similar relationship was associated with the undesirable traits associated with these pleiotropic pigmentation genes.

Unlike the striking pattern identified in the types of proteins, the relationship between protein identity and phenotype is not as tightly linked. For example, both KIT receptor and KIT ligand are associated with sterility and lethality; however, the receptor exhibits about 62%–64% identity among the mammals while KIT ligand exhibits approximately 45% identity between the python and mammals. Also, the very highly conserved sequence (MYO5) has an association with lethality, as SLC2A9, although SLC2A9 exhibits almost 10 percentage points less identity than MYO5A.

Nevertheless, the identification of these genes in pythons provides a unique perspective to view undesirable abnormalities produced by breeders. Furthermore, this set of genes represents high confidence candidates to genomic locations of polymorphisms associated with phenotypic variation in coloration.

### 3.4. Identification of Python Pigmentation Gene Expression Network Components

The following gene expression network components were identified:

1. Transcription factors: CREB, FOXD3, LEF-1, MITF, POU3F2, and USF-1.
2. Target gene promoters: AIM1, DCT, MC1R, MITF, MLANA, OA1, PMEL, RAB27A, and TYR.
3. Upstream regions: 1500 bases upstream of the transcription start site.

Because the foundation of our hypothesis is that shared pigmentation physiology and cell biology between pythons and mammals may be detectable as conserved transcriptional regulatory fingerprints in the respective genomes, it is worthwhile to assess the identity of these pigmentation transcription factors across species. Although the particular DNA sequence recognized by a sequence specific transcription factor may diverge in different evolutionary lineages, we reasoned that the extent of divergence between the transcriptional regulatory elements (between the species) may be inversely related to the conservation among the transcription factors (between the species) that recognize those regulatory binding sites. Therefore, we expanded the set of species considered for the alignments of orthologous pigmentation transcription factors to broaden the representation of reptile transcription factor sequences.

### 3.5. Protein Domain Analysis of Python Pigmentation Associated Transcription Factors

In order to most effectively interpret the sequence alignment data, we first identified functionally important domains within each protein (Table 3). Domain location information provides biologically relevant context that is critical when attempting to infer the functional consequences of sequence conservation and/or sequence divergence observed in alignments.

Analysis of CREB protein sequence revealed the presence of a coactivator domain from position 87 to position 146 and a basic-leucine zipper domain spanning amino acids between residue 284 and residue 342. Analysis of FOXD3 identified a winged helix-turn-helix DNA binding domain beginning at position 71 and ending at position 173. Within the LEF-1 sequence, three domains were identified; the first spans residues 1 through 65 and encodes a catenin binding domain. The second LEF-1 domain detected was CTNNB1 binding, N-terminal region beginning at the first residue and ending at position 209. The final functional region detected in LEF-1 was the high mobility group box domain spanning the region from 293 to 375.

MITF contains an N-terminal MiT/TFE transcription factor region from position 4 to position 142 and a MiT/TFE transcription factor C-terminal motif beginning at position 393 and ending at position 519. Additionally, MITF encodes a Myc-type basic helix-loop-helix (bHLH) domain beginning at position 303 and ending at position 370. Domain detection with POU3F2 identified a POU-specific domain from residue 51 through residue 125 and a homeodomain-like region from position 125 to position 205. The USF-1 protein has an Myc-type helix-turn-helix-loop-helix (bHLH) domain beginning at amino acid 193 and continuing until amino acid 286.

### 3.6. Analysis of Orthologous Pigmentation Associated Transcription Factors

**Figure 4:**
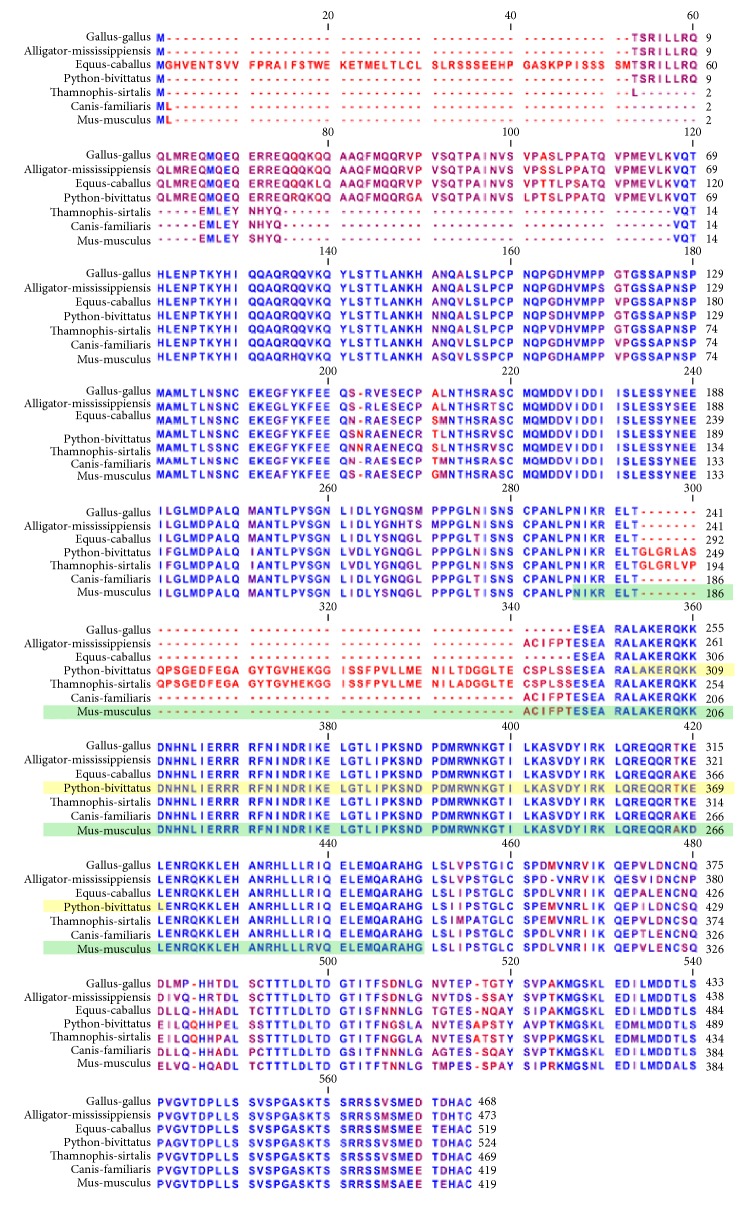
Orthologous multiple sequence alignment of MITF across species. Strong patterns of conservation are observed from alignment position 130 until alignment position 293. A second long block of conservation is observed at position 347 and continues until the end of the alignment at position 575. The DNA binding region of MITF is contained with the Myc-type, basic helix-loop-helix (bHLH) domain at spanning from position 303 to position 370 (this region is highlighted in yellow in the figure). This region of the alignment is extremely well conserved with only a single amino acid differing between the species at alignment position 418 where the mammals (mouse, dog, and horse) have a threonine (T) while all of the nonmammals have an alanine (A). The highlighted green region of the mouse protein indicates the region of the mouse DNA binding region for which a protein structure cocrystal exists.

The MITF alignment (Figure 4) includes three mammalian orthologs (mouse, dog, and horse), three reptilian orthologs (alligator, python, and garter snake), and an avian ortholog (chicken). Strong patterns of conservation are observed from alignment position 130 until alignment position 293. A second long block of conservation is observed at position 347 and continues until the end of the alignment at position 575. The DNA binding region of MITF is contained with the Myc-type, basic helix-loop-helix (bHLH) domain at spanning from position 303 to position 370 (this region is highlighted in yellow in the figure). This region of the alignment is extremely well conserved with only a single amino acid differing between the species at alignment position 418 where the mammals (mouse, dog, and horse) have a threonine (T) while all of the nonmammals have an alanine (A).

**Figure 5:**
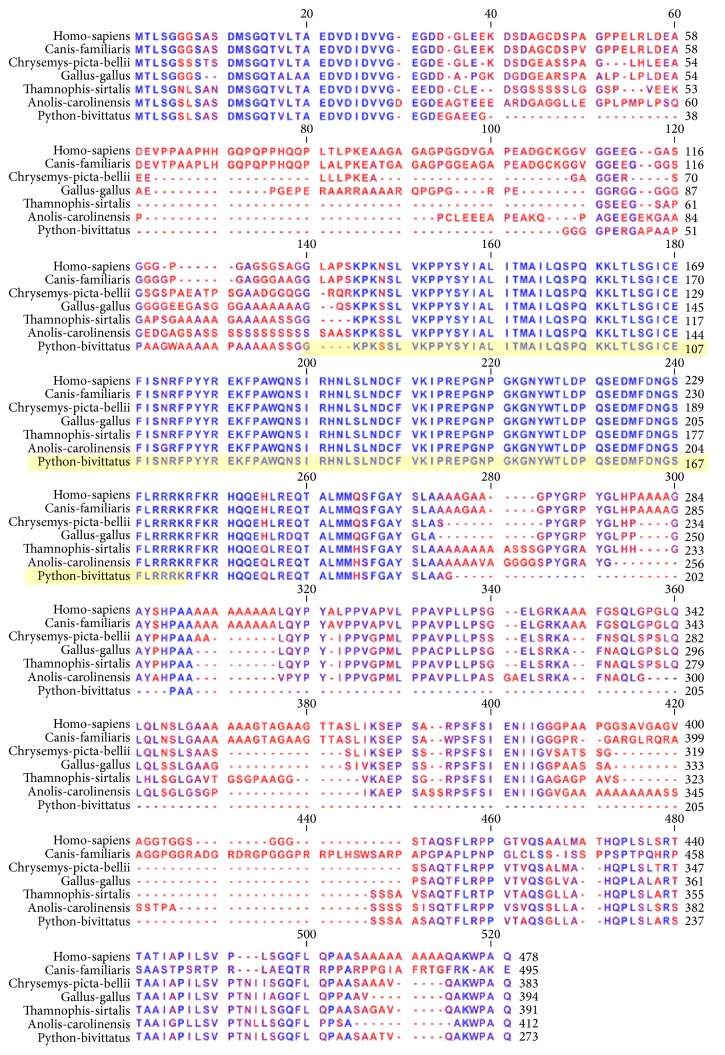
Orthologous multiple sequence alignment of FOXD3 across species. Highly conserved regions are colored in blue. The winged helix-turn-helix DNA binding region spans a region beginning at position 71 and ending at position 173. This region is highly conserved. The single nonconserved residue within the DNA binding domain occurs at position 148 in the alignment where human, dog, and painted turtle have asparagine (N) while chicken, garter snake, anole lizard, and python have serine (S).

The FOX3D alignment (Figure 5) includes two mammals (human and dog), four reptiles (green anole lizard, garter snake, painted turtle, and python), and one avian species (chicken). There are numerous gaps at multiple locations within the alignment. Highly conserved regions are colored blue. The winged helix-turn-helix DNA binding region spans a region beginning at position 71 and ending at position 173. This region is highly conserved. The single nonconserved residue within the DNA binding domain occurs at position 148 in the alignment where human, dog, and painted turtle have asparagine (N) while chicken, garter snake, anole lizard, and python have serine (S).

**Figure 6:**
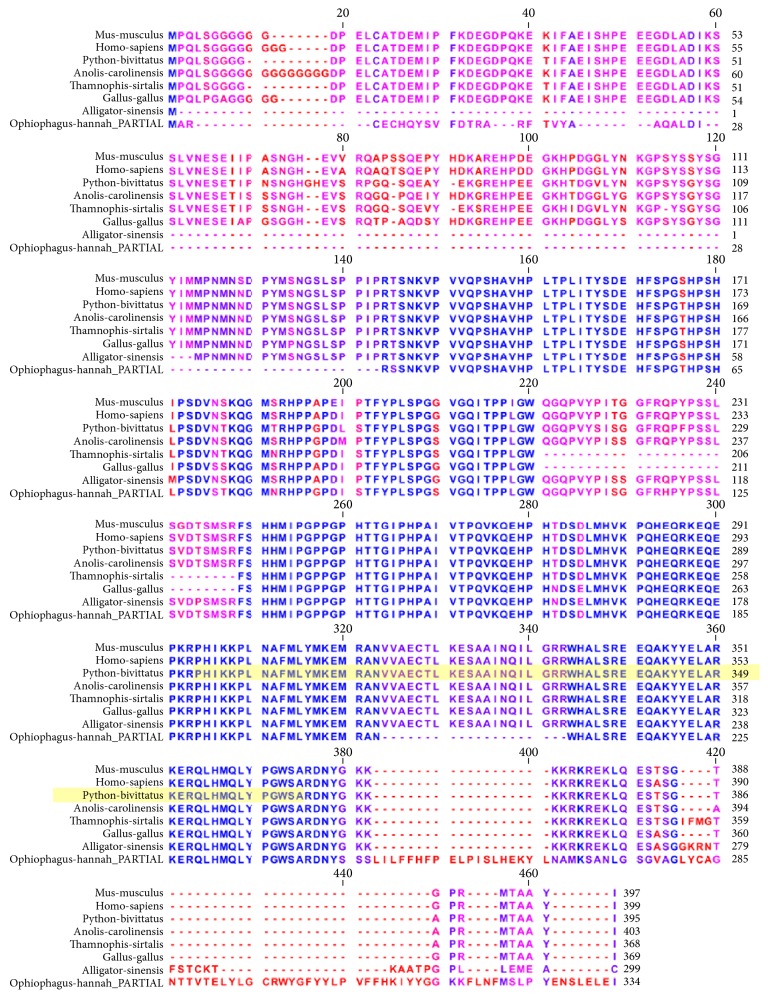
Orthologous multiple sequence alignment of LEF-1 across species. Note regions of divergence as well as some areas of strong conservation. Specifically, the N-terminal region exhibits some variability in amino acids exemplified by variable length repeats of the amino acid glycine near the beginning of the sequences. Stronger patterns of conservation are observable beginning at alignment position 146 and continuing at alignment position 220 which partly overlaps with the CTNNB1 binding, N-terminal domain (position 1 to position 209 in the python sequence). The DNA binding region of LEF-1 exhibits identical amino acid composition among all the species except for the king cobra (for which the sequence is annotated as partial).

Within the LEF-1 alignment (Figure 6), there are regions of divergence as well as some areas of strong conservation. Specifically, the N-terminal region exhibits some variability in amino acids exemplified by variable length repeats of the amino acid glycine near the beginning of the sequences. Stronger patterns of conservation are observable beginning at alignment position 146 and continuing at alignment position 220 which partly overlaps with the CTNNB1 binding, N-terminal domain (position 1 to position 209 in the python sequence). The DNA binding region of LEF-1 exhibits identical amino acid composition among all the species except for the king cobra (for which the sequence is annotated as partial).

**Figure 7:**
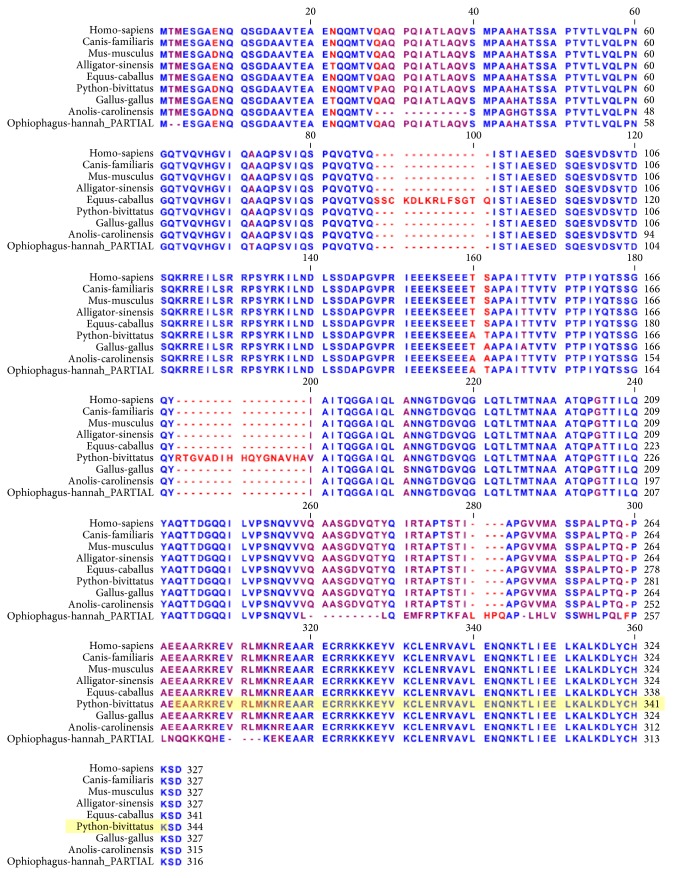
Orthologous multiple sequence alignment of CREB across species. CREB sequences exhibit considerable conservation throughout much of the alignment with a few notable point differences with the conserved regions. The basic-leucine zipper DNA binding domain occurs in the C-terminal region (284 to 342 in the python sequence). Within the DNA recognition strong conservation is observed among the species and only king cobra exhibits considerable divergent sequence identity in this region.

CREB sequences exhibit considerable conservation throughout much of the alignment (Figure 7) with a few notable point differences with the conserved regions. The basic-leucine zipper DNA binding domain occurs in the C-terminal region (284 to 342 in the python sequence). Within the DNA recognition, strong conservation is observed among the species and only king cobra exhibits considerable divergent sequence identity in this region.

**Figure 8:**
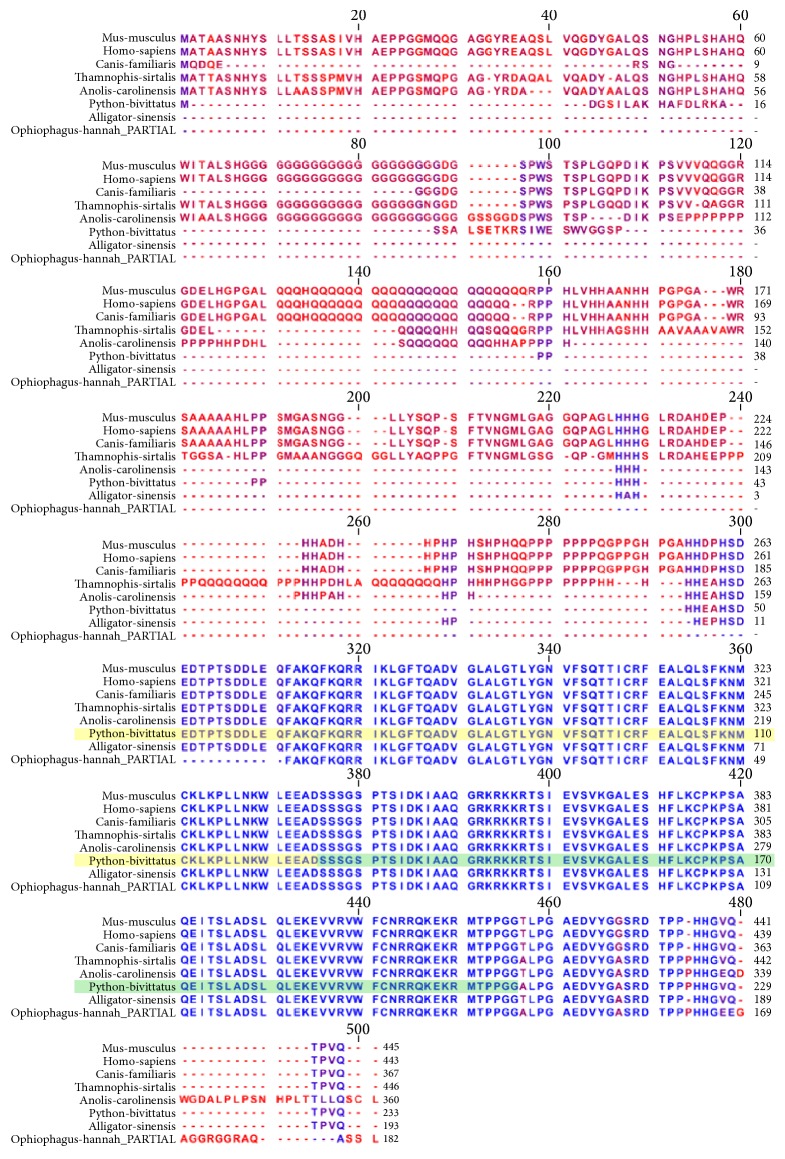
Orthologous multiple sequence alignment of POU3F2 across species. The alignment shows marked divergence within the N-terminal portions of the protein and strong conservation within the C-terminal region. The pattern of sequence variation observed within the N-terminal region is consistent with properties associated with transcriptional activator domains where lengths of consecutive repeats of basic amino acids vary among species. Between alignment position 151 and alignment position 160, a group of glutamines (Q) are observed with noticeable differences among the species. However, the DNA binding region of POU3F2, which is composed of the POU-specific domain (python position 51 to position 125) and the homeodomain (python position 125 to position 205), exhibits perfect sequence identity among all of the species.

The POU3F2 alignment (Figure 8) shows marked divergence within the N-terminal portions of the protein and strong conservation within the C-terminal region. The pattern of sequence variation observed within the N-terminal region is consistent with properties associated with transcriptional activator domains where lengths of consecutive repeats of basic amino acids vary among species. Between alignment position 151 and alignment position 160, a group of glutamines (Q) are observed with noticeable differences among the species. However, the DNA binding region of POU3F2, which is composed of the POU-specific domain (python position 51 to position 125) and the homeodomain (python position 125 to position 205), exhibits perfect sequence identity among all of the species.

**Figure 9:**
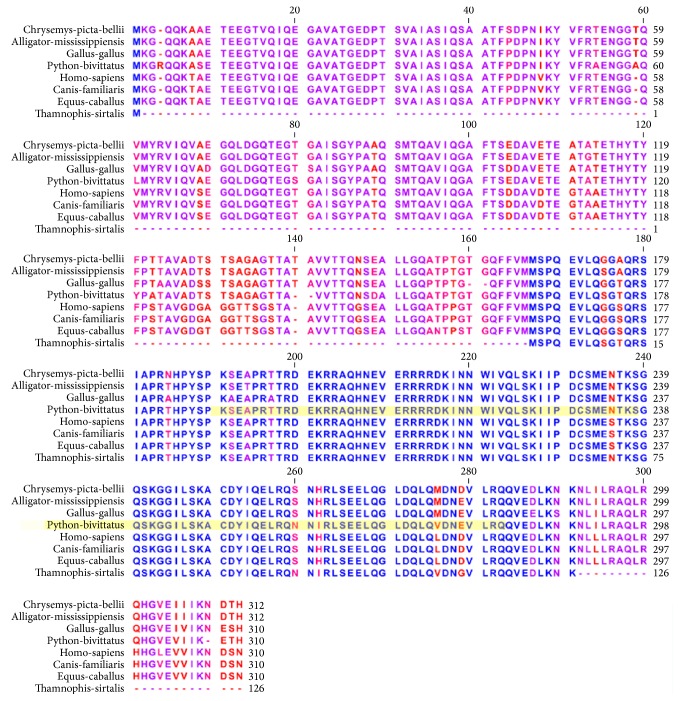
Orthologous multiple sequence alignment of USF-1 across species. The alignment shows patterns of conservation as well as divergence between the species. The N-terminal region exhibits some distinct differences between the species interspersed with groups of strong conservation. The Myc-type, basic helix-loop-helix (bHLH) DNA binding domain spans the region of the python protein beginning at position 193 and ending at position 286. Within this portion of the alignment, eight specific amino acids exhibit divergence between the species.

THE USF-1 alignment (Figure 9) shows patterns of conservation as well as divergence between the species. The N-terminal region exhibits some distinct differences between the species interspersed with groups of strong conservation. The Myc-type, basic helix-loop-helix (bHLH) DNA binding domain spans the region of the python protein beginning at position 193 and ending at position 286. Within this portion of the alignment, eight specific amino acids exhibit divergence between the species.

### 3.7. Evolutionarily Conserved Clusters of Pigment Transcription Factor Binding Sites in Python Genes

The 1500-nucleotide upstream regions immediately adjacent to the transcription start site of python genes were analyzed to identify evolutionarily conserved transcription factor binding sites associated with gene expression regulation of pigment related transcripts.

**Figure 10:**
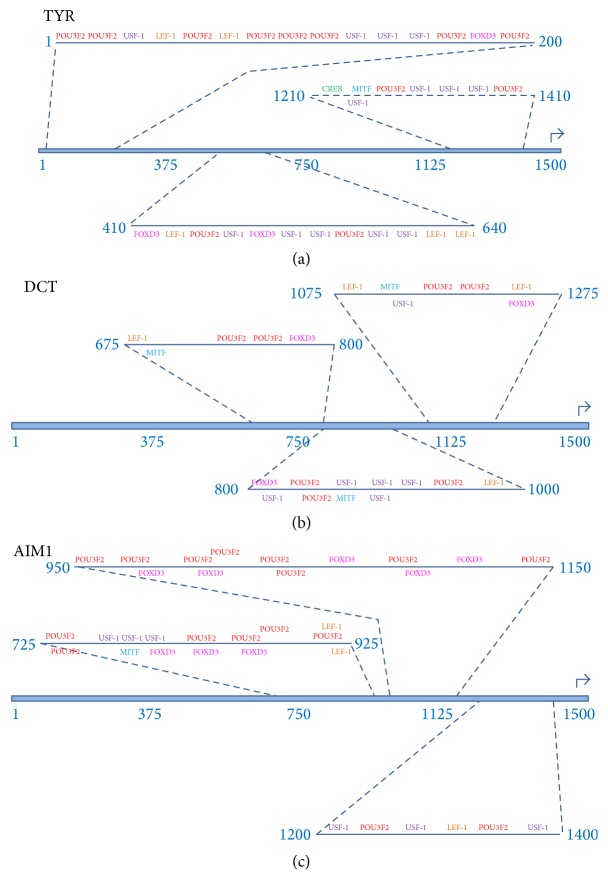
TYR, DCT, and AIM1 upstream regions and associated candidate enhancer modules. (a) The upstream region from the python tyrosinase gene (TYR) contains a cluster of pigmentation associated transcription factor binding sites located between position 1 and position 200. This cluster includes binding sites for POU3F2, USF-1, LEF-1, and FOXD3. A second cluster occurs between position 410 and position 640 that contains sites for FOXD3, LEF-1, USF-1, and POU3F2. The third cluster is located from position 1210 to position 1410 and contains binding sites for CREB, MITF, POU3F2, and USF-1. (b) The upstream region of python dopachrome tautomerase (DCT) contains a cluster of binding sites from position 675 to position 800 that include LEF-1, POU3F2, MITF, and FOXD3. A second cluster occurs from position 800 to position 1000 which includes binding sites for transcription factors FOXD3, POU3F2, USF-1, LEF-1, and MITF. The third cluster contains binding sites for LEF-1, MITF, USF-1, POU3F2, and FOXD3. (c) The upstream region from Absent In Melanoma 1 (AIM1) (Figure 10(c)) contains a cluster of transcription factor binding sites between position 725 and position 925 for POU3F2, USF-1, MITF, FOXD3, and LEF-1. A second cluster of sites beginning at position 950 and ending at position 1150 contains binding sites for POU3F2 and FOXD3. A third cluster of binding sites beginning at position 1200 and ending at position 1500 has binding sites for USF-1, POU3F2, and LEF-1.

The upstream region from the python tyrosinase gene (TYR) (Figure 10(a)) contains a cluster of pigmentation associated transcription factor binding sites located between positions 1 and 200. This cluster includes binding sites for POU3F2, USF-1, LEF-1, and FOXD3. A second cluster occurs between position 410 and position 640 which contains sites for FOXD3, LEF-1, USF-1, and POU3F2. The third cluster is located from position 1210 to position 1410 and contains binding sites for CREB, MITF, POU3F2, and USF-1. Although a CREB binding site was identified, the dissimilarity score is relatively high (see the supplemental files) suggesting that perhaps it may not be a true site. Prior analysis of the human tyrosinase gene has characterized a cAMP dependent increase in expression, but the kinetics of the response suggest the cAMP induced response occurs via the CREB activity on MITF promoters, which ultimately increases MITF protein levels facilitating increased expression of tyrosinase through the MITF binding site in the tyrosinase upstream region [25].

The upstream region of python dopachrome tautomerase (DCT) (Figure 10(b)) contains a cluster of binding sites from position 675 to position 800 which include LEF-1, POU3F2, MITF, and FOXD3. A second cluster occurs from position 800 to position 1000 which includes binding sites for transcription factors FOXD3, POU3F2, USF-1, LEF-1, and MITF. The third cluster contains binding sites for LEF-1, MITF, USF-1, POU3F2, and FOXD3.

The upstream region from Absent In Melanoma 1 (AIM1) (Figure 10(c)) contains a cluster of transcription factor binding sites between position 725 and position 925 for POU3F2, USF-1, MITF, FOXD3, and LEF-1. A second cluster of sites beginning at position 950 and ending at position 1150 contains binding sites for POU3F2 and FOXD3. A third cluster of binding sites beginning at position 1200 and ending at position 1500 has binding sites for USF-1, POU3F2, and LEF-1.

**Figure 11:**
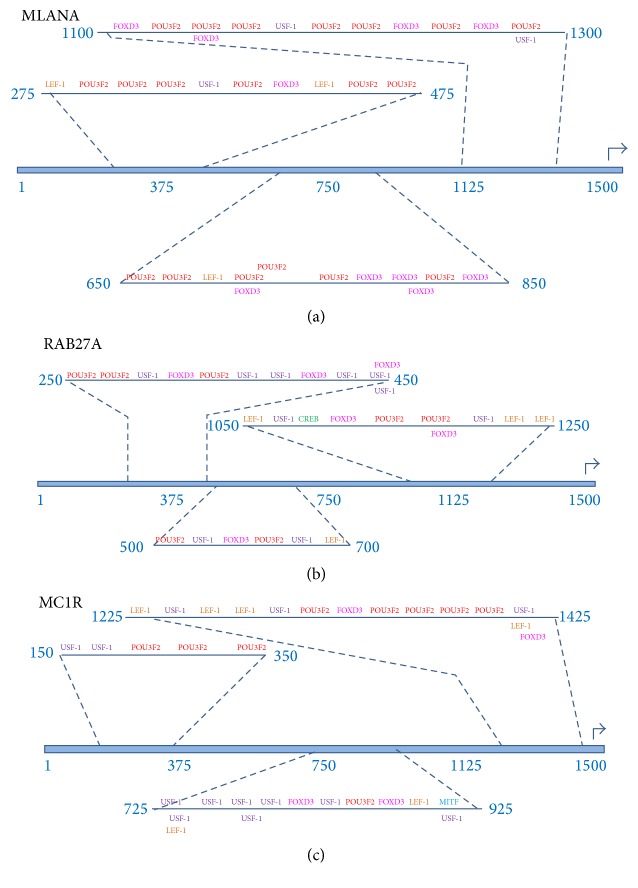
MLANA, RAB27A, and MC1R upstream regions and associated candidate enhancer modules. (a) The upstream region from melan-A (MLANA) contains a cluster of binding sites beginning at position 275 and ending at position 475 which include recognition sites for LEF-1, POU3F2, USF-1, and FOX3D. A second cluster of binding sites begins at position 650 and extends to position 850 which contains binding sites for POU3F2, LEF-1, and FOX3D. The third cluster of sites occurs between position 1100 and position 1300 which contains binding sites for FOXD3, POU3F2, and USF-1. (b) The upstream region for RAS oncogene family member 27A (RAB27A) (Figure 11(b)) has a cluster of transcription factor binding sites beginning at position 250 and ending at position 450 which include sites for POU3F2, USF-1, and FOXD3. The second cluster of binding sites begins at position 500 and ends at position 700 which contains sites for POU3F2, USF-1, FOX3D, and LEF-1. The third cluster begins at position 1050 and ends at position 1250 and binds LEF-1, USF-1, CREB, FOX3D, and POU3F2. (c) The upstream region for melanocortin 1 receptor (MC1R) contains a cluster of transcription factor binding sites for USF-1 and POU3F2 between position 150 and position 350. A second cluster begins at position 725 and ends at position 925 with binding sites for USF-1, LEF-1, FOX3D, POU3F2, and MITF. A third cluster of binding sites is located between position 1225 and position 1425 with sites for LEF-1, USF-1, POU3F2, and FOXD3.

The upstream region from melan-A (MLANA) (Figure 11(a)) contains a cluster of binding sites beginning at position 275 and ending at position 475 which include recognition sites for LEF-1, POU3F2, USF-1, and FOX3D. A second cluster of binding sites begins at position 650 and extends to position 850 which contains binding sites for POU3F2, LEF-1, and FOX3D. The third cluster of sites occurs between position 1100 and position 1300 which contains binding sites for FOXD3, POU3F2, and USF-1.

The upstream region for RAS oncogene family member 27A (RAB27A) (Figure 11(b)) has a cluster of transcription factor binding sites beginning at position 250 and ending at position 450 which include sites for POU3F2, USF-1, and FOXD3. The second cluster of binding sites begins at position 500 and ends at position 700 which contains sites for POU3F2, USF-1, FOX3D, and LEF-1. The third cluster begins at position 1050 and ends at position 1250 and binds LEF-1, USF-1, CREB, FOX3D, and POU3F2.

The upstream region for melanocortin 1 receptor (MC1R) (Figure 11(c)) contains a cluster of transcription factor binding sites for USF-1 and POU3F2 between position 150 and position 350. A second cluster begins at position 725 and ends at position 925 with binding sites for USF-1, LEF-1, FOX3D, POU3F2, and MITF. A third cluster of binding sites is located between position 1225 and position 1425 with sites for LEF-1, USF-1, POU3F2, and FOXD3.

**Figure 12:**
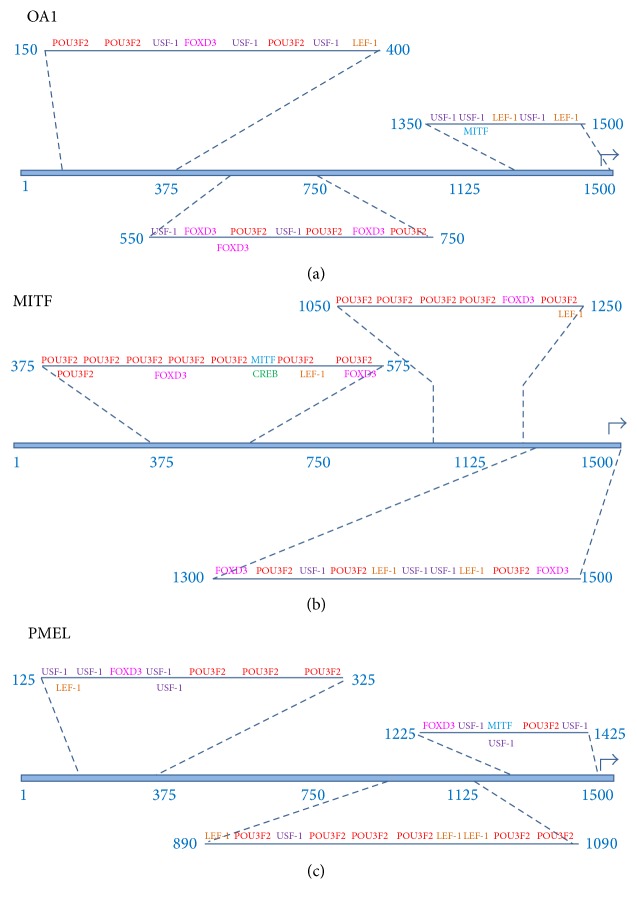
OA1, MITF, and PMEL upstream regions and associated candidate enhancer modules. (a) The upstream region for ocular albinism type 1 (OC1) contains a cluster of transcription factor binding sites beginning at position 150 and spanning to position 400 which include recognition sites for POU3F2, USF-1, FOX3D, and LEF-1. A second cluster of binding sites occurs between position 550 and position 750 which binds USF-1, FOX3D, and POU3F2. The third cluster of transcription factor binding sites begins at position 1350 and ends at position 1500 and recognizes transcription factors USF-1, MITF, and LEF-1. (b) The upstream region for microphthalmia-associated transcription factor (MITF) has a cluster of transcription factor binding sites beginning at position 375 and ending at position 575. The sites recognized within this first cluster include POU3F2, FOXD3, MITF, CREB, and LEF-1. A second cluster of binding sites starts at position 1050 and ends at position 1250 with recognition sites for POU3F2, FOX3D, and LEF-1. The third cluster of binding sites begins at position 1300 and extends to position 1500 which recognizes transcription factors FOXD3, POU3F2, USF-1, and LEF-1. (c) The upstream region for premelanosome protein (PMEL) contains a cluster of transcription factor binding sites between position 125 and position 325 that include sites for USF-1, FOX3D, and LEF-1. A second cluster of binding sites begins at position 890 and extends to position 1090 with recognition sites for LEF-1, POU3F2, and USF-1. The third cluster of transcription factor binding sites begins at position 1225 and ends at position 1425 and contains binding sites for FOXD3, USF-1, and MITF.

The upstream region for OA1-melanosome membrane protein (OA1) (Figure 12(a)) contains a cluster of transcription factor binding sites beginning at position 150 and spanning to position 400 which include recognition sites for POU3F2, USF-1, FOX3D, and LEF-1. A second cluster of binding sites occurs between position 550 and position 750 which binds USF-1, FOX3D, and POU3F2. The third cluster of transcription factor binding sites begins at position 1350 and ends at position 1500 and recognizes transcription factors USF-1, MITF, and LEF-1.

The upstream region for microphthalmia-associated transcription factor (MITF) (Figure 12(b)) has a cluster of transcription factor binding sites beginning at position 375 and ending at position 575. The sites recognized within this first cluster include POU3F2, FOXD3, MITF, CREB, and LEF-1. A second cluster of binding sites starts at position 1050 and ends at position 1250 with recognition sites for POU3F2, FOX3D, and LEF-1. The third cluster of binding sites begins at position 1300 and extends to position 1500 which recognizes transcription factors FOXD3, POU3F2, USF-1, and LEF-1. The presence of a MITF binding site within the upstream region of the MITF coding region suggests that MITF may regulate its own expression in a positive feedback manner. Self-activation of MITF has been described in human cell lines [14].

The upstream region for premelanosome protein (PMEL) (Figure 12(c)) contains a cluster of transcription factor binding sites between position 125 and position 325 which include sites for USF-1, FOX3D, and LEF-1. A second cluster of binding sites begins at position 890 and extends to position 1090 with recognition sites for LEF-1, POU3F2, and USF-1. The third cluster of transcription factor binding sites begins at position 1225 and ends at position 1425 and contains binding sites for FOXD3, USF-1, and MITF.

## 4. Discussion

Pigmentation phenotypes have been produced through both natural selection and artificial selection. Naturally occurring color and pattern variation provide advantages for organisms that include thermoregulation, camouflage, mimicry, sexual fitness, and warnings. Naturally selected pigmentation variation in domestic animals offers a breadth of morphological phenotypic variation that can be used to produce visually appealing lineages and breeds. Undesirable health traits associated with perturbations in some pigmentation genes have been observed in certain domesticated species bred for particular color and pattern phenotypes.

Moreover, malignant melanoma arises from the neoplastic transformation of melanocytes and results in the most aggressive form of skin cancer. As of 2004, the incidence was 27.5/100,000 for Caucasian Americans and less than half that for African Americans. Although early surgical intervention cures 90% of patients, metastatic melanoma is resistant to treatment. The five-year survival rate for patients in stage III ranges from 78% to 48%, and for M1 patients, the prognosis is even worse, with a 1-year survival rate between 62% and 33% [26].

As of 2013, the incidence of melanoma had grown to 5% of the population. A number of pharmacological treatments target inhibition of BRAF, MEK, c-KIT, and PI3K pathways. BRAF is a serine/threonine kinase involved in the RAS-RAF-MEK-ERK signaling cascade which promotes proliferation and antiapoptotic cellular programs. Mutations in BRAF and N-RAS account for roughly 50% and 20% of melanoma patients, respectively. MEK is a component of the MAPK signaling cascade which also promotes proliferation and migration. c-KIT mutations and amplifications are potent drivers of melanocyte tumorigenesis. Phosphatidylinositol-3-kinase (PIK3) is ectopically activated in most metastatic melanomas [27]. Altogether, these four targets represent viable molecular strategies for treating melanoma.

Cancer is responsible for approximately 15% of human deaths worldwide each year. In wildlife, trauma and starvation have been considered the leading causes of death. However, more recently, the impact of cancer on wildlife has become more recognized. For example, endangered reptiles, such as the green sea turtle (*Chelonia mydas*), are threatened by herpes-associated fibropapillomatosis, as the endangered leather back turtle (*Dermochelys coriacea*), Kemp's ridley turtle (*Lepidochelys kempii*), and the hawksbill turtle (*Eretmochelys imbricata*) [27].

Neoplasia in reptiles was once thought to be uncommon, but in the last decade this view has evolved. Prevalence is the highest in snakes (15%), followed closely by lizards (9%) and then chelonians (3%), and is the lowest in crocodiles (2%). Out of 43 different types, the top 5 are lymphoma (11%), soft tissue sarcoma (11%), adenocarcinoma (8%), fibrosarcoma (6%), and melanoma (6%). Among snakes, melanoma prevalence has been characterized across colubrids (2%), crotalids (1%), boids (0.5%), and vipers (0.5%) [28].

Tumors associated with pigment cells of skin tend to be rarely reported in reptiles. One recent study from 2012 characterized the frequency of chromatophoromas in an archive of 179 reptilian tumors. The results identified a frequency of 15% across all reptiles, with melanophoromas representing 11% while iridophoromas had slightly over 3%. When compared across species, lizards exhibited the highest frequency with 20%, corresponding to 14% melanophoromas and 6% iridophoromas. Snakes had the second highest frequency (10%) with all being melanophoromas, followed by turtles/tortoises (8%), also having all melanophoromas [29].

Reptiles are less well studied than mammals. Increasing knowledge of the shared physiological and molecular mechanisms underlying important phenotypes is valuable. Comparative genomics approaches allow for knowledge gained in one species to be leveraged for use in another species. Our working hypothesis for this study is that* shared pigmentation biology between species can be identified through the detection of genomic fingerprints associated with conserved transcriptional regulatory networks underlying melanocyte biology and melanin formation.* The idea that conserved physiology will be encoded in conserved genomic signals is the foundation of comparative genomics.

Since melanocytes are conserved cell types in both mammals and reptiles, we hypothesized that the transcriptional regulatory mechanisms would be conserved for numerous cellular processes common to melanocytes such as melanocyte differentiation, melanosome formation, regulation of enzymes involved in pigment biosynthesis, and melanocyte signaling pathways. Because many of the mammalian genes involved in these processes have corresponding orthologs in the Burmese python, it seemed plausible that some of the transcriptional regulation of these genes would also be shared between mammals and pythons.

The underlying principle for eukaryotic gene activation is cooperativity whereby nucleosomes inactivate chromatin and transcriptional activators activate chromatin. This “chromatin switch” provides a molecular regulatory mechanism for gene level transcriptional control. The switch between active and inactive state is determined by the competition between the factors contributing to the active and inactive states. In this model, enhancers are contiguous DNA sequences containing binding sites for transcriptional activators and thereby enable specific combinations of transcription factors to flip the “chromatin switch” towards gene activation [30].

Because transcription factor binding sites are much shorter than full length amino acid and nucleotide sequences, identifying evolutionarily conserved binding sites is not effectively accomplished using sequence alignment methods, as is typically done with proteins. Moreover, the fact that transcription factors can recognize a variety of consensus binding site perturbations contributes to the difficulty in successfully identifying true positive binding sites. Additionally, selective pressures acting on the two species since their divergence from a common ancestor may have resulted in corresponding changes in the transcription factor DNA binding domains resulting in correlated divergence in the transcription factor binding site sequences in the upstream regions of genes regulated by these transcription factors.

Identifying enhancers in the python genome represents a cryptographic approach to decoding biological information. Specifically, the function of mammalian melanocyte associated transcription factors was employed to identify multiple binding sites clustered within a relatively small interval in the upstream region of a particular gene within the python genome. These mammalian transcription factors and associated target genes provide the “language of the cipher” needed to decode the evolutionarily conserved melanocyte biology encoded in the reptile genome [31].

Our strategy for identifying genomic signals associated with python pigmentation biology parallels the method described by Bodnar and Bradley to decode the genetic program underlying developmental phenotypes during drosophila embryogenesis. Although our end product differs substantially from the integrated developmental maps characterizing gene regulation of development, our result integrates a variety of comparative genomic and published knowledge to produce an initial model of portions of the gene expression network underlying pigmentation phenotypes in the python [32].

The total number of indexed publications in PubMed associated with “*pigmentation OR melanocyte*” as of 2016 is 54,319, with 9,254 publications specifically containing “*gene OR genetic OR genome OR genomics*” in the title or abstract. The information content of this dataset is overwhelming and so complex that it is not possible to use in its entirety. However, a workable approach was adopted as described by Bodnar which provides experimentalists with testable hypotheses about pigmentation and melanocyte genes, upstream regions, transcription factors, cis elements, and putative enhancers [33].

One method for increasing the signal to noise ratio in detection of regulatory binding sites within noncoding regions of the genome is to limit the search to those regions in close proximity to the transcription site of a gene. Although this can facilitate the identification of true positive transcription factor binding sites, it also causes an increase in false negatives due to the true binding sites that are not detected which lie outside of the search region. Another method for increasing true regulatory motifs is to look for combinations of transcription factor binding sites. Since transcription factor binding sites are relatively small, on the order of 4 to 20 nucleotides each, the search for combinations of binding sites adds the benefit of increased specificity of detection through the reduced frequency associated with the reduced joint probability of finding a combination of sites with close proximity of one another.

Further success in identifying true regulatory motifs in genomic sequence can be accomplished by limiting the search to transcription factors that are known to regulate or contribute to the regulation of genes. This also suffers from the production of false negatives because transcription factors not considered in the search will not end up in the results.

Ultimately, the specific methodological approach used to identify cis-regulatory elements in genomes will be informed by the specific overarching goal of the project and the subsequent anticipated downstream applications of the data. For example, a goal of identifying any possible transcription factor binding site within a genomic sequence is quite different from the goal of identifying at least one highly likely true transcription factor binding site in a genomic sequence. When considering downstream applications of the predicted data, it is important to consider the associated time and cost of validating predictions which is directly proportional to the number of prediction and affected by the prediction success rate.

For the purpose of investigating the genetic basis of common pigmentation and morphological pattern phenotypes that may be shared between mammals and pythons, we were much more interested in identifying fewer candidates with very high true positive rates rather than massive numbers of predictions that are dominated by false positive rates. Subsequently, we used a variety of constraints that helped shift the outcome towards true positives with the trade-off of identifying fewer numbers of candidates and also having a relatively high rate of false negatives (binding sites we failed to predict which are in fact real).

We believe that the constraints we imposed shifted our predictions towards our desired goal of producing a higher true positive rate in a smaller set of total predictions. First, we selected as starting orthologous python genes, only genes for which known coat color and pattern phenotypes were known to occur in other species (Table 1). Additionally, we specifically limited our search of potential regulatory regions to only the most proximal 1500 nucleotides immediately upstream of genes. Furthermore, we specifically limited our transcription factors of interest to those with a known role in melanocyte biology from studies in mammals (Table 2). Moreover, we looked for clusters of these transcription factor binding sites within relatively small intervals (~200 nucleotides) of the upstream region. Finally, we limited our predictions to only three putative enhancer regions per upstream region.

Together, these constraints have produced a set of 27 candidate enhancer modules likely to underlie pigmentation and pattern phenotypes in pythons. While bioinformatics approaches ultimately produce predictions, for which subsequent experimental validation must be performed, we noted a number of features in our predictions suggesting some of them are true positive regulatory control elements driving expression of python genes contributing to the types of color patterns observed in ball pythons. Specifically, many of our clustered transcription factor binding sites contain multiple instances of the same transcription factor in tandem [34, 35], which is a known transcriptional regulatory feature associated with evolution of promoters [36, 37]. Additionally, some of our candidate enhancers contain binding sites for three, four, and even five pigmentation transcription factors in ~200-nucleotide intervals. The joint probability of such an occurrence is low and therefore provides support for the idea that some of our predictions are in fact true positives. Our enhancer module results are consistent with the combinatorial control of eukaryotic gene expression [38, 39] whereby synergistic interactions between different transcription regulators occur in an enhanceosome in order to achieve activation of target genes [40, 41].

The results of our work provide a starting frame work for investigating the genetic regulation of python color and pattern variation. In combination with the hundreds of color/pattern variant lines of ball pythons, our set of color/pattern enhancer modules creates an ideal starting point to investigate the genetic factors contributing to observed color and pattern variation in pythons. Although genes and alleles contributing to the specific phenotypes observed in the ball python may lay outside the set we have described, our work implicates a number of genes as likely to be involved in python melanocyte biology.

Another important application of our work is a better understanding of the anticipated undesirable phenotypes that may be associated with specific ball python color variants based upon the pleiotropic effect these genes play in biology (Figure 3).

The results of this study should be interpreted cautiously based on the fact that there are hundreds of genes involved in pigmentation, for which subtle and complex regulatory mechanisms have yet to be elucidated. Moreover, the regulatory elements underlying eukaryotic gene expression are distributed across very large landscapes that may cover thousands and even millions of bases along a chromosome. Therefore, further experimental investigations into our predictions are necessary to not only validate each prediction but also relate each cluster of evolutionarily conserved transcription factor binding sites to its regulatory purpose and association (if any) with specific wild type python color/pattern phenotypes. Ultimately, through future studies, the genetic correlates of ball python color and pattern morphs may be elucidated.

## 5. Conclusion

We describe a novel application of bioinformatics to infer conserved cellular genetic programs in melanocyte biology based on comparative genomics, supervised literature mining, and regulatory motif analysis resulting in the identification of 27 candidate enhancer modules, within 9 orthologs of mammalian pigmentation genes likely to contribute to pigmentation and pattern phenotypes in pythons. Our results focused on a set of 6 transcription factors that have been previously demonstrated to modulate melanocyte and pigmentation in mammals. The integration of supervised literature mining, phenotypic characterization of pigmentation in pythons, comparative genomics, orthologous protein sequence analysis, phylogenetic analysis, sequence similarity, detection of TF binding sites, clustering of TF binding sites, and regulatory module identification provides an example of an integrated bioinformatics framework for extending biological knowledge from one species to another. Specifically, we searched for CREB, FOXD3, LEF-1, MITF, POU3F2, and USF-1 binding sites within the 1500-nucleotide upstream regions of AIM1, DCT, MC1R, MITF, MLANA, OA1, PMEL, RAB27A, and TYR. Our results provide initial evidence for aspects of conserved melanocyte and pigmentation biology between mammals and reptiles. Among our findings, we identified binding sites for the MITF transcription factor in upstream regions for DCT, TYR, AIM1, MC1R, MITF, PMEL, and OA1. Interestingly, we detect evidence of a MITF positive feedback circuit in reptiles, as has been characterized in mammals. We briefly review the prevalence of melanoma in both humans and reptiles, with a particular focus on snakes. Overall, our findings provide a set of computationally inferred transcriptional network models for melanocyte mediated pigmentation biology in pythons. These models are valuable for future experimental approaches aimed at dissecting the genetic regulation of this interesting and important biology in pythons and other reptiles.

## Supplementary Material

 FASTA nucleotide and protein sequences described in this paper are provided in the supplemnetal file.

## Figures and Tables

**Table 1:** Python orthologs of mammalian coat color genes and associated pairwise percent identity.

Gene	pyth2hum	pyth2mouse	pyth2dog	pyth2horse	pyth2cow	pyth2chick	pyth2anole	pyth2Tham_sirt
ASIP	46%	39	45	39	48	52	79	86
KIT	64	64	64	64	65	71	83	89
MGRN1	74	73	77	73	74	78	80	92
PMEL	40	48	39	41	39	50	66	77
OCA2	86	83	86	86	73	89	93	82
MYO5A	89	88	88	86	89	89	92	82
TRPM1	76	74	76	76	76	84	86	91
RAB27A	90	92	90	92	91	93	94	95
SLC24A5	76	71	75	76	75	77	85	0
EDN3	42	41	43	48	42	64	60	78
MITF	80	77	80	79	72	88	94	94
SLC2A9	68	67	68	71	68	73	83	88
RAB38	74	76	76	78	78	82	83	0
PAX3	98	96	97	97	99	97	99	99
ATRN	79	80	80	95	77	83	87	91
OSTM1	62	57	59	62	57	60	66	86
MCOLN3	82	80	82	78	81	85	90	95
KITLG	40	49	46	46	46	62	73	85
TYR	71	72	73	73	74	76	81	87
LYST	64	62	63	63	63	67	79	92
SOX10	82	83	83	79	82	95	97	99
STX17	74	71	74	74	74	76	81	87
EDNRB	64	61	62	61	60	80	79	64

pyth2hum: python and human; pyth2mouse: python and mouse; pyth2dog: python and dog; pyth2horse: python and horse; pyth2cow: python and cow; pyth2chick: python and chicken; pyth2anole: python and anole lizard; pyth2Tham_sirt: python and garter snake. *Identity: best blast hit between proteins*.

**Table 2:** Pigmentation gene expression network components organized by reference and component type.

Reference	Transcription factors	Promoter/target gene	Regulatory elements	Transcription factor binding sequences	Evolutionary conservation	Year
Bentley et al., 1994 [42]	USF, MITF, SP1	Tyrosinase, 115 bp fragment	M-box, CR1, CR2	CATGTG, GGTGGA, GTGATAAT	Turtle, quail, human	1994

Besch and Berking, 2014 [43]	POU domain TFs, POU3F2 (Brn2)	MITF promoter		ATGCAAAT		2014

Fuse et al., 1996 [44]	MITF	MITF promoter, melanocyte	GATA, CRE, TATA-like	CATGTG		1996

Gorkin et al., 2012 [45]	SOX10, MITF, TEAD1, JUND, FOS, JUN	6-mers and 7-mers are more informative in these analyses than k-mers of other lengths	2489 putative melanocyte enhancers	ACA[AGC]AG SOX10		2012
				CAC[AG][TG]G MITF		
				GGAAT[GT][TC] TEAD1		
				[AG]TGA[CG]TCA JUND,FOS,JUN		

Loftus et al., 2009 [46]	MITF, SOX10	Gpnmb	E-box motif	CACGTG, CACATGT, CACATGA (variant in other species), TCACATGA	Mouse, rat, dog, cat, horse, platypus, chicken	2009

Moro et al., 1999 [47]	MITF, AP-1, AP-2, SP-1	MC1R	E-box, TATA box, AP-1, AP-2, SP-1	GCCTGCGG AP-2		1999
				GCCCGGGG AP-2		
				TGACTCAG AP-1		
				CAAGTG (E-box)		
				CAGAGT (E-box)		
				CACCTG (E-box)		
				CAGGTG (E-box)		
				CAGGTG (E-box)		
				CAGCTG (E-box)		

Murisier et al., 2006 [48]	SOX10, SP-1	Tyrp1 melanocytes	E-box, SP-1 sites	AACAAA,		2006
				CANNTG E-box consensus		
				[AT][AT]CAA[TA] SP-1		

Murisier et al., 2007 [49]		Tyrosinase, retinal pigmented epithelium	DRE located at −15 kb acts as a strong transcriptional enhancer in melanocytes		Conserved noncoding sequences (CNS) that might represent putative novel regulatory elements of the Tyr gene	2007

Murisier et al., [50]	MITF, SOX10, Brn2, Mitf, Otx2, Pax2, Pax3, Pax6, Sox10, Tbx2, USF-1	Tyrosinase, core enhancer requires the E-boxes and Sox10 motifs	DRE: distal reg. Elem, E-box, M-box	CANNTG E-box		2007
				AGTCANNTGCT M-box		
				[AT] [AT]CAA[TA] potential SOX binding		

Schwahn et al., 2005 [51]	LEF-1	MITF, tyrosinase, DCT, TRP-1, PMEL17, Moel for DCT expression in proliferating and senescent normal human melanocytes	M-box that includes the MITF CATGTG, which overlaps with (ER-a), USF-1, TFE-3, Isl-1 and AP-1 binding elements	CTTTGGGTCATGTG LEF-1 & M-box		2005
				GGTCATGTGCT estrogen RE		

Vachtenheim and Borovanský, 2010 [11]	MITF, PAX3, LEF-1, SOX10, CREB, POU3F2, USF-1, p53, Tbx2	Mc1R, EDNRB, RAB27A, OA1, PMEL17, MLANA, Gpnmb, melanostatin I, Aim1TYR, TRP-1, TRP-2 (DCT),	M-box, E-box, MITF binding motifs	MITF binding sites: AGTCATGTGCT, ACATGTGA, AATCATGTGCT, GGTCATGTGCT, GCACATGAGT, GCTCACATGCT, TCACGTGTG, TCACATGAA, GGCACATGATG, ACAGCTGA, CCATATGA		2010

Vance and Goding, 2004 [52]	MITF, SOX10, LEF1, CREB, PAX3	MITF promoter	M-box (T) CATGTG (A)	–268 CATTGTC –262 (SOX10), -260 TTAATACTACTGGAACT –244 (PAX3), –228 AATTGGCCTTGATCTGAC –211 (SOX10A/B), –199 CTTTGAT –193 (LEF1), –147 TGACGTCA –140 (CREB), –127 AATTGATATCAACATT –112 (OC)		2004

Wan et al., 2011 [53]	MITF, SOX10, PAX3, STAT3, CREB, LEF-1, ITF2, FOXD3, BRN2 (POU3F2)	MITF promoter, tyrosinase promoter				2011

Watanabe et al., 2002 [54]	MITF, SOX10, PAX3	MITF M promoter	Distal enhancer	[AT][AT]CAA[AT]G SOX10		2002
				CATTGAA SOX10-s1		
				AACAAA SOX10-s2		
				TTTTGTT SOX10-s3		
				AACAAAA SOX10-s4		

Relevant information extracted from references is presented in the table. References are ordered alphabetically by the first author.

**Table 3:** InterPro domain annotation for pigmentation associated python transcription factors.

Gene	Protein name	StartPos	EndPos	DomainId	Domain name
CREB	cAMP response element-binding protein	87	146	IPR003102	Coactivator CBP, pKID
CREB	cAMP response element-binding protein	284	342	IPR004827	Basic-leucine zipper domain
FOXD3	Forkhead box D3	71	173	IPR011991	Winged helix-turn-helix DNA binding domain
LEF-1	Lymphoid enhancer-binding factor 1	1	65	IPR027397	Catenin binding domain
LEF-1	Lymphoid enhancer-binding factor 1	1	209	IPR013558	CTNNB1 binding, N-terminal
LEF-1	Lymphoid enhancer-binding factor 1	293	375	IPR009071	High mobility group box domain
MITF	Microphthalmia-associated transcription factor	303	370	IPR011598	Myc-type, basic helix-loop-helix (bHLH) domain
MITF	Microphthalmia-associated transcription factor	393	519	IPR021802	MiT/TFE transcription factors, C-terminal
MITF	Microphthalmia-associated transcription factor	4	142	IPR031867	MiT/TFE transcription factors, N-terminal
POU3F2	POU Class 3 Homeobox 2	51	125	IPR000327	POU-specific domain
POU3F2	POU Class 3 Homeobox 2	125	205	IPR009057	Homeodomain-like
USF-1	Upstream transcription factor 1	193	286	IPR011598	Myc-type, basic helix-loop-helix (bHLH) domain

Start and end positions are provided for each domain detected in the transcription factor protein sequences.
