# Supplementary material for: In Silico Analysis of Gene Expression Network Components Underlying Pigmentation Phenotypes in the Python Identified Evolutionarily Conserved Clusters of Transcription Factor Binding Sites
Source: Adv Bioinformatics. 2016 Sep 6;2016:1286510. doi: 10.1155/2016/1286510 (PMC5028829; doi:10.1155/2016/1286510)
Supplement: Supplementary file 1 — FASTA nucleotide and protein sequences described in this paper are provided in the supplemnetal file. [file 1286510.f1.pdf]

```
-- Input sequences --------------------------------------------------
>DCT 1500KB upstream region
TTACATGTAATCAGGAGGACAATCAAAAGCAAGGGAAGGGAAAATGAAAATCTCAAACAATTAATTAAAACAA
ACTAACATAACTGAAAAGAATACCCACATATGTACACTCTAACAGTAACAACTATAGTTATAGTACAGAACTT
TATAAACCTTCTAGAGAATTTTGAAGGTAAATCGGAAAAGAGGAGAACGTTCTTCTGATCATTTGCCCAAGGC
AATTGGATCAAAAGATTAAGAAGGCAAGAGAGCAATCACTGAAAATAAGTTTGGCCCAAATATGTAAACAAGC
ATGAGGTTTTAGAGAAGCTGGTCCTGGTTCCCGGTGCACTAATGAATTGGAAGACCTCTTAGGAACTTGGACC
ACGACTCATAGATGGTTCTGCTTCTAGAAGGCTTGAATCTTTATAATTACCAGTCTGTGAGCCATCTAACAGG
TGGTTGATCATCTTTGCTTTAAAAAGTATTTATTTTATAGTAGTAGGTATACAGTTACCATCTTAGATGCTAG
GCAAACACAGAATTGTATTAATAAACTGATGAGCCTGGTCAGTAAAATTTCTTATTTGCTCTCTCTAAACAAC
AGAAAACGTTCCTCCCAAATATTTAAAATTTGCTGAATGTGGATGCTCTTAGTCTTCCAGTGATAAGTACAAT
CTGCAAGCCTCTAAGTGAAGATCACTTTCTTTGTTGGGACATGACTGATGATGAACCGTTCCTCTTTACATCA
CTGTATGAGACACATCATAAAAATGGAGATTCATTTTTAAACACTGTAAGAGTCTTGGTATCATAGCTAGAAA
ACAGGTGGCAGAAAATGTCATTTGAATATAACCTGAATGTAGATATCAGCTCACTATGAAGGCTACAACATGG
TAATGTGTATTGTCTTCTGCCATGTGGTAGCTCGTGATCCTTTAAAGTTTTCAGAGTGAATTACAATATTCAA
CTATAGTTGCTGTGGAGGTCCATAATTTTGCAATCTCTTACAAAGCTCTCAAAGAAACTTGCACCCCTCAGCC
C
ACAGCATGAAGTCTGAGGTCGTGGTCTCATAAAGCACTGAGCAGAAGAAAAGATAAAGCAATGGGAATCAAAG
ACGTTTTGTCTCCCTCGTCCAGGGCTTCTCCCCCAGCATTCCATGGGTTTCTTTTTGCTTGTAATGTCATGTG
CTAATCATAAGGGTTTTCAAAAAGCTAATCCTTCTAATTAGGGTTTACTGACCCCCAAATATAAAATCTCCAG
CTACGGCCTCTTGGCAAACAACAAAGAGCTAAACTGTTTGCCTATTAACCACATTCAGTCCTGGTAATCTTGG
ATTAAGCAAAGACCCCTGAAGGTTAACCTAGTTGCAGTGAGGAGGAAAAATTGCTTCTCGGGTCAGTTCGAAT
GGGGCTTGCTCATCCAGCTGGAGATGGCTAATGATAATCTTTTCACTGTATCCTGCATTTAGAACCACAGGCT
GAGCAGCAGAGTTTTGGAAGCTCTGGGAATTAGATCTTG


>TYR 1500KB upstream region
ATTGACTTCACTGTGCATCATGTTTTCTGCTAGAAAATCCCAGGTTCCCCCCAAGTAAAATTGAATAGACTGG
AATAATGGCTTTCTATTGTATTTCCATCAGAACGTCCATGCTAAAAAATGAAACCAGTCTCTACAAGTTCCAT
ACTGTCCTGCCCAACTGCCTATTCTTGTTGAAGAGCCAGCCTTGGACATCCCAAACTTTCTGAATGGAGCTGC
TGACTAGCAGAAAAAAAAAGGGGGGGGGTCAAAGAAGCCTCTTGGTTTTGCTTTCTTCTTCACTCAATTGTTT
GTAATCTCAAATTCTTTCCATAGCATTTGTAAGAAACAATATATGCACATTTTGATACATATGGAACGCTATA
GAACTTTTTCTTATTTCCTAGTTTTCAGAATTCTGGTTTGCCTAGAGGTTTAAGATGGTCTGCATGGTCAAAC
TGATTTTGCTGCAGTTTTAATCTTCCAGTGTTTTGCTTGTTGATATACAGTATTTGTGTCCTGATTGCTGCAC
TGCATTTCCAACGTGCTGTAAGTCATCGAAAGCACTACGGCTGGAAGACAGACTGTCCCTTGAGATGTCAGTT
AAAAGCATTTCAAACAGCAGGGCTCATCAATATTTCTGAGCCCGAGACTTCAGGGAGCTGTTGCCGGTGAGAA
TACACGCATCACAGGACCAGCATAAGTCATCTTCATTTCATATGATATGATCATAGCAATATGCAAGGAAGCC
CAGATATTCATAGTTTTCTAGCTTAAAAGTAGGCTGAACCAACAGTTCTCTGCTACGTTTGAGTGCAAGAGAT
CCCAATGCATTTTGTAATATGGAACCTTACCACGCTGTAGGTGGCACTAAGACCTGAGCAGCTACGGTGCTGC
AAAAAGATAGAGTCATACAGCTGGAAGACCACCATATCCAAGTCCTGCTGAATGCAGGGATTTGTGTGAAAGC
AGCCCAGACAGATGGCTGTGGAGCCTTTGCTTAAGCATATCAAGTCAAGGGTAGTCCACTACCTCTCTGTTTA
G
TTGGTTCCACTGCTGGACTATTCCTACCACTAGATGTCTTTCCTAACATTCAGGTAGAATCTGCCTTCCTGTA
ATTTAAACCTGTGTTTTGCGCTTAGGAATAAGAGAGAACAGCTCTTCCATCTGACATCCATTCAGGTACTTGA
AGAATGCTTCACCTCAGTCATCTTTCCTCAAGACTAAGAGTACACAGCTCCTTCAACTTCTCTTCACAGAAAT
GCTTTCTAGTCCCCTGATCATCGCTGTTGTCCTTCTCTGAATCTTTCTTAAAGCTTGGAGCCCAGAATGGGAC
ACAGTAAGATGGGATTTAACCAAAGCATAGTTCTTATCAGAAACCATCTCCACATCCTTTACAGTCCTAGAAT
CTCTGAATCTTACTTCTAAAATTATATCAGGAATGCTTTGCATTAGTGCAGACCTGTAGATTTTCAATTGCTG
CATGTACCATACAAAATTCTTCCTCTCCCTCCTCCCCAC


>AIM1 1500KB upstream region
CAGGATTTTTGAAGATACATGCATAAGACTAAACATTTTTTCAAGAAAGAGAGAAGTGGAAGCAGACTAGAAA
AAAATGGAAATAATAGATGATAGAAAAGAAGGCAAAACCACAGGTTTGCCTGACAGACAAATCCTAGGACCTA
```

CAAAGACTGTGGCAGCAGCAAACATGCCCACTCCCTTTATTGGTTTCCTTCTTAATGGAATATTATTTTTATG
CTTTTTTTAAAATGGTAGGTTGGCTTGAGTGTCAAAAGAAAATAAATGTTAAATGTGTTTTTCTGAAACCTAC
ATGGAATTTCAGTTCAGATTCAACTACCACTCTGCTTAAGCATTACTCCCAAATCACAGCATATCAGGAAAAT
CCTCTGTCAACTGCAAAGACAGAAGATGGCCCTGTGTATTATTTTTCATCAAACATCAATATAATTTTAAATT
TTTGCATTTTTGGAGCCAATGGGGAATTTTTTTCTATCATAAATTGAAAAATGGAGGGGTAGCTTCTATCAGG
GTTATAATCTGGGAAAGAAGGATTACTTTTAAGAAGTAATTTAAAAAATTGTACCATACTTACAATTCATGAG
TGGAATAGTTACCCCAAGATTCACTATGCTTCACTCCAATGTGCTTTTGCTATTCCTTTACTCTTTTCCTGTG
TATACATTTTTCAGATATGCAAAGTTTAGAATTACGTTTACAGTACAAGCTGCTACATGCCTGTATAGTCAGA
AATAAATCGCTGAGAGTTCAGTGGTGAGAAGAGTATTGCCACATGAATGGTGACCGGGTGGGCCTGTTTTGCT
TCTGAGTCCAAACTATTGGCCCCATGGACTCTGTTGTTAAATACAAACATATCAGGCTTTTTGCAAAAATAAA
AATCCCTTTAAAAAATCTCCTTTGCTTCAAAGAACTCACAGCAGAGAAGACAGTATGTCCTGGACTACCTTAA
ATGTAGCAATTTGTCTTTTTCATATGGTTTCTTATTTATTTATATATCAAATTTATATAGCCGCCCAGTTCAC
T
CTGGGCAGGGTACAACGAAGATATAAAACAAGAGTTTCCTGGATACTTGCAGCCCTTCCTTGCACAAACTGTT
GATCATTTCGTCTCCATCAGAAAGAAACAACAACCCTTATTACTGCCTTGCAATTTATATCCTCGGATCAGTC
GAGTCTCTCCCTTCTCGGGTAACACGCAGTATGAAGGGGAGCTGGAGTGGCAGTCAATTTTCTGCTTCAAGAC
CACGAACTGGCAGCCCTTTAAACGGAACAAAGGAGGGTCCCGAGCCCCGAGAGAAGTAGTTGCAGTCGCGGAA
ATAGGAGCGAGAGATAAAACTTAATAAAGGCACGAAGAGGAGACAGGCGAATTCGTGGCCGTCGCTTGAAAGT
TTGTACAAGCCAAGCTGAGGAGGTGGGAAGGGTGCGGTCCGCACCGCGGTTGGAGCAGCTGACAGGGCCGGCG
GTGCTCCCGCAGCCTTTAGGAGGCGCTTTGGTTCTCCCC

>MLANA 1500KB upstream region
ACTTCTCTTTTGCTGAAAGTAAGTGACCCAATTTAGATTGGTTAAGAGGGGCATAATGGAGAGATTTAATCCA
GTGGATTGTTATGCCCCCAATTCAAATCCTCTGTGGTAATTTTAAAATAATGAAAAGGGCCATGAATTTCCTA
TAGTATAACCAGTTAGATCTTTAATCAAATAGTTGTTCATCAAGTAGTTTAATTACGTTCAGGGGACTTACCC
CTAAATGTATATCAGACTGCATCTTGAGTTCCTGAATTTGAGTTGAAGTTACCAGATTTAGATGCAAAGCTAA
ATGTATTTTTAATATAAGTATTTATTAAATTTATATACTGCTTCTTTTTGAAGAGGCCCTAAGTGGTGTATAT
AATAAGTGACGGTAATATGACACCAATGCAAAATACCATGGAATACAGAACAAAAAAACACCATCGGCATACA
AAGTTCATTTATAGATATGAAAATGCTGAGAAACGTGGAGGTTGAGAGGGATTGAATGTTGTCATCTCACCCA
TAAACAGTCACTGGGAATTCCTACCAGTACCCTCACCTACTACTCTCGCATAAGGGCATGAATTGGTTGGAAT
CTAATTGCTCAAAGACAAACAGAAAGTGATAATACCAGACCTTCAATAAATCCTATCACAACAAAACCTGAAA
AATCTAGAAGATGAAGAACACATTTTTAGCATCTTCTCCAAAGTCCTTTCAACAAAAAAATGTAAATGATTGT
TTCAGGAGACTGTAATGCCTGATTAATTGTGCTATTTCTTTCATATTAATACTTGAATTGTTTTTAATGTGCT
TGTTTTCTGTTTCATTTGAGGAATATATCAACATTGTTTCGTTATGAATTATTCTGAATAGAGTTTTAAAAGA
GCAGGATGTTGATAGTAAATTACTTAATTTCCTCACCAAAGCTTTTCTCCCAGCTATTCTTTCTCCCAGTATT
ACTCCAAAGGAAAAAGGCAAGTAGGAAGACGTATTCTGGGGGCAAAATGTAAGAATCATTAGAAGTTTTTTTG
C
ACCCTAAATGTGAGTGCATGCACAGTTTATACCAGATTGGACAGGACTGGAGATGGGTCATAATCCTGGATCT
TATTTTGCTTTTAGAAAACTAGCAAAATAAGCTATTGAGTTGTATAAGTAGGCACTATCTGATGACAGGTTTT
GAATACAGAGAAAGTTCTATTGCATCTTATAATGTCTAAAAGATAAAATTGCCTCAGATTGATCTTAAATCCT
GGTAATTTATATAGACACATCCTTGCAGTTTTCTTGCCATAATACAGAAGTGATTTTTCATTGTTTTCTCCCA
GGAATTTTTTATTCATTTTCCCAGTTTAGGTCATAGCTCTGGATTTCCCAGATGTTCCCCACAATCCTGATGA
AATTTCTAGATCAGCCAAGGTTAGCTAAATCCCCTTTTTGTTAGTATTGCCATGGATTTCAGGAAGACTGGTC
CTTGTTTTTTATAATATTTATTACATGTATAGTTTTAAA

>MC1R 1500KB upstream region
TATCCAAATATAAATTGCTTCTCAGCCTTTTGGCTAAGATCAAGGGTGCACGGTAGACGTAGGCCGTGACTGA
TCCACCTGGCTCACCTTGATGGCAACGGTCAGGTGCAGCGACCACCTTCCTTGGTTTCCTTCTCCATCATAAG
TGATCACTCCGGGGTTCCAACAAAGGTGGCAGTTTGGCTGCTTTGCCCTGGGTGCCTCCACATCAGTTAGCAT
GATCTAGAGATTACGGGGCATTTGTACATCTGGACAGGCAGAAAAATTATCCCTTGATATGCCCCTACAGATC
CAATCTCAGAAGAATTTGACTACTAATCGAATAACATGCCATAATAATAAAGAAACAGACTCAGAAGTAGATG
ATGATTCTTTCAAATTCCTCATGGCAGAGACCTTTTACCACCACCCAGTTAAAAAAAAAGGGGGGGGGTCAGA

AAACTGGCCGTGCTGAAGGCTAAAAGAAGGCATCCTATCAATATATGGGGTGTGTGTAGATAACTCAAGAAGT
GATTCAGTGGCAAATGCTGAACCAGCTAATACAGGTCTCCTGTAGATAGGCCCAGTTTGTTACTTAAGGCTAC
TCTGAACATAACAGGAACCATGCTGAAAGAAATATAGGAAAGGGTACTTAAAAGAACGGCATATCAGATATGG
TTGACGCTGTCCTTGCGACAACCTGGCTCAGCAGCAGCGTGGCCCAGCACTCTCTCTGCTGCCACCCGTGGCC
CACATTCCACCACTCCACCTTGGGAGCCCAGGTGATGATAATCCATTCCTGTTTCTGGTTTTGTAGCACTTCA
TCTTCGGGAAAGGCCATCCCACTGTCAGTGTATTTACAGAGGCAAAACGCATCCTGACTTTAAGGACTGTTTC
ATAACATCTCCCTGCCTTTCAAAGTGCTCGCCGCCTGACTTATCATCATGTGCATCACCCTCTGCCCCCCTGC
AAAAGGAGGAGCTGCATCTATCCCTTTAAAAAAGGGAAAAGAAAGAGTTCTTTCTTTGCTGTAAATTTTTGCT
A
ATCTATTTTGGAGGCAGAGACAAATTCAGCAATTATAACATTAGGAAGGAAGCCAAGCAGAACTTTGACATGG
ACACACCACTTCCCAAGGAAGATGCTGGGGGATCGCTCTCCTTAGCCGCATAGGCTGAAGAACAAAGGAGACC
TGCAGAAACCAATGACAGGGGAAGAAAGGCCAAGAAGCCACCACTTTAGGATCCTTGGAACGGTATTGCGACC
AAAGAAGTCCTTGTGGAAGCTTGTGTCTGCGCTGCTCGCTTTGCTCTCCCGGACCCTTTGTTGGGGAGCATGT
GATCTCACCATCTGGGATTTTCTGGGAGGAAAAGTGCTGACAGAGGAACCTGTAGCCTACAGGTGAGTGTTTG
GGACAGGCTAAAAATACCTGCATTCATCTAAAATCACTTCAGAAGAAACGCTCTTCCAAAATCTGTGTCCCCC
CCTCTTATTTCTCACTAGCAGCGCCACAAAGTGTTTGAG

>RAB27A 1500KB upstream region
CATTTTCTTAGAACATTATCCACGTGCATAAGAATGCGGTCACATTTTAAAGTGCCTTTTGCCGAACTTAAGT
GCTGCCAATTAATAGGCCCTGTGAACCTCGTTTCTGCTTATGTCCTCAATCAAAAGGAGCAGACCCCCCTCCTT
TAGGCACTCTCCCCACTTCTGCCAAGGGCCTCTCATTTCCATGGCACCAAACTCAGTCACGCTGCGCTGTGGA
CCAGCCTTCCCTATGGTGCTCCGTGTTGGCCCAGGTTCAAGATGCTCTTAAATAACGCGCTAAAAGGATTTTT
TTTCTCATGCACTCTGTTCCACCATAATAGCTGAAACAAGTCTTTATTAAGGAGTCATATGATGCTGATTATA
TCTTGATTTGTCAGTTGTACAGCAAAACCACAGATCTTCCCTGTTTGTGGCAGGAAAACCACAAACCGTGGTG
CCTGTTGGAGTTCTTCCTGAATTCAGTTCCTCTTTAAACACTACACATGCAAGGAGATGTGGAGAATTTGGCA
GGAAAAGAAGTGGTCCAAGGCCAATGGAAAAACATCAAGTATATGGAGGAAGTTTAATTAAACGTAGTATAAG
GAAGAACTGCTTTATTAAACTTGCCATGCACACAAGAAAATTCCTATGAATATTACCACATGGGGGGTCTAAC
CGAATGCCCTTTGGAAGGGCGGGGGGCTGCAGGTGTCTGTCTGCCTCCCCACATGCCCTCAGCCCTGCGAGGC
AACACCCAAGGGTTTGGTGTCTGCAGGGGCACGGACCTGTGCAGACCAGCCAGGCGTGCAGCAGAGGCAGCTC
GCGTGCACTCCTTGCAGAACTGCAGTTGCAGTCAGTGTCTGCGCTCTGGAGAACAGCTGTGGACCAGGATCTC
CTGGTTTCCGAACAGAGCAGCCCTATCACTTTACCAGCTGAAACAGACAAGAGGGTCTTGCTCCAAGGCAGCG
AGTTGCAGGTTGGTGCCTGCCTTCTGGCATCCTCGGTCTGGATCCAGACAGGAGCCTTCGAGGTTCAGGATCC
T
TTTGAAAAGGATGTGAATTCTTTGCACTTTGGGGGGCTTCACACATACTCCACTCTGATGACGTATGAAAGAA
AACACCCAAGGTGTGCAATCAAACTGATTTAGCAAGAGAAATGGCTGTTTTAGCTGCAGGGACTGATAAACCC
TGGGCAGCACCACTTCCTACAGAAGCCGGCCATACCGTGACGGTTTTCCTCGCTAATCTTTGCAGCAACAAAG
CAAGCAACAGCAGCTCCTGATTCATCCTGCACTGTTGAGTGAATGGGCACGTTTTATCCTTGAAGTTGCCAGG
CTACAGAAGCCTTCAGCCGGGGACAAGGCTGCTTCCAAGGACTGCCAGAGAGTTCCCTTCCCCTCTTCCACGG
TACCTCCCTGCAGCAGTGGCGGCATTCAGCCTCAGGGCCTCCCCTGGTTCCTCTGCCCAGTCCCCCCCCCGCC
TCCTCCCCAACACAGGAAATCCTGCTTCCAACTGTGGGG

>MITF 1500bp upstream region
AAATGTATGTTCATCTCAAGAAAGTCTTATATAGGTATAAGATTTATTTGGACACTGATCCCTGTTTAGATCT
CTGTCACAACAGTGCTGTTTGCTTGCATTATTATTTCAGTGGAATTTCTGGAAGAAAGAACAATAAACATTAA
TAAAATAATTCCCATTTCTAATTCCCATAATGCTCTTAAAGTAACTAGATAGCTATGCTGTCCTATGGTATCC
TATGCTGACCTGTCCTACCTTATCCTACCCTACCCTACCCTACCCTACCCTACCCTACCCTACCCTATCCTGG
CTTGTCCTGTCCTGTCCTATGTGTGTCTTTAGCCTAATCTACACCTTTGAAAACTCTCTTATTTAAAATTTCT
CTCTTAAGCACTAATACATCTAATAAATTATTTTCAGATACTACATAGAAGAGTCACCTTCACTGATTACATT
TTTTTTCAATATTAAATTGTTTCCATTTTACATATTTTGATACAGTTCGCACTGTCACCCAGTAGCACCTGAC
GTGCCCATTTCACAAAGGACTTTTAGATGCTTGAGAATAAAATGCTTCTTGTTTGCCTCGACAAAACTTTGGC
CCTATTGTTGTAATGCATGCAGGGTCTAGAACAAAATGCTGCAGGAGATTGTCCAGACAAAGCTGCACTACCT
AGACATTCACCAGTTTTCTCACAATGTTGTCAGACAGTAATTCCCCTGAATATAACATTCCTGTCTTGCTATA

GCAGTTCTTTACCTGAGCTGCTGGGTGACAGCCCCATTGAGCTGGAGTAGAAAATTCCCATAGAGCTGGACTA
GGAAGTTCCCATTGAACTGGATTAGAAAGTTCCCACTGAGCTCTACTAGAAAGTTCCCATTGAGTTGGACTAG
AAGTTCCCACTGAGCTGGACTAGACTGCTGAGTTCAGGTCCTCCCTTAGGCATGAAAGCCGCCTGGGTGACTT
TGAGCCAGCTACTGTCTCTCAGCACTACTTTATAGGGTTGTTGCTGGGAAAAAAGTAGGCAGGAGCATTATGT
A
CACTGCCTTGAGTTGCAAAAATAAAGACAGGATATCAGTCTAATAATCAATAAATCTATAGGGACCTCTTATT
GAGAGGGCATATATTTGATTCCATTTTCAGTATGATAACTGGGCCCCTATTGGTTCCAAAATCAGGCCTTACC
AAAAAAAGAAAAGAAAAAGGGAATAAAGGAATCCTGACTGAAAGAGTAGGGAAATCAAAGACAACTTCTGTCT
CCTTTTGAATTAGTATCGTATAATCACTGGGGTGCTGATCATTAGAAAGACTAGAACATAGCTAACTTCTTGT
GTTAAACAGAGATCAAGCCCAGAAATCCATATACATCCACACCCCTGCCAGGCATTTTTCTACACTTGCAAAG
GTTTTTGCCCACTTCCTAGCACATGCAATGGCCCCCTCCCAAAGCCTGACTCTTTAAACTCCAGCTAATATTT
ACTGAGCAGTGATGAGTGTTCTCTGGTGTTTCCCTCAGC

>OA1 1500KB upstream region
TGTAGACTTCAACTCTCNNNNNNNNNNNNNNNNNNNNNNNNNNNNNNNNNNNNNNNNNNNNNNNNNNNNNNNN
NNNNNNNNNNNNNNNNNNNNNNNNNNNNNNNNNNNNNNNNNNNNNNNNNNNNNNNGAGTCTAGTGCAGTGTTT
TAAAACCTAAGCAATTTAAAATTGTGTAGCCTTCAACTCTCAGAATTCGGGGGGTTGAAGTCCACACATCTTA
AAGTTGCCAAGTTTTAAAAACACTGGTCAGGTGTAAAAAAGGGTTACTGAACCTGGGCTGCAAAAGTCCAGCA
GACACCACTAGACAGCTGCAGCAATACAGAAAACTCTATATCACTGCCATCATCTAGCAGTCATCTGGATTTC
TGAGCCCAGATGTGGTATGGTAGTCCAAAGGTAATTCAAGATGCACTGGATCCATGCCTTACTTTCTCTTCCT
TCTACTTCATCATCTTCTCAGAGACGCAATAACATCTTTGAGCAGTAATTAGTGGGCATGGGGGTCCGATGAT
CTCACCGCTCAGGCCTCTGAAGTTTTTCAGGAGTGCCGGCACAGCTACCCGCCGGCGTGGGAAAAGCTGTTTT
AGGGCAGTCAGGAGTTCAGGAAACAAATCTCAGAGCGGTAACAGAACCACGTGCAGGGAGGAGGATTTATGCC
CCAGCAAGTCATGCTAAGAAAACACATAGGCACAATTGATGAGAAACGGTAATCCTCTGTCCCCAATTTACAA
AACGCGGAGGTTGACCCTATGCAGGGAGTCCTTTTCAGACTGCGCCTTTTCAGAACTCGGAAGAGCTGCTCCT
TTTCTCTGGCGCGCCGCGCCCCAAGAGGGCTCCGATCCACCCGCTCCAGCGAGAAGCCAATTCGGTTTGAGTG
AAGCCCTGCCAACGCCTCGAAGCAAGGGGAATGAAAGACACGCGGTTCGCCCCCCCGCTCTCCCCCCGCGTCT
CGCTGCCCTAGTACGATTTTCCCCCGGCGGGGAGGAAAAAAAAAAAAGGANNNNNNNNNNNNNNNNNNNNNNN
N
NNNNNNNNNNNNNNNNNNNNNNNNNNNNNNNNNNNNNNNNNNNNNNNNNNNNNNNNNNNNNNNNNNNNNNNNN
NNNNNNNNNNNNNNNNNNNNNNNNNNNNNNNNNNNNNNNNNNNNNNNNNNNNNNNNNNNNNNNNNNNNNNNNN
NNNNNNNNNNNNNNNNNNNNNNNNNNNNNNNNNNNNNNNNNNNNNNNNNNNNNNNNNNNNNNNNNNNNNNNNN
NNNNNNNNNNNNNNNNNNNNNNNNNNNNNNNNNNNNNNNNNNNNNNNNNNNNNNNNNNNNNNNNNNNNNNNNN
NNAAACGGAGCCGGGAACGCCAGTCGGGGGCTTTAGCCGCCACTTCGCCACATGATCCGGCCGCCGCGTGACC
CCCATCTCTAAGCCGAAGGAGGAATTCTGTACAAAGGAAAGGCTGCTCGGAGCGGGAGAAAGTGCGCGGCCCT
GCGTCCCACCCCGACCTGCCAGTCTCGCCCAAACTTTCC

>PMEL 1500KB upstream region
TTTTGTGGATAATGATACAAACGTAATAAAGAAGAAAACCCTCATCAGGATGAGTAGCCTTCTTGCAGAACTA
TGCCAGCTTTTGTTTTCCCCCAAAGTTTTGTTATAAGGCGATACAGGAAGTCTTCATATTGCTGTGGCAAAGT
GGTTGATCTGTTTTTGGTGGTGGTTTTCCCAAATGGCTTGTAGCTGCTCATACAGATATCCATCTGTACTTAG
AAAAATCAAGACAGCTAAACTAGTCTTAGATTGGAAGTCAAGCATATCAGTTTAGTTGAAAAGCCCCCTTTAT
ACCCTGCAGTTTCAGAATTTGTAAAGAGGTCCATCCCTATACAACAGTGTTTCTCAACCTTGGCAACTTTAAG
ATGCGTGGACTTCAACTCCCAGAATTCCCCAGCCAGCATGGCTGGCTGNNNNNNNNNNNNNNNNNNNNNNNNN
NNNNNNNNNNNNNNNNNNNNNNNNNNNNNNNNNNNNNNNNNNNNNNNNNNNNNNNNNNNNNNNNNNNNNNNNN
NNNNNNNNNNNNNNNNNNNNNNNNNNNNNNNNNNNNNNNNNNNNNNNNNNNNNNNNNNNNNNNNNNNNNNNNN
NNNNNNNNNNNNNNNNNNNNNNNNNNNNNNNNNNNNNNNNNNNNNNNNNNNNNNNNNNNNNNNNNNNNNNNNN
NNNNNNNNNNNNNNNNNNNNNNNNCAGCCAGCCATGCTGGCTGGGGGATTCTTGGAGTTGAAGGCCACATCTT
AAAGTTGCCAAGGTTGAGAAACACAGCTATATAATATTTTGTCTTCACCTACTGAATTACTCTGTGTCAAATT
ATTTTTCTGTATTGGAGTGGGAGTGGTTGTGTGCTTAAGTGACTTTCATTCTAACAGATGGCTCACCCATCCC
TCTGCATTCCATGACTTTGTCCAGAAATGTAGGACCACAGCAAACCCTGCCTCGCCTGCTGTCCAAGAAACCC

TAGACCATGCCCTTGCCTGAGAATTATTTTTGATTTAAATTGTGGATTCCTTTGCCTGCTTTGACTTGCTTCA
T
ATCACTATCTGCCCAGGCATCCAATTTCAAAGGAGATTTTGAAGTTACAAGAATATGAAATGGTTGCAGATAT
TCATGCTCACTTTATATCAGCCTGTCCTGTTTCTCCTGTTCTCAGTGCTAGCACCTCTCTTTGGAAGAAGTTT
TTCCAGAATTTGAGATGCCACCCTCGTTCCCCACTTCTAGCTCTAGAATTGCCCAGGCATGCTTCTGTTTCTT
CTCTCTCATCTCTTTCTGTAGTGTTCCCTGGAGCCACCAGTACCCCAGTTTGAGCTCTGGTATATTGCTCATG
TGCAGTGTCCATCAACGGGGCAGCTGGAAATCTATTCCCTTATGAGGTGGCCTATTGGCGCTGACCAGGTCAG
GCAAATTCTGGACTACCATTCAGCTAGCAACTGGGGAATCGCCATTTGACTGCTCTTCCACACATAAATGGCT
CACATGGTGTAGACAATGATCACACTGAGAAGTAGTCCA

-- Factors predicted by PROMO in these sequences ----------------------
NAME; MATRIX_WIDTH;
POU3F2 [T00630]; 7
USF-1 [T00875]; 7
Mitf [T01554]; 13
USF1 [T00874]; 9
LEF-1 [T02905]; 8
FOXD3 [T02290]; 8
FOXD3 [T04166]; 11
USF-1 [T00877]; 11
CREB [T00989]; 10
CREB [T00163]; 9
CREB [T00164]; 9

-- PROMO predictions detail --

Sequence name; Factor name; Start position; End position; Dissimilarity; String; RE equally; RE query
DCT 1500KB upstream region; POU3F2 [T00630]; 41; 47; 0.000000; AAATGAA; 0.18311; 0.21080;
DCT 1500KB upstream region; POU3F2 [T00630]; 47; 53; 8.841973; AAATCTC; 0.27466; 0.33169;
DCT 1500KB upstream region; POU3F2 [T00630]; 160; 166; 5.963103; AGAATTT; 0.73242; 0.98415;
DCT 1500KB upstream region; POU3F2 [T00630]; 174; 180; 8.361098; AAATCGG; 0.64087; 0.67044;
DCT 1500KB upstream region; POU3F2 [T00630]; 203; 209; 5.457738; ATCATTT; 0.73242; 0.73821;
DCT 1500KB upstream region; POU3F2 [T00630]; 261; 267; 5.963103; AAATAAG; 0.73242; 0.98415;
DCT 1500KB upstream region; POU3F2 [T00630]; 276; 282; 7.730221; AAATATG; 0.91553; 1.19566;
DCT 1500KB upstream region; POU3F2 [T00630]; 462; 468; 5.963103; AGTATTT; 0.73242; 0.98415;
DCT 1500KB upstream region; POU3F2 [T00630]; 466; 472; 0.505365; TTTATTT; 0.54932; 0.77377;
DCT 1500KB upstream region; POU3F2 [T00630]; 554; 560; 7.730221; AAAATTT; 0.91553; 1.19566;
DCT 1500KB upstream region; POU3F2 [T00630]; 555; 561; 9.094656; AAATTTC; 0.27466; 0.33169;
DCT 1500KB upstream region; POU3F2 [T00630]; 561; 567; 5.963103; CTTATTT; 0.73242; 0.98415;
DCT 1500KB upstream region; POU3F2 [T00630]; 600; 606; 7.730221;

AAATATT; 0.91553; 1.19566;
DCT 1500KB upstream region; POU3F2 [T00630]; 601; 607; 7.730221;
AATATTT; 0.91553; 1.19566;
DCT 1500KB upstream region; POU3F2 [T00630]; 608; 614; 7.730221;
AAAATTT; 0.91553; 1.19566;
DCT 1500KB upstream region; POU3F2 [T00630]; 609; 615; 7.730221;
AAATTTG; 0.91553; 1.19566;
DCT 1500KB upstream region; POU3F2 [T00630]; 750; 756; 2.650678;
AAATGGA; 0.27466; 0.41195;
DCT 1500KB upstream region; POU3F2 [T00630]; 759; 765; 0.000000;
TTCATTT; 0.18311; 0.21080;
DCT 1500KB upstream region; POU3F2 [T00630]; 815; 821; 8.589292;
AAATGTC; 0.64087; 0.67044;
DCT 1500KB upstream region; POU3F2 [T00630]; 819; 825; 6.822173;
GTCATTT; 0.54932; 0.52790;
DCT 1500KB upstream region; POU3F2 [T00630]; 970; 976; 5.963103;
ATAATTT; 0.73242; 0.98415;
DCT 1500KB upstream region; POU3F2 [T00630]; 1225; 1231; 2.272484;
AAATATA; 0.27466; 0.41195;
DCT 1500KB upstream region; POU3F2 [T00630]; 1232; 1238; 8.841973;
AAATCTC; 0.27466; 0.33169;
DCT 1500KB upstream region; POU3F2 [T00630]; 1362; 1368; 9.978215;
AAATTGC; 0.18311; 0.18057;
DCT 1500KB upstream region; POU3F2 [T00630]; 1441; 1447; 0.000000;
TGCATTT; 0.18311; 0.21080;
DCT 1500KB upstream region; USF-1 [T00875]; 96; 102; 5.121209; CCACATA;
0.73242; 0.41833;
DCT 1500KB upstream region; USF-1 [T00875]; 363; 369; 11.575332;
CCACGAC; 1.09863; 0.59829;
DCT 1500KB upstream region; USF-1 [T00875]; 434; 440; 0.031764; CAGGTGG;
0.36621; 0.17808;
DCT 1500KB upstream region; USF-1 [T00875]; 619; 625; 5.974743; AATGTGG;
0.36621; 0.23847;
DCT 1500KB upstream region; USF-1 [T00875]; 804; 810; 0.031764; CAGGTGG;
0.36621; 0.17808;
DCT 1500KB upstream region; USF-1 [T00875]; 896; 902; 0.000000; CATGTGG;
0.36621; 0.17808;
DCT 1500KB upstream region; USF-1 [T00875]; 957; 963; 9.731297; GCTGTGG;
0.36621; 0.15583;
DCT 1500KB upstream region; USF-1 [T00875]; 1021; 1027; 9.731297;
CCACAGC; 0.36621; 0.15583;
DCT 1500KB upstream region; USF-1 [T00875]; 1040; 1046; 11.575332;
GTCGTGG; 1.09863; 0.59829;
DCT 1500KB upstream region; USF-1 [T00875]; 1290; 1296; 5.974743;
CCACATT; 0.36621; 0.23847;
DCT 1500KB upstream region; USF-1 [T00875]; 1452; 1458; 4.610088;
CCACAGG; 0.36621; 0.13317;
DCT 1500KB upstream region; Mitf [T01554]; 692; 704; 13.639481;
GGGACATGACTGA; 0.00563; 0.00244;
DCT 1500KB upstream region; Mitf [T01554]; 891; 903; 14.563599;
TCTGCCATGTGGT; 0.02535; 0.01042;
DCT 1500KB upstream region; Mitf [T01554]; 1158; 1170; 2.772353;
AATGTCATGTGCT; 0.00282; 0.00123;
DCT 1500KB upstream region; USF1 [T00874]; 904; 912; 8.481293;

AGCTCGTGA; 0.22316; 0.08113;
DCT 1500KB upstream region; LEF-1 [T02905]; 449; 456; 1.835267; CTTTGCTT; 0.13733; 0.10092;
DCT 1500KB upstream region; LEF-1 [T02905]; 685; 692; 0.904249; CTTTGTTG; 0.06866; 0.06728;
DCT 1500KB upstream region; LEF-1 [T02905]; 986; 993; 7.080257; TTACAAAG; 0.09155; 0.08101;
DCT 1500KB upstream region; LEF-1 [T02905]; 995; 1002; 7.737185; TCTCAAAG; 0.16022; 0.10553;
DCT 1500KB upstream region; LEF-1 [T02905]; 1088; 1095; 0.531842; AATCAAAG; 0.09155; 0.08561;
DCT 1500KB upstream region; LEF-1 [T02905]; 1260; 1267; 0.904249; CAACAAAG; 0.06866; 0.06728;
DCT 1500KB upstream region; LEF-1 [T02905]; 1318; 1325; 1.835267; AAGCAAAG; 0.13733; 0.10092;
DCT 1500KB upstream region; FOXD3 [T02290]; 54; 61; 0.461834; AAACAATT; 0.09155; 0.11425;
DCT 1500KB upstream region; FOXD3 [T02290]; 67; 74; 0.833571; AAACAAAC; 0.09155; 0.11446;
DCT 1500KB upstream region; FOXD3 [T02290]; 284; 291; 1.875536; AAACAAGC; 0.22888; 0.22054;
DCT 1500KB upstream region; FOXD3 [T02290]; 513; 520; 4.743818; AAACACAG; 0.13733; 0.12406;
DCT 1500KB upstream region; FOXD3 [T02290]; 577; 584; 1.851876; AAACAACA; 0.22888; 0.22054;
DCT 1500KB upstream region; FOXD3 [T02290]; 768; 775; 3.910247; AAACACTG; 0.11444; 0.10419;
DCT 1500KB upstream region; FOXD3 [T02290]; 801; 808; 5.320522; AAACAGGT; 0.13733; 0.10109;
DCT 1500KB upstream region; FOXD3 [T02290]; 1257; 1264; 1.851876; AAACAACA; 0.22888; 0.22054;
DCT 1500KB upstream region; FOXD3 [T02290]; 1273; 1280; 3.444986; AACTGTTT; 0.09155; 0.08583;
DCT 1500KB upstream region; FOXD3 [T04166]; 114; 124; 8.459600; ACAGTAACAAC; 0.00429; 0.00335;
DCT 1500KB upstream region; FOXD3 [T04166]; 1256; 1266; 14.158476; CAAACAACAAA; 0.09012; 0.11075;
DCT 1500KB upstream region; USF-1 [T00877]; 895; 905; 2.080477; CCATGTGGTAG; 0.02003; 0.01131;
DCT 1500KB upstream region; USF-1 [T00877]; 1162; 1172; 0.693492; TCATGTGCTAA; 0.01144; 0.00719;
TYR 1500KB upstream region; POU3F2 [T00630]; 34; 40; 7.074855; AAATCCC; 0.54932; 0.52790;
TYR 1500KB upstream region; POU3F2 [T00630]; 57; 63; 3.156043; AAATTGA; 0.27466; 0.33133;
TYR 1500KB upstream region; POU3F2 [T00630]; 89; 95; 0.505365; TGTATTT; 0.54932; 0.77377;
TYR 1500KB upstream region; POU3F2 [T00630]; 118; 124; 0.000000; AAATGAA; 0.18311; 0.21080;
TYR 1500KB upstream region; POU3F2 [T00630]; 300; 306; 5.963103; AAATTCT; 0.73242; 0.98415;
TYR 1500KB upstream region; POU3F2 [T00630]; 313; 319; 5.457738; AGCATTT; 0.73242; 0.73821;
TYR 1500KB upstream region; POU3F2 [T00630]; 337; 343; 7.224857;

CACATTT; 0.54932; 0.52790;
TYR 1500KB upstream region; POU3F2 [T00630]; 374; 380; 5.963103;
CTTATTT; 0.73242; 0.98415;
TYR 1500KB upstream region; POU3F2 [T00630]; 437; 443; 5.710420;
CTGATTT; 0.73242; 0.73821;
TYR 1500KB upstream region; POU3F2 [T00630]; 486; 492; 5.963103;
AGTATTT; 0.73242; 0.98415;
TYR 1500KB upstream region; POU3F2 [T00630]; 511; 517; 0.000000;
TGCATTT; 0.18311; 0.21080;
TYR 1500KB upstream region; POU3F2 [T00630]; 587; 593; 5.457738;
AGCATTT; 0.73242; 0.73821;
TYR 1500KB upstream region; POU3F2 [T00630]; 612; 618; 7.730221;
AATATTT; 0.91553; 1.19566;
TYR 1500KB upstream region; POU3F2 [T00630]; 688; 694; 0.000000;
TTCATTT; 0.18311; 0.21080;
TYR 1500KB upstream region; POU3F2 [T00630]; 808; 814; 0.000000;
TGCATTT; 0.18311; 0.21080;
TYR 1500KB upstream region; POU3F2 [T00630]; 932; 938; 7.074855;
GGGATTT; 0.54932; 0.52790;
TYR 1500KB upstream region; POU3F2 [T00630]; 1093; 1099; 7.327537;
GTAATTT; 0.91553; 1.19566;
TYR 1500KB upstream region; POU3F2 [T00630]; 1238; 1244; 5.457738;
AAATGCT; 0.73242; 0.73821;
TYR 1500KB upstream region; POU3F2 [T00630]; 1325; 1331; 7.074855;
GGGATTT; 0.54932; 0.52790;
TYR 1500KB upstream region; POU3F2 [T00630]; 1406; 1412; 5.963103;
AAATTAT; 0.73242; 0.98415;
TYR 1500KB upstream region; POU3F2 [T00630]; 1444; 1450; 2.019801;
TAGATTT; 0.18311; 0.24110;
TYR 1500KB upstream region; POU3F2 [T00630]; 1474; 1480; 5.963103;
AAATTCT; 0.73242; 0.98415;
TYR 1500KB upstream region; USF-1 [T00875]; 832; 838; 11.506849;
CCACGCT; 1.09863; 0.59829;
TYR 1500KB upstream region; USF-1 [T00875]; 840; 846; 5.152973; TAGGTGG;
0.73242; 0.41833;
TYR 1500KB upstream region; USF-1 [T00875]; 904; 910; 12.460630;
CCACCAT; 0.36621; 0.23857;
TYR 1500KB upstream region; USF-1 [T00875]; 963; 969; 9.731297; GCTGTGG;
0.36621; 0.15583;
TYR 1500KB upstream region; USF-1 [T00875]; 1004; 1010; 11.607096;
CCACTAC; 1.09863; 0.59829;
TYR 1500KB upstream region; USF-1 [T00875]; 1029; 1035; 9.763061;
CCACTGC; 0.36621; 0.15632;
TYR 1500KB upstream region; USF-1 [T00875]; 1049; 1055; 6.485888;
CCACTAG; 0.36621; 0.17813;
TYR 1500KB upstream region; USF-1 [T00875]; 1364; 1370; 5.121209;
CCACATC; 0.73242; 0.41833;
TYR 1500KB upstream region; LEF-1 [T02905]; 245; 252; 7.720010;
GGTCAAAG; 0.16022; 0.10553;
TYR 1500KB upstream region; LEF-1 [T02905]; 973; 980; 1.835267;
CTTTGCTT; 0.13733; 0.10092;
TYR 1500KB upstream region; LEF-1 [T02905]; 1332; 1339; 2.021471;
AACCAAAG; 0.13733; 0.10092;
TYR 1500KB upstream region; LEF-1 [T02905]; 1423; 1430; 8.011275;

CTTTGCAT; 0.22888; 0.16519;
TYR 1500KB upstream region; FOXD3 [T02290]; 17; 24; 2.060269; TCATGTTT; 0.22888; 0.22054;
TYR 1500KB upstream region; FOXD3 [T02290]; 284; 291; 0.461834; AATTGTTT; 0.09155; 0.11425;
TYR 1500KB upstream region; FOXD3 [T02290]; 325; 332; 0.184733; AAACAATA; 0.09155; 0.11443;
TYR 1500KB upstream region; FOXD3 [T02290]; 463; 470; 3.910247; CAGTGTTT; 0.11444; 0.10419;
TYR 1500KB upstream region; FOXD3 [T02290]; 595; 602; 4.835029; AAACAGCA; 0.18311; 0.12873;
TYR 1500KB upstream region; FOXD3 [T02290]; 1013; 1020; 4.370924; CTCTGTTT; 0.13733; 0.12406;
TYR 1500KB upstream region; FOXD3 [T02290]; 1104; 1111; 4.743818; CTGTGTTT; 0.13733; 0.12406;
TYR 1500KB upstream region; FOXD3 [T04166]; 473; 483; 7.969909; CTTGTTGATAT; 0.00572; 0.00596;
TYR 1500KB upstream region; USF-1 [T00877]; 694; 704; 14.506027; TCATATGATAT; 0.10300; 0.08274;
TYR 1500KB upstream region; CREB [T00989]; 2; 11; 14.841915; TGACTTCACT; 0.07725; 0.03507;
AIM1 1500KB upstream region; POU3F2 [T00630]; 1; 7; 5.710420; AGGATTT; 0.73242; 0.73821;
AIM1 1500KB upstream region; POU3F2 [T00630]; 31; 37; 7.224857; AACATTT; 0.54932; 0.52790;
AIM1 1500KB upstream region; POU3F2 [T00630]; 74; 80; 2.650678; AAATGGA; 0.27466; 0.41195;
AIM1 1500KB upstream region; POU3F2 [T00630]; 80; 86; 5.963103; AAATAAT; 0.73242; 0.98415;
AIM1 1500KB upstream region; POU3F2 [T00630]; 131; 137; 5.710420; AAATCCT; 0.73242; 0.73821;
AIM1 1500KB upstream region; POU3F2 [T00630]; 207; 213; 5.963103; ATTATTT; 0.73242; 0.98415;
AIM1 1500KB upstream region; POU3F2 [T00630]; 228; 234; 8.108417; AAATGGT; 0.18311; 0.15867;
AIM1 1500KB upstream region; POU3F2 [T00630]; 258; 264; 0.505365; AAATAAA; 0.54932; 0.77377;
AIM1 1500KB upstream region; POU3F2 [T00630]; 262; 268; 7.224857; AAATGTT; 0.54932; 0.52790;
AIM1 1500KB upstream region; POU3F2 [T00630]; 269; 275; 7.224857; AAATGTG; 0.54932; 0.52790;
AIM1 1500KB upstream region; POU3F2 [T00630]; 294; 300; 7.327537; GGAATTT; 0.91553; 1.19566;
AIM1 1500KB upstream region; POU3F2 [T00630]; 342; 348; 7.074855; AAATCAC; 0.54932; 0.52790;
AIM1 1500KB upstream region; POU3F2 [T00630]; 361; 367; 5.710420; AAATCCT; 0.73242; 0.73821;
AIM1 1500KB upstream region; POU3F2 [T00630]; 402; 408; 5.963103; ATTATTT; 0.73242; 0.98415;
AIM1 1500KB upstream region; POU3F2 [T00630]; 425; 431; 5.963103; ATAATTT; 0.73242; 0.98415;
AIM1 1500KB upstream region; POU3F2 [T00630]; 432; 438; 2.272484; TAAATTT; 0.27466; 0.41195;
AIM1 1500KB upstream region; POU3F2 [T00630]; 433; 439; 7.730221;

AAATTTT; 0.91553; 1.19566;
AIM1 1500KB upstream region; POU3F2 [T00630]; 440; 446; 0.000000;
TGCATTT; 0.18311; 0.21080;
AIM1 1500KB upstream region; POU3F2 [T00630]; 460; 466; 7.327537;
GGAATTT; 0.91553; 1.19566;
AIM1 1500KB upstream region; POU3F2 [T00630]; 478; 484; 3.156043;
AAATTGA; 0.27466; 0.33133;
AIM1 1500KB upstream region; POU3F2 [T00630]; 486; 492; 2.650678;
AAATGGA; 0.27466; 0.41195;
AIM1 1500KB upstream region; POU3F2 [T00630]; 546; 552; 7.327537;
GTAATTT; 0.91553; 1.19566;
AIM1 1500KB upstream region; POU3F2 [T00630]; 556; 562; 8.613781;
AAATTGT; 0.64087; 0.67044;
AIM1 1500KB upstream region; POU3F2 [T00630]; 659; 665; 1.767119;
TACATTT; 0.18311; 0.24110;
AIM1 1500KB upstream region; POU3F2 [T00630]; 729; 735; 0.505365;
AAATAAA; 0.54932; 0.77377;
AIM1 1500KB upstream region; POU3F2 [T00630]; 733; 739; 9.725533;
AAATCGC; 0.18311; 0.13544;
AIM1 1500KB upstream region; POU3F2 [T00630]; 841; 847; 0.505365;
AAATACA; 0.54932; 0.77377;
AIM1 1500KB upstream region; POU3F2 [T00630]; 869; 875; 0.505365;
AAATAAA; 0.54932; 0.77377;
AIM1 1500KB upstream region; POU3F2 [T00630]; 875; 881; 7.074855;
AAATCCC; 0.54932; 0.52790;
AIM1 1500KB upstream region; POU3F2 [T00630]; 888; 894; 8.841973;
AAATCTC; 0.27466; 0.33169;
AIM1 1500KB upstream region; POU3F2 [T00630]; 947; 953; 1.767119;
AAATGTA; 0.18311; 0.24110;
AIM1 1500KB upstream region; POU3F2 [T00630]; 954; 960; 9.978215;
GCAATTT; 0.18311; 0.18057;
AIM1 1500KB upstream region; POU3F2 [T00630]; 979; 985; 5.963103;
CTTATTT; 0.73242; 0.98415;
AIM1 1500KB upstream region; POU3F2 [T00630]; 983; 989; 0.505365;
TTTATTT; 0.54932; 0.77377;
AIM1 1500KB upstream region; POU3F2 [T00630]; 996; 1002; 7.730221;
CAAATTT; 0.91553; 1.19566;
AIM1 1500KB upstream region; POU3F2 [T00630]; 997; 1003; 2.272484;
AAATTTA; 0.27466; 0.41195;
AIM1 1500KB upstream region; POU3F2 [T00630]; 1097; 1103; 5.457738;
ATCATTT; 0.73242; 0.73821;
AIM1 1500KB upstream region; POU3F2 [T00630]; 1145; 1151; 9.978215;
GCAATTT; 0.18311; 0.18057;
AIM1 1500KB upstream region; POU3F2 [T00630]; 1222; 1228; 3.156043;
TCAATTT; 0.27466; 0.33133;
AIM1 1500KB upstream region; POU3F2 [T00630]; 1313; 1319; 8.613781;
AAATAGG; 0.64087; 0.67044;
AIM1 1500KB upstream region; USF-1 [T00875]; 52; 58; 5.152973; GAAGTGG;
0.73242; 0.41833;
AIM1 1500KB upstream region; USF-1 [T00875]; 110; 116; 4.610088;
CCACAGG; 0.36621; 0.13317;
AIM1 1500KB upstream region; USF-1 [T00875]; 151; 157; 10.584831;
ACTGTGG; 1.09863; 0.49365;
AIM1 1500KB upstream region; USF-1 [T00875]; 173; 179; 10.685079;

CCACTCC; 1.09863; 0.49365;
AIM1 1500KB upstream region; USF-1 [T00875]; 318; 324; 11.538613;
CCACTCT; 1.09863; 0.59829;
AIM1 1500KB upstream region; USF-1 [T00875]; 580; 586; 10.685079;
TGAGTGG; 1.09863; 0.49365;
AIM1 1500KB upstream region; USF-1 [T00875]; 747; 753; 9.763061;
TCAGTGG; 0.36621; 0.15632;
AIM1 1500KB upstream region; USF-1 [T00875]; 768; 774; 0.000000;
CCACATG; 0.36621; 0.17808;
AIM1 1500KB upstream region; USF-1 [T00875]; 784; 790; 5.563870;
CGGGTGG; 0.36621; 0.13403;
AIM1 1500KB upstream region; USF-1 [T00875]; 1212; 1218; 10.685079;
GGAGTGG; 1.09863; 0.49365;
AIM1 1500KB upstream region; USF-1 [T00875]; 1241; 1247; 11.575332;
CCACGAA; 1.09863; 0.59829;
AIM1 1500KB upstream region; USF-1 [T00875]; 1366; 1372; 11.575332;
TTCGTGG; 1.09863; 0.59829;
AIM1 1500KB upstream region; USF-1 [T00875]; 1407; 1413; 5.152973;
GAGGTGG; 0.73242; 0.41833;
AIM1 1500KB upstream region; Mitf [T01554]; 767; 779; 7.618192;
GCCACATGAATGG; 0.00778; 0.00415;
AIM1 1500KB upstream region; LEF-1 [T02905]; 143; 150; 7.080257;
CTACAAAG; 0.09155; 0.08101;
AIM1 1500KB upstream region; LEF-1 [T02905]; 375; 382; 8.011275;
CTGCAAAG; 0.22888; 0.16519;
AIM1 1500KB upstream region; LEF-1 [T02905]; 673; 680; 8.011275;
ATGCAAAG; 0.22888; 0.16519;
AIM1 1500KB upstream region; LEF-1 [T02905]; 895; 902; 1.835267;
CTTTGCTT; 0.13733; 0.10092;
AIM1 1500KB upstream region; LEF-1 [T02905]; 900; 907; 6.707850;
CTTCAAAG; 0.09155; 0.08561;
AIM1 1500KB upstream region; LEF-1 [T02905]; 1266; 1273; 0.372407;
GAACAAAG; 0.09155; 0.08561;
AIM1 1500KB upstream region; LEF-1 [T02905]; 1486; 1493; 2.021471;
CTTTGGTT; 0.13733; 0.10092;
AIM1 1500KB upstream region; FOXD3 [T02290]; 30; 37; 0.461834; AAACATTT;
0.09155; 0.11425;
AIM1 1500KB upstream region; FOXD3 [T02290]; 165; 172; 1.875536;
AAACATGC; 0.22888; 0.22054;
AIM1 1500KB upstream region; FOXD3 [T02290]; 271; 278; 4.651452;
ATGTGTTT; 0.13733; 0.12406;
AIM1 1500KB upstream region; FOXD3 [T02290]; 415; 422; 1.851876;
AAACATCA; 0.22888; 0.22054;
AIM1 1500KB upstream region; FOXD3 [T02290]; 791; 798; 4.858688;
GCCTGTTT; 0.18311; 0.12873;
AIM1 1500KB upstream region; FOXD3 [T02290]; 847; 854; 1.295405;
AAACATAT; 0.13733; 0.15122;
AIM1 1500KB upstream region; FOXD3 [T02290]; 1048; 1055; 2.060269;
AAACAAGA; 0.22888; 0.22054;
AIM1 1500KB upstream region; FOXD3 [T02290]; 1120; 1127; 1.851876;
AAACAACA; 0.22888; 0.22054;
AIM1 1500KB upstream region; FOXD3 [T04166]; 868; 878; 13.371894;
AAAATAAAAAT; 0.06008; 0.08496;
AIM1 1500KB upstream region; FOXD3 [T04166]; 958; 968; 13.371894;

TTTGTCTTTTT; 0.06008; 0.08496;
AIM1 1500KB upstream region; FOXD3 [T04166]; 979; 989; 14.158476;
CTTATTTATTT; 0.09012; 0.11075;
AIM1 1500KB upstream region; FOXD3 [T04166]; 1093; 1103; 14.945058;
GTTGATCATTT; 0.06008; 0.06359;
AIM1 1500KB upstream region; FOXD3 [T04166]; 1116; 1126; 3.932910;
AAAGAAACAAC; 0.00286; 0.00342;
AIM1 1500KB upstream region; FOXD3 [T04166]; 1119; 1129; 14.158476;
GAAACAACAAC; 0.09012; 0.11075;
AIM1 1500KB upstream region; USF-1 [T00877]; 765; 775; 0.000000;
TTGCCACATGA; 0.00572; 0.00447;
MLANA 1500KB upstream region; POU3F2 [T00630]; 27; 33; 8.613781;
CCAATTT; 0.64087; 0.67044;
MLANA 1500KB upstream region; POU3F2 [T00630]; 60; 66; 8.841973;
GAGATTT; 0.27466; 0.33169;
MLANA 1500KB upstream region; POU3F2 [T00630]; 96; 102; 5.710420;
AAATCCT; 0.73242; 0.73821;
MLANA 1500KB upstream region; POU3F2 [T00630]; 108; 114; 7.327537;
GTAATTT; 0.91553; 1.19566;
MLANA 1500KB upstream region; POU3F2 [T00630]; 117; 123; 5.963103;
AAATAAT; 0.73242; 0.98415;
MLANA 1500KB upstream region; POU3F2 [T00630]; 135; 141; 0.505365;
TGAATTT; 0.54932; 0.77377;
MLANA 1500KB upstream region; POU3F2 [T00630]; 172; 178; 8.613781;
AAATAGT; 0.64087; 0.67044;
MLANA 1500KB upstream region; POU3F2 [T00630]; 221; 227; 1.767119;
AAATGTA; 0.18311; 0.24110;
MLANA 1500KB upstream region; POU3F2 [T00630]; 251; 257; 0.505365;
TGAATTT; 0.54932; 0.77377;
MLANA 1500KB upstream region; POU3F2 [T00630]; 271; 277; 7.477539;
CAGATTT; 0.91553; 1.19566;
MLANA 1500KB upstream region; POU3F2 [T00630]; 290; 296; 1.767119;
AAATGTA; 0.18311; 0.24110;
MLANA 1500KB upstream region; POU3F2 [T00630]; 293; 299; 0.505365;
TGTATTT; 0.54932; 0.77377;
MLANA 1500KB upstream region; POU3F2 [T00630]; 308; 314; 5.963103;
AGTATTT; 0.73242; 0.98415;
MLANA 1500KB upstream region; POU3F2 [T00630]; 317; 323; 2.272484;
TAAATTT; 0.27466; 0.41195;
MLANA 1500KB upstream region; POU3F2 [T00630]; 318; 324; 2.272484;
AAATTTA; 0.27466; 0.41195;
MLANA 1500KB upstream region; POU3F2 [T00630]; 395; 401; 7.327537;
AAATACC; 0.91553; 1.19566;
MLANA 1500KB upstream region; POU3F2 [T00630]; 441; 447; 0.000000;
TTCATTT; 0.18311; 0.21080;
MLANA 1500KB upstream region; POU3F2 [T00630]; 458; 464; 5.457738;
AAATGCT; 0.73242; 0.73821;
MLANA 1500KB upstream region; POU3F2 [T00630]; 631; 637; 5.710420;
AAATCCT; 0.73242; 0.73821;
MLANA 1500KB upstream region; POU3F2 [T00630]; 656; 662; 2.019801;
AAATCTA; 0.18311; 0.24110;
MLANA 1500KB upstream region; POU3F2 [T00630]; 675; 681; 7.224857;
CACATTT; 0.54932; 0.52790;
MLANA 1500KB upstream region; POU3F2 [T00630]; 714; 720; 1.767119;

AAATGTA; 0.18311; 0.24110;
MLANA 1500KB upstream region; POU3F2 [T00630]; 720; 726; 5.457738;
AAATGAT; 0.73242; 0.73821;
MLANA 1500KB upstream region; POU3F2 [T00630]; 760; 766; 9.978215;
GCTATTT; 0.18311; 0.18057;
MLANA 1500KB upstream region; POU3F2 [T00630]; 813; 819; 0.000000;
TTCATTT; 0.18311; 0.21080;
MLANA 1500KB upstream region; POU3F2 [T00630]; 892; 898; 7.327537;
AAATTAC; 0.91553; 1.19566;
MLANA 1500KB upstream region; POU3F2 [T00630]; 899; 905; 0.505365;
TTAATTT; 0.54932; 0.77377;
MLANA 1500KB upstream region; POU3F2 [T00630]; 993; 999; 1.767119;
AAATGTA; 0.18311; 0.24110;
MLANA 1500KB upstream region; POU3F2 [T00630]; 1028; 1034; 7.224857;
AAATGTG; 0.54932; 0.52790;
MLANA 1500KB upstream region; POU3F2 [T00630]; 1094; 1100; 5.963103;
CTTATTT; 0.73242; 0.98415;
MLANA 1500KB upstream region; POU3F2 [T00630]; 1120; 1126; 5.963103;
AAATAAG; 0.73242; 0.98415;
MLANA 1500KB upstream region; POU3F2 [T00630]; 1214; 1220; 9.978215;
AAATTGC; 0.18311; 0.18057;
MLANA 1500KB upstream region; POU3F2 [T00630]; 1235; 1241; 5.710420;
AAATCCT; 0.73242; 0.73821;
MLANA 1500KB upstream region; POU3F2 [T00630]; 1243; 1249; 7.327537;
GTAATTT; 0.91553; 1.19566;
MLANA 1500KB upstream region; POU3F2 [T00630]; 1291; 1297; 7.074855;
GTGATTT; 0.54932; 0.52790;
MLANA 1500KB upstream region; POU3F2 [T00630]; 1315; 1321; 7.327537;
GGAATTT; 0.91553; 1.19566;
MLANA 1500KB upstream region; POU3F2 [T00630]; 1326; 1332; 0.000000;
TTCATTT; 0.18311; 0.21080;
MLANA 1500KB upstream region; POU3F2 [T00630]; 1354; 1360; 0.252682;
TGGATTT; 0.54932; 0.77377;
MLANA 1500KB upstream region; POU3F2 [T00630]; 1386; 1392; 9.094656;
GAAATTT; 0.27466; 0.33169;
MLANA 1500KB upstream region; POU3F2 [T00630]; 1387; 1393; 9.094656;
AAATTTC; 0.27466; 0.33169;
MLANA 1500KB upstream region; POU3F2 [T00630]; 1414; 1420; 7.074855;
AAATCCC; 0.54932; 0.52790;
MLANA 1500KB upstream region; POU3F2 [T00630]; 1440; 1446; 0.252682;
TGGATTT; 0.54932; 0.77377;
MLANA 1500KB upstream region; POU3F2 [T00630]; 1473; 1479; 7.730221;
AATATTT; 0.91553; 1.19566;
MLANA 1500KB upstream region; USF-1 [T00875]; 70; 76; 4.641852; CCAGTGG;
0.36621; 0.13317;
MLANA 1500KB upstream region; USF-1 [T00875]; 102; 108; 9.731297;
TCTGTGG; 0.36621; 0.15583;
MLANA 1500KB upstream region; USF-1 [T00875]; 351; 357; 5.152973;
TAAGTGG; 0.73242; 0.41833;
MLANA 1500KB upstream region; USF-1 [T00875]; 469; 475; 5.974743;
AACGTGG; 0.36621; 0.23847;
MLANA 1500KB upstream region; USF-1 [T00875]; 1373; 1379; 12.428866;
CCACAAT; 0.36621; 0.23857;
MLANA 1500KB upstream region; LEF-1 [T02905]; 280; 287; 8.011275;

ATGCAAAG; 0.22888; 0.16519;
MLANA 1500KB upstream region; LEF-1 [T02905]; 433; 440; 7.080257;
ATACAAAG; 0.09155; 0.08101;
MLANA 1500KB upstream region; LEF-1 [T02905]; 590; 597; 7.205343;
GCTCAAAG; 0.09155; 0.08101;
MLANA 1500KB upstream region; LEF-1 [T02905]; 692; 699; 8.197478;
CTCCAAAG; 0.22888; 0.16519;
MLANA 1500KB upstream region; LEF-1 [T02905]; 909; 916; 2.021471;
CACCAAAG; 0.13733; 0.10092;
MLANA 1500KB upstream region; LEF-1 [T02905]; 950; 957; 8.197478;
CTCCAAAG; 0.22888; 0.16519;
MLANA 1500KB upstream region; FOXD3 [T02290]; 420; 427; 5.207923;
AAACACCA; 0.18311; 0.12873;
MLANA 1500KB upstream region; FOXD3 [T02290]; 512; 519; 2.983153;
AAACAGTC; 0.04578; 0.04281;
MLANA 1500KB upstream region; FOXD3 [T02290]; 600; 607; 4.001457;
AAACAGAA; 0.11444; 0.10419;
MLANA 1500KB upstream region; FOXD3 [T02290]; 724; 731; 0.000000;
GATTGTTT; 0.09155; 0.11443;
MLANA 1500KB upstream region; FOXD3 [T02290]; 785; 792; 0.461834;
AATTGTTT; 0.09155; 0.11425;
MLANA 1500KB upstream region; FOXD3 [T02290]; 800; 807; 1.875536;
GCTTGTTT; 0.22888; 0.22054;
MLANA 1500KB upstream region; FOXD3 [T02290]; 807; 814; 4.001457;
TTCTGTTT; 0.11444; 0.10419;
MLANA 1500KB upstream region; FOXD3 [T02290]; 834; 841; 0.554200;
CATTGTTT; 0.09155; 0.11425;
MLANA 1500KB upstream region; FOXD3 [T02290]; 1300; 1307; 0.554200;
CATTGTTT; 0.09155; 0.11425;
MLANA 1500KB upstream region; FOXD3 [T02290]; 1460; 1467; 2.429736;
CCTTGTTT; 0.09155; 0.08561;
MLANA 1500KB upstream region; FOXD3 [T04166]; 416; 426; 13.371894;
AAAAAAACACC; 0.06008; 0.08496;
MLANA 1500KB upstream region; FOXD3 [T04166]; 1322; 1332; 14.945058;
TTTATTCATTT; 0.06008; 0.06359;
MLANA 1500KB upstream region; FOXD3 [T04166]; 1424; 1434; 10.329655;
TTTGTTAGTAT; 0.00858; 0.00778;
MLANA 1500KB upstream region; FOXD3 [T04166]; 1461; 1471; 14.158476;
CTTGTTTTTA; 0.09012; 0.11075;
MLANA 1500KB upstream region; USF-1 [T00877]; 1478; 1488; 14.506027;
TTATTACATGT; 0.10300; 0.08274;
MC1R 1500KB upstream region; POU3F2 [T00630]; 5; 11; 2.272484; AAATATA;
0.27466; 0.41195;
MC1R 1500KB upstream region; POU3F2 [T00630]; 11; 17; 9.978215; AAATTGC;
0.18311; 0.18057;
MC1R 1500KB upstream region; POU3F2 [T00630]; 235; 241; 6.822173;
GGCATTT; 0.54932; 0.52790;
MC1R 1500KB upstream region; POU3F2 [T00630]; 262; 268; 5.963103;
AAATTAT; 0.73242; 0.98415;
MC1R 1500KB upstream region; POU3F2 [T00630]; 302; 308; 5.963103;
AGAATTT; 0.73242; 0.98415;
MC1R 1500KB upstream region; POU3F2 [T00630]; 376; 382; 7.327537;
AAATTCC; 0.91553; 1.19566;
MC1R 1500KB upstream region; POU3F2 [T00630]; 522; 528; 5.457738;

AAATGCT; 0.73242; 0.73821;
MC1R 1500KB upstream region; POU3F2 [T00630]; 613; 619; 2.272484;
AAATATA; 0.27466; 0.41195;
MC1R 1500KB upstream region; POU3F2 [T00630]; 831; 837; 0.505365;
TGTATTT; 0.54932; 0.77377;
MC1R 1500KB upstream region; POU3F2 [T00630]; 1010; 1016; 2.272484;
TAAATTT; 0.27466; 0.41195;
MC1R 1500KB upstream region; POU3F2 [T00630]; 1011; 1017; 7.730221;
AAATTTT; 0.91553; 1.19566;
MC1R 1500KB upstream region; POU3F2 [T00630]; 1024; 1030; 3.156043;
TCTATTT; 0.27466; 0.33133;
MC1R 1500KB upstream region; POU3F2 [T00630]; 1044; 1050; 0.505365;
AAATTCA; 0.54932; 0.77377;
MC1R 1500KB upstream region; POU3F2 [T00630]; 1328; 1334; 7.074855;
GGGATTT; 0.54932; 0.52790;
MC1R 1500KB upstream region; POU3F2 [T00630]; 1399; 1405; 7.327537;
AAATACC; 0.91553; 1.19566;
MC1R 1500KB upstream region; POU3F2 [T00630]; 1418; 1424; 7.074855;
AAATCAC; 0.54932; 0.52790;
MC1R 1500KB upstream region; POU3F2 [T00630]; 1446; 1452; 7.477539;
AAATCTG; 0.91553; 1.19566;
MC1R 1500KB upstream region; POU3F2 [T00630]; 1464; 1470; 5.963103;
CTTATTT; 0.73242; 0.98415;
MC1R 1500KB upstream region; USF-1 [T00875]; 74; 80; 0.031764; CCACCTG;
0.36621; 0.17808;
MC1R 1500KB upstream region; USF-1 [T00875]; 114; 120; 6.006507;
CCACCTT; 0.36621; 0.23847;
MC1R 1500KB upstream region; USF-1 [T00875]; 168; 174; 6.006507;
AAGGTGG; 0.36621; 0.23847;
MC1R 1500KB upstream region; USF-1 [T00875]; 203; 209; 5.121209;
CCACATC; 0.73242; 0.41833;
MC1R 1500KB upstream region; USF-1 [T00875]; 402; 408; 11.607096;
CCACCAC; 1.09863; 0.59829;
MC1R 1500KB upstream region; USF-1 [T00875]; 405; 411; 10.685079;
CCACCCA; 1.09863; 0.49365;
MC1R 1500KB upstream region; USF-1 [T00875]; 514; 520; 9.763061;
TCAGTGG; 0.36621; 0.15632;
MC1R 1500KB upstream region; USF-1 [T00875]; 692; 698; 11.506849;
AGCGTGG; 1.09863; 0.59829;
MC1R 1500KB upstream region; USF-1 [T00875]; 718; 724; 5.563870;
CCACCCG; 0.36621; 0.13403;
MC1R 1500KB upstream region; USF-1 [T00875]; 721; 727; 4.610088;
CCCGTGG; 0.36621; 0.13317;
MC1R 1500KB upstream region; USF-1 [T00875]; 729; 735; 5.974743;
CCACATT; 0.36621; 0.23847;
MC1R 1500KB upstream region; USF-1 [T00875]; 736; 742; 11.607096;
CCACCAC; 1.09863; 0.59829;
MC1R 1500KB upstream region; USF-1 [T00875]; 739; 745; 10.685079;
CCACTCC; 1.09863; 0.49365;
MC1R 1500KB upstream region; USF-1 [T00875]; 744; 750; 6.006507;
CCACCTT; 0.36621; 0.23847;
MC1R 1500KB upstream region; USF-1 [T00875]; 821; 827; 10.616595;
CCACTGT; 1.09863; 0.49365;
MC1R 1500KB upstream region; USF-1 [T00875]; 1101; 1107; 5.152973;

CCACTTC; 0.73242; 0.41833;
MC1R 1500KB upstream region; USF-1 [T00875]; 1206; 1212; 11.607096;
CCACCAC; 1.09863; 0.59829;
MC1R 1500KB upstream region; USF-1 [T00875]; 1209; 1215; 6.006507;
CCACTTT; 0.36621; 0.23847;
MC1R 1500KB upstream region; USF-1 [T00875]; 1251; 1257; 6.454123;
CTTGTGG; 0.36621; 0.17813;
MC1R 1500KB upstream region; USF-1 [T00875]; 1484; 1490; 11.575332;
CCACAAA; 1.09863; 0.59829;
MC1R 1500KB upstream region; Mitf [T01554]; 917; 929; 7.123746;
ATCATCATGTGCA; 0.00595; 0.00327;
MC1R 1500KB upstream region; USF1 [T00874]; 719; 727; 8.313905;
CACCCGTGG; 0.22316; 0.08113;
MC1R 1500KB upstream region; LEF-1 [T02905]; 163; 170; 0.904249;
CAACAAAG; 0.06866; 0.06728;
MC1R 1500KB upstream region; LEF-1 [T02905]; 186; 193; 9.023435;
CTTTGCCC; 0.18311; 0.09642;
MC1R 1500KB upstream region; LEF-1 [T02905]; 892; 899; 6.707850;
TTTCAAAG; 0.09155; 0.08561;
MC1R 1500KB upstream region; LEF-1 [T02905]; 1002; 1009; 1.835267;
CTTTGCTG; 0.13733; 0.10092;
MC1R 1500KB upstream region; LEF-1 [T02905]; 1085; 1092; 8.251852;
CTTTGACA; 0.18311; 0.12147;
MC1R 1500KB upstream region; LEF-1 [T02905]; 1155; 1162; 0.372407;
GAACAAAG; 0.09155; 0.08561;
MC1R 1500KB upstream region; LEF-1 [T02905]; 1238; 1245; 1.489628;
GACCAAAG; 0.04578; 0.02752;
MC1R 1500KB upstream region; LEF-1 [T02905]; 1280; 1287; 1.303425;
CTTTGCTC; 0.04578; 0.02752;
MC1R 1500KB upstream region; LEF-1 [T02905]; 1297; 1304; 0.904249;
CTTTGTTG; 0.06866; 0.06728;
MC1R 1500KB upstream region; LEF-1 [T02905]; 1484; 1491; 8.109591;
CCACAAAG; 0.22888; 0.16519;
MC1R 1500KB upstream region; FOXD3 [T02290]; 344; 351; 3.816724;
AAACAGAC; 0.11444; 0.10419;
MC1R 1500KB upstream region; FOXD3 [T02290]; 776; 783; 5.043422;
TCCTGTTT; 0.18311; 0.12873;
MC1R 1500KB upstream region; FOXD3 [T02290]; 867; 874; 2.983153;
GACTGTTT; 0.04578; 0.04281;
MC1R 1500KB upstream region; FOXD3 [T02290]; 1379; 1386; 3.356047;
GAGTGTTT; 0.09155; 0.08583;
MC1R 1500KB upstream region; FOXD3 [T02290]; 1489; 1496; 3.817880;
AAGTGTTT; 0.11444; 0.10419;
MC1R 1500KB upstream region; USF-1 [T00877]; 921; 931; 9.029386;
TCATGTGCATC; 0.05007; 0.02443;
MC1R 1500KB upstream region; USF-1 [T00877]; 1309; 1319; 8.109390;
GCATGTGATCT; 0.02289; 0.01079;
RAB27A 1500KB upstream region; POU3F2 [T00630]; 40; 46; 7.224857;
CACATTT; 0.54932; 0.52790;
RAB27A 1500KB upstream region; POU3F2 [T00630]; 177; 183; 5.457738;
CTCATTT; 0.73242; 0.73821;
RAB27A 1500KB upstream region; POU3F2 [T00630]; 268; 274; 7.327537;
AAATAAC; 0.91553; 1.19566;
RAB27A 1500KB upstream region; POU3F2 [T00630]; 283; 289; 5.710420;

AGGATTT; 0.73242; 0.73821;
RAB27A 1500KB upstream region; POU3F2 [T00630]; 367; 373; 0.252682;
TTGATTT; 0.54932; 0.77377;
RAB27A 1500KB upstream region; POU3F2 [T00630]; 500; 506; 5.963103;
AGAATTT; 0.73242; 0.98415;
RAB27A 1500KB upstream region; POU3F2 [T00630]; 621; 627; 7.327537;
AAATTCC; 0.91553; 1.19566;
RAB27A 1500KB upstream region; POU3F2 [T00630]; 1119; 1125; 5.710420;
CTGATTT; 0.73242; 0.73821;
RAB27A 1500KB upstream region; POU3F2 [T00630]; 1134; 1140; 9.472851;
AAATGGC; 0.18311; 0.13544;
RAB27A 1500KB upstream region; POU3F2 [T00630]; 1477; 1483; 5.710420;
AAATCCT; 0.73242; 0.73821;
RAB27A 1500KB upstream region; USF-1 [T00875]; 19; 25; 0.000000;
CCACGTG; 0.36621; 0.17808;
RAB27A 1500KB upstream region; USF-1 [T00875]; 158; 164; 5.152973;
CCACTTC; 0.73242; 0.41833;
RAB27A 1500KB upstream region; USF-1 [T00875]; 211; 217; 9.731297;
GCTGTGG; 0.36621; 0.15583;
RAB27A 1500KB upstream region; USF-1 [T00875]; 310; 316; 12.460630;
CCACCAT; 0.36621; 0.23857;
RAB27A 1500KB upstream region; USF-1 [T00875]; 392; 398; 9.731297;
CCACAGA; 0.36621; 0.15583;
RAB27A 1500KB upstream region; USF-1 [T00875]; 408; 414; 11.575332;
TTTGTGG; 1.09863; 0.59829;
RAB27A 1500KB upstream region; USF-1 [T00875]; 423; 429; 11.575332;
CCACAAA; 1.09863; 0.59829;
RAB27A 1500KB upstream region; USF-1 [T00875]; 429; 435; 10.584831;
ACCGTGG; 1.09863; 0.49365;
RAB27A 1500KB upstream region; USF-1 [T00875]; 493; 499; 5.121209;
GATGTGG; 0.73242; 0.41833;
RAB27A 1500KB upstream region; USF-1 [T00875]; 517; 523; 5.152973;
GAAGTGG; 0.73242; 0.41833;
RAB27A 1500KB upstream region; USF-1 [T00875]; 639; 645; 0.000000;
CCACATG; 0.36621; 0.17808;
RAB27A 1500KB upstream region; USF-1 [T00875]; 705; 711; 0.000000;
CCACATG; 0.36621; 0.17808;
RAB27A 1500KB upstream region; USF-1 [T00875]; 858; 864; 9.731297;
GCTGTGG; 0.36621; 0.15583;
RAB27A 1500KB upstream region; USF-1 [T00875]; 1072; 1078; 11.538613;
CCACTCT; 1.09863; 0.59829;
RAB27A 1500KB upstream region; USF-1 [T00875]; 1178; 1184; 5.152973;
CCACTTC; 0.73242; 0.41833;
RAB27A 1500KB upstream region; USF-1 [T00875]; 1382; 1388; 10.584831;
CCACGGT; 1.09863; 0.49365;
RAB27A 1500KB upstream region; USF-1 [T00875]; 1400; 1406; 9.763061;
GCAGTGG; 0.36621; 0.15632;
RAB27A 1500KB upstream region; USF-1 [T00875]; 1491; 1497; 10.584831;
ACTGTGG; 1.09863; 0.49365;
RAB27A 1500KB upstream region; USF1 [T00874]; 18; 26; 2.334845;
TCCACGTGC; 0.06294; 0.02694;
RAB27A 1500KB upstream region; USF1 [T00874]; 19; 27; 2.100004;
CCACGTGCA; 0.07439; 0.03130;
RAB27A 1500KB upstream region; USF1 [T00874]; 800; 808; 8.514439;

CTCGCGTGC; 0.22316; 0.08113;
RAB27A 1500KB upstream region; LEF-1 [T02905]; 665; 672; 8.197478; CTTTGGAA; 0.22888; 0.16519;
RAB27A 1500KB upstream region; LEF-1 [T02905]; 1042; 1049; 7.479433; CTTTGCAC; 0.16022; 0.10553;
RAB27A 1500KB upstream region; LEF-1 [T02905]; 1049; 1056; 9.226813; CTTTGGGG; 0.18311; 0.09642;
RAB27A 1500KB upstream region; LEF-1 [T02905]; 1226; 1233; 8.011275; CTTTGCAG; 0.22888; 0.16519;
RAB27A 1500KB upstream region; LEF-1 [T02905]; 1234; 1241; 0.904249; CAACAAAG; 0.06866; 0.06728;
RAB27A 1500KB upstream region; FOXD3 [T02290]; 325; 332; 2.337369; AAACAAGT; 0.09155; 0.08561;
RAB27A 1500KB upstream region; FOXD3 [T02290]; 403; 410; 5.412889; CCCTGTTT; 0.13733; 0.10109;
RAB27A 1500KB upstream region; FOXD3 [T02290]; 473; 480; 3.540780; AAACACTA; 0.09155; 0.08583;
RAB27A 1500KB upstream region; FOXD3 [T02290]; 540; 547; 1.851876; AAACATCA; 0.22888; 0.22054;
RAB27A 1500KB upstream region; FOXD3 [T02290]; 916; 923; 3.816724; AAACAGAC; 0.11444; 0.10419;
RAB27A 1500KB upstream region; FOXD3 [T02290]; 1095; 1102; 5.023189; AAACACCC; 0.18311; 0.12873;
RAB27A 1500KB upstream region; FOXD3 [T02290]; 1138; 1145; 4.650295; GGCTGTTT; 0.13733; 0.12406;
RAB27A 1500KB upstream region; FOXD3 [T04166]; 419; 429; 14.158476; AAAACCACAAA; 0.09012; 0.11075;
RAB27A 1500KB upstream region; FOXD3 [T04166]; 539; 549; 14.945058; AAAACATCAAG; 0.06008; 0.06359;
RAB27A 1500KB upstream region; USF-1 [T00877]; 477; 487; 10.883360; ACTACACATGC; 0.02038; 0.00808;
RAB27A 1500KB upstream region; USF-1 [T00877]; 636; 646; 1.386985; TTACCACATGG; 0.02003; 0.01279;
RAB27A 1500KB upstream region; USF-1 [T00877]; 702; 712; 8.802883; TCCCCACATGC; 0.05007; 0.02443;
RAB27A 1500KB upstream region; CREB [T00989]; 1081; 1090; 9.084806; TGACGTATGA; 0.05150; 0.03637;
RAB27A 1500KB upstream region; CREB [T00163]; 1081; 1089; 1.509908; TGACGTATG; 0.06866; 0.04350;
RAB27A 1500KB upstream region; CREB [T00164]; 1081; 1089; 4.825740; TGACGTATG; 0.08583; 0.05598;
MITF 1500bp upstream region; POU3F2 [T00630]; 0; 6; 1.767119; AAATGTA; 0.18311; 0.24110;
MITF 1500bp upstream region; POU3F2 [T00630]; 38; 44; 7.477539; AAGATTT; 0.91553; 1.19566;
MITF 1500bp upstream region; POU3F2 [T00630]; 42; 48; 0.505365; TTTATTT; 0.54932; 0.77377;
MITF 1500bp upstream region; POU3F2 [T00630]; 102; 108; 5.963103; ATTATTT; 0.73242; 0.98415;
MITF 1500bp upstream region; POU3F2 [T00630]; 113; 119; 7.327537; GGAATTT; 0.91553; 1.19566;
MITF 1500bp upstream region; POU3F2 [T00630]; 148; 154; 5.963103; AAATAAT; 0.73242; 0.98415;
MITF 1500bp upstream region; POU3F2 [T00630]; 156; 162; 8.108417;

CCCATTT; 0.18311; 0.15867;
MITF 1500bp upstream region; POU3F2 [T00630]; 349; 355; 5.963103;
CTTATTT; 0.73242; 0.98415;
MITF 1500bp upstream region; POU3F2 [T00630]; 356; 362; 7.730221;
AAAATTT; 0.91553; 1.19566;
MITF 1500bp upstream region; POU3F2 [T00630]; 357; 363; 9.094656;
AAATTTC; 0.27466; 0.33169;
MITF 1500bp upstream region; POU3F2 [T00630]; 389; 395; 5.963103;
AAATTAT; 0.73242; 0.98415;
MITF 1500bp upstream region; POU3F2 [T00630]; 391; 397; 5.963103;
ATTATTT; 0.73242; 0.98415;
MITF 1500bp upstream region; POU3F2 [T00630]; 432; 438; 1.767119;
TACATTT; 0.18311; 0.24110;
MITF 1500bp upstream region; POU3F2 [T00630]; 451; 457; 8.613781;
AAATTGT; 0.64087; 0.67044;
MITF 1500bp upstream region; POU3F2 [T00630]; 459; 465; 2.650678;
TCCATTT; 0.27466; 0.41195;
MITF 1500bp upstream region; POU3F2 [T00630]; 468; 474; 7.730221;
CATATTT; 0.91553; 1.19566;
MITF 1500bp upstream region; POU3F2 [T00630]; 514; 520; 8.108417;
CCCATTT; 0.18311; 0.15867;
MITF 1500bp upstream region; POU3F2 [T00630]; 550; 556; 5.457738;
AAATGCT; 0.73242; 0.73821;
MITF 1500bp upstream region; POU3F2 [T00630]; 617; 623; 5.457738;
AAATGCT; 0.73242; 0.73821;
MITF 1500bp upstream region; POU3F2 [T00630]; 781; 787; 7.327537;
AAATTCC; 0.91553; 1.19566;
MITF 1500bp upstream region; POU3F2 [T00630]; 1041; 1047; 0.505365;
AAATAAA; 0.54932; 0.77377;
MITF 1500bp upstream region; POU3F2 [T00630]; 1074; 1080; 2.019801;
AAATCTA; 0.18311; 0.24110;
MITF 1500bp upstream region; POU3F2 [T00630]; 1105; 1111; 2.272484;
TATATTT; 0.27466; 0.41195;
MITF 1500bp upstream region; POU3F2 [T00630]; 1115; 1121; 2.650678;
TCCATTT; 0.27466; 0.41195;
MITF 1500bp upstream region; POU3F2 [T00630]; 1154; 1160; 5.710420;
AAATCAG; 0.73242; 0.73821;
MITF 1500bp upstream region; POU3F2 [T00630]; 1220; 1226; 0.252682;
AAATCAA; 0.54932; 0.77377;
MITF 1500bp upstream region; POU3F2 [T00630]; 1337; 1343; 0.252682;
AAATCCA; 0.54932; 0.77377;
MITF 1500bp upstream region; POU3F2 [T00630]; 1365; 1371; 6.822173;
GGCATTT; 0.54932; 0.52790;
MITF 1500bp upstream region; POU3F2 [T00630]; 1454; 1460; 7.730221;
AATATTT; 0.91553; 1.19566;
MITF 1500bp upstream region; USF-1 [T00875]; 108; 114; 9.763061;
TCAGTGG; 0.36621; 0.15632;
MITF 1500bp upstream region; USF-1 [T00875]; 835; 841; 9.763061;
CCACTGA; 0.36621; 0.15632;
MITF 1500bp upstream region; USF-1 [T00875]; 882; 888; 9.763061;
CCACTGA; 0.36621; 0.15632;
MITF 1500bp upstream region; USF-1 [T00875]; 1351; 1357; 10.653315;
CCACACC; 1.09863; 0.49365;
MITF 1500bp upstream region; USF-1 [T00875]; 1396; 1402; 5.152973;

CCACTTC; 0.73242; 0.41833;
MITF 1500bp upstream region; Mitf [T01554]; 501; 513; 14.067903; AGCACCTGACGTG; 0.01502; 0.00638;
MITF 1500bp upstream region; LEF-1 [T02905]; 336; 343; 6.707850; CTTTGAAA; 0.09155; 0.08561;
MITF 1500bp upstream region; LEF-1 [T02905]; 520; 527; 8.109591; TCACAAAG; 0.22888; 0.16519;
MITF 1500bp upstream region; LEF-1 [T02905]; 577; 584; 9.209639; CTTTGGCC; 0.18311; 0.09642;
MITF 1500bp upstream region; LEF-1 [T02905]; 638; 645; 8.624259; AGACAAAG; 0.18311; 0.12147;
MITF 1500bp upstream region; LEF-1 [T02905]; 946; 953; 7.205343; CTTTGAGC; 0.09155; 0.08101;
MITF 1500bp upstream region; LEF-1 [T02905]; 1221; 1228; 0.531842; AATCAAAG; 0.09155; 0.08561;
MITF 1500bp upstream region; LEF-1 [T02905]; 1380; 1387; 8.011275; TTGCAAAG; 0.22888; 0.16519;
MITF 1500bp upstream region; LEF-1 [T02905]; 1424; 1431; 9.226813; TCCCAAAG; 0.18311; 0.09642;
MITF 1500bp upstream region; FOXD3 [T02290]; 59; 66; 5.412889; CCCTGTTT; 0.13733; 0.10109;
MITF 1500bp upstream region; FOXD3 [T02290]; 85; 92; 4.835029; TGCTGTTT; 0.18311; 0.12873;
MITF 1500bp upstream region; FOXD3 [T02290]; 137; 144; 0.184733; AAACATTA; 0.09155; 0.11443;
MITF 1500bp upstream region; FOXD3 [T02290]; 452; 459; 0.461834; AATTGTTT; 0.09155; 0.11425;
MITF 1500bp upstream region; FOXD3 [T02290]; 557; 564; 2.060269; TCTTGTTT; 0.22888; 0.22054;
MITF 1500bp upstream region; FOXD3 [T02290]; 1318; 1325; 4.370924; AAACAGAG; 0.13733; 0.12406;
MITF 1500bp upstream region; FOXD3 [T02290]; 1484; 1491; 5.207923; TGGTGTTT; 0.18311; 0.12873;
MITF 1500bp upstream region; FOXD3 [T04166]; 126; 136; 14.158476; GAAAGAACAAT; 0.09012; 0.11075;
MITF 1500bp upstream region; FOXD3 [T04166]; 1169; 1179; 13.371894; AAAAAAAGAAA; 0.06008; 0.08496;
MITF 1500bp upstream region; USF-1 [T00877]; 1403; 1413; 2.080477; CTAGCACATGC; 0.02003; 0.01131;
MITF 1500bp upstream region; CREB [T00989]; 507; 516; 10.487463; TGACGTGCCC; 0.07725; 0.03337;
MITF 1500bp upstream region; CREB [T00163]; 507; 515; 2.041286; TGACGTGCC; 0.13733; 0.07620;
MITF 1500bp upstream region; CREB [T00164]; 507; 515; 5.639863; TGACGTGCC; 0.09727; 0.05069;
OA1 1500KB upstream region; POU3F2 [T00630]; 156; 162; 9.978215; GCAATTT; 0.18311; 0.18057;
OA1 1500KB upstream region; POU3F2 [T00630]; 164; 170; 8.613781; AAATTGT; 0.64087; 0.67044;
OA1 1500KB upstream region; POU3F2 [T00630]; 357; 363; 0.252682; TGGATTT; 0.54932; 0.77377;
OA1 1500KB upstream region; POU3F2 [T00630]; 608; 614; 8.841973; AAATCTC; 0.27466; 0.33169;
OA1 1500KB upstream region; POU3F2 [T00630]; 645; 651; 5.710420;

AGGATTT; 0.73242; 0.73821;
OA1 1500KB upstream region; POU3F2 [T00630]; 719; 725; 8.613781; CCAATTT; 0.64087; 0.67044;
OA1 1500KB upstream region; POU3F2 [T00630]; 961; 967; 8.361098; ACGATTT; 0.64087; 0.67044;
OA1 1500KB upstream region; USF-1 [T00875]; 207; 213; 10.653315; CCACACA; 1.09863; 0.49365;
OA1 1500KB upstream region; USF-1 [T00875]; 296; 302; 6.485888; CCACTAG; 0.36621; 0.17813;
OA1 1500KB upstream region; USF-1 [T00875]; 373; 379; 5.121209; GATGTGG; 0.73242; 0.41833;
OA1 1500KB upstream region; USF-1 [T00875]; 486; 492; 11.607096; TTAGTGG; 1.09863; 0.59829;
OA1 1500KB upstream region; USF-1 [T00875]; 564; 570; 10.653315; GGCGTGG; 1.09863; 0.49365;
OA1 1500KB upstream region; USF-1 [T00875]; 630; 636; 0.000000; CCACGTG; 0.36621; 0.17808;
OA1 1500KB upstream region; USF-1 [T00875]; 839; 845; 5.563870; CCACCCG; 0.36621; 0.13403;
OA1 1500KB upstream region; USF-1 [T00875]; 1354; 1360; 5.152973; CCACTTC; 0.73242; 0.41833;
OA1 1500KB upstream region; USF-1 [T00875]; 1362; 1368; 0.000000; CCACATG; 0.36621; 0.17808;
OA1 1500KB upstream region; USF-1 [T00875]; 1466; 1472; 10.685079; CCACCCC; 1.09863; 0.49365;
OA1 1500KB upstream region; Mitf [T01554]; 1361; 1373; 6.507668; GCCACATGATCCG; 0.00460; 0.00262;
OA1 1500KB upstream region; USF1 [T00874]; 629; 637; 1.761227; ACCACGTGC; 0.07439; 0.03130;
OA1 1500KB upstream region; USF1 [T00874]; 630; 638; 2.100004; CCACGTGCA; 0.07439; 0.03130;
OA1 1500KB upstream region; USF1 [T00874]; 913; 921; 8.890372; ACACGCGGT; 0.21744; 0.07993;
OA1 1500KB upstream region; USF1 [T00874]; 1377; 1385; 7.441259; GCCGCGTGA; 0.13161; 0.04401;
OA1 1500KB upstream region; LEF-1 [T02905]; 387; 394; 7.665637; GTCCAAAG; 0.16022; 0.10553;
OA1 1500KB upstream region; LEF-1 [T02905]; 473; 480; 7.205343; CTTTGAGC; 0.09155; 0.08101;
OA1 1500KB upstream region; LEF-1 [T02905]; 1416; 1423; 6.548415; GTACAAAG; 0.09155; 0.08561;
OA1 1500KB upstream region; FOXD3 [T02290]; 138; 145; 3.910247; CAGTGTTT; 0.11444; 0.10419;
OA1 1500KB upstream region; FOXD3 [T02290]; 236; 243; 3.910247; AAACACTG; 0.11444; 0.10419;
OA1 1500KB upstream region; FOXD3 [T02290]; 575; 582; 5.112129; AGCTGTTT; 0.18311; 0.12873;
OA1 1500KB upstream region; FOXD3 [T02290]; 604; 611; 1.295405; AAACAAAT; 0.13733; 0.15122;
OA1 1500KB upstream region; FOXD3 [T02290]; 676; 683; 4.651452; AAACACAT; 0.13733; 0.12406;
OA1 1500KB upstream region; FOXD3 [T04166]; 984; 994; 13.371894; AAAAAAAAAAA; 0.06008; 0.08496;
OA1 1500KB upstream region; FOXD3 [T04166]; 985; 995; 14.158476;

AAAAAAAAAAG; 0.09012; 0.11075;
OA1 1500KB upstream region; USF-1 [T00877]; 372; 382; 14.506027;
AGATGTGGTAT; 0.10300; 0.08274;
OA1 1500KB upstream region; USF-1 [T00877]; 1359; 1369; 7.415898;
TCGCCACATGA; 0.03147; 0.01802;
PMEL 1500KB upstream region; POU3F2 [T00630]; 176; 182; 9.472851;
AAATGGC; 0.18311; 0.13544;
PMEL 1500KB upstream region; POU3F2 [T00630]; 221; 227; 0.252682;
AAATCAA; 0.54932; 0.77377;
PMEL 1500KB upstream region; POU3F2 [T00630]; 305; 311; 5.963103;
AGAATTT; 0.73242; 0.98415;
PMEL 1500KB upstream region; POU3F2 [T00630]; 762; 768; 7.730221;
AATATTT; 0.91553; 1.19566;
PMEL 1500KB upstream region; POU3F2 [T00630]; 798; 804; 5.963103;
AAATTAT; 0.73242; 0.98415;
PMEL 1500KB upstream region; POU3F2 [T00630]; 800; 806; 5.963103;
ATTATTT; 0.73242; 0.98415;
PMEL 1500KB upstream region; POU3F2 [T00630]; 900; 906; 1.767119;
AAATGTA; 0.18311; 0.24110;
PMEL 1500KB upstream region; POU3F2 [T00630]; 971; 977; 5.963103;
ATTATTT; 0.73242; 0.98415;
PMEL 1500KB upstream region; POU3F2 [T00630]; 978; 984; 0.252682;
TTGATTT; 0.54932; 0.77377;
PMEL 1500KB upstream region; POU3F2 [T00630]; 985; 991; 8.613781;
AAATTGT; 0.64087; 0.67044;
PMEL 1500KB upstream region; POU3F2 [T00630]; 1043; 1049; 8.613781;
CCAATTT; 0.64087; 0.67044;
PMEL 1500KB upstream region; POU3F2 [T00630]; 1055; 1061; 8.841973;
GAGATTT; 0.27466; 0.33169;
PMEL 1500KB upstream region; POU3F2 [T00630]; 1080; 1086; 8.108417;
AAATGGT; 0.18311; 0.15867;
PMEL 1500KB upstream region; POU3F2 [T00630]; 1173; 1179; 5.963103;
AGAATTT; 0.73242; 0.98415;
PMEL 1500KB upstream region; POU3F2 [T00630]; 1342; 1348; 2.019801;
AAATCTA; 0.18311; 0.24110;
PMEL 1500KB upstream region; POU3F2 [T00630]; 1390; 1396; 5.963103;
AAATTCT; 0.73242; 0.98415;
PMEL 1500KB upstream region; POU3F2 [T00630]; 1428; 1434; 9.472851;
GCCATTT; 0.18311; 0.13544;
PMEL 1500KB upstream region; POU3F2 [T00630]; 1453; 1459; 9.472851;
AAATGGC; 0.18311; 0.13544;
PMEL 1500KB upstream region; USF-1 [T00875]; 1; 7; 11.575332; TTTGTGG;
1.09863; 0.59829;
PMEL 1500KB upstream region; USF-1 [T00875]; 133; 139; 9.731297;
GCTGTGG; 0.36621; 0.15583;
PMEL 1500KB upstream region; USF-1 [T00875]; 141; 147; 6.006507;
AAAGTGG; 0.36621; 0.23847;
PMEL 1500KB upstream region; USF-1 [T00875]; 159; 165; 11.607096;
TTGGTGG; 1.09863; 0.59829;
PMEL 1500KB upstream region; USF-1 [T00875]; 162; 168; 11.607096;
GTGGTGG; 1.09863; 0.59829;
PMEL 1500KB upstream region; USF-1 [T00875]; 366; 372; 10.653315;
TGCGTGG; 1.09863; 0.49365;
PMEL 1500KB upstream region; USF-1 [T00875]; 721; 727; 5.121209;

CCACATC; 0.73242; 0.41833;
PMEL 1500KB upstream region; USF-1 [T00875]; 816; 822; 10.685079;
GGAGTGG; 1.09863; 0.49365;
PMEL 1500KB upstream region; USF-1 [T00875]; 822; 828; 10.685079;
GGAGTGG; 1.09863; 0.49365;
PMEL 1500KB upstream region; USF-1 [T00875]; 910; 916; 9.731297;
CCACAGC; 0.36621; 0.15583;
PMEL 1500KB upstream region; USF-1 [T00875]; 987; 993; 12.428866;
ATTGTGG; 0.36621; 0.23857;
PMEL 1500KB upstream region; USF-1 [T00875]; 1186; 1192; 11.538613;
CCACCCT; 1.09863; 0.59829;
PMEL 1500KB upstream region; USF-1 [T00875]; 1199; 1205; 5.152973;
CCACTTC; 0.73242; 0.41833;
PMEL 1500KB upstream region; USF-1 [T00875]; 1275; 1281; 6.485888;
CCACCAG; 0.36621; 0.17813;
PMEL 1500KB upstream region; USF-1 [T00875]; 1358; 1364; 5.152973;
GAGGTGG; 0.73242; 0.41833;
PMEL 1500KB upstream region; USF-1 [T00875]; 1445; 1451; 10.653315;
CCACACA; 1.09863; 0.49365;
PMEL 1500KB upstream region; Mitf [T01554]; 1306; 1318; 8.542309;
TTGCTCATGTGCA; 0.00706; 0.00394;
PMEL 1500KB upstream region; LEF-1 [T02905]; 90; 97; 9.226813; CCCCAAAG;
0.18311; 0.09642;
PMEL 1500KB upstream region; LEF-1 [T02905]; 137; 144; 9.555277;
TGGCAAAG; 0.13733; 0.07565;
PMEL 1500KB upstream region; LEF-1 [T02905]; 890; 897; 8.092417;
CTTTGTCC; 0.22888; 0.16519;
PMEL 1500KB upstream region; LEF-1 [T02905]; 998; 1005; 9.555277;
CTTTGCCT; 0.13733; 0.07565;
PMEL 1500KB upstream region; LEF-1 [T02905]; 1007; 1014; 8.251852;
CTTTGACT; 0.18311; 0.12147;
PMEL 1500KB upstream region; LEF-1 [T02905]; 1047; 1054; 6.707850;
TTTCAAAG; 0.09155; 0.08561;
PMEL 1500KB upstream region; LEF-1 [T02905]; 1154; 1161; 8.197478;
CTTTGGAA; 0.22888; 0.16519;
PMEL 1500KB upstream region; FOXD3 [T02290]; 80; 87; 1.018305; TTTTGTTT;
0.09155; 0.11446;
PMEL 1500KB upstream region; FOXD3 [T02290]; 151; 158; 4.278557;
ATCTGTTT; 0.13733; 0.12406;
PMEL 1500KB upstream region; FOXD3 [T02290]; 336; 343; 3.910247;
CAGTGTTT; 0.11444; 0.10419;
PMEL 1500KB upstream region; FOXD3 [T02290]; 748; 755; 4.743818;
AAACACAG; 0.13733; 0.12406;
PMEL 1500KB upstream region; FOXD3 [T02290]; 1120; 1127; 5.043422;
TCCTGTTT; 0.18311; 0.12873;
PMEL 1500KB upstream region; FOXD3 [T02290]; 1231; 1238; 4.001457;
TTCTGTTT; 0.11444; 0.10419;
PMEL 1500KB upstream region; FOXD3 [T04166]; 974; 984; 13.371894;
ATTTTTGATTT; 0.06008; 0.08496;
PMEL 1500KB upstream region; USF-1 [T00877]; 1310; 1320; 9.496375;
TCATGTGCAGT; 0.04506; 0.01972;
PMEL 1500KB upstream region; USF-1 [T00877]; 1457; 1467; 10.883360;
GGCTCACATGG; 0.02038; 0.00808;

-- END --

>MITF-promoter-1500bpUpstream-pos23041-24540
aaatgtatgttcatctcaagaaagtcttatataggtataagatttatttggacactgatc
cctgtttagatctctgtcacaacagtgctgtttgcttgcattattatttcagtggaattt
ctggaagaaagaacaataaacattaataaaataattcccatttctaattcccataatgct
cttaaagtaactagatagctatgctgtcctatggtatcctatgctgacctgtcctacctt
atcctaccctaccctaccctaccctaccctaccctaccctaccctatcctggcttgtcct
gtcctgtcctatgtgtgtctttagcctaatctacacctttgaaaactctcttatttaaaa
tttctctcttaagcactaatacatctaataaattattttcagatactacatagaagagtc
accttcactgattacattttttttcaatattaaattgtttccattttacatattttgata
cagttcgcactgtcacccagtagcacctgacgtgcccatttcacaaaggacttttagatg
cttgagaataaaatgcttcttgtttgcctcgacaaaactttggccctattgttgtaatgc
atgcagggtctagaacaaaatgctgcaggagattgtccagacaaagctgcactacctaga
cattcaccagttttctcacaatgttgtcagacagtaattcccctgaatataacattcctg
tcttgctatagcagttctttacctgagctgctgggtgacagccccattgagctggagtag
aaaattcccatagagctggactaggaagttcccattgaactggattagaaagttcccact
gagctctactagaaagttcccattgagttggactagaagttcccactgagctggactaga
ctgctgagttcaggtcctcccttaggcatgaaagccgcctgggtgactttgagccagcta
ctgtctctcagcactactttatagggttgttgctgggaaaaaagtaggcaggagcattat
gtacactgccttgagttgcaaaaataaagacaggatatcagtctaataatcaataaatct
atagggacctcttattgagagggcatatatttgattccattttcagtatgataactgggc
ccctattggttccaaaatcaggccttaccaaaaaaagaaaagaaaaagggaataaaggaa
tcctgactgaaagagtagggaaatcaaagacaacttctgtctccttttgaattagtatcg
tataatcactggggtgctgatcattagaaagactagaacatagctaacttcttgtgttaa
acagagatcaagcccagaaatccatatacatccacacccctgccaggcatttttctacac
ttgcaaaggtttttgcccacttcctagcacatgcaatggccccctcccaaagcctgactc
tttaaactccagctaatatttactgagcagtgatgagtgttctctggtgtttccctcagc

>TYR-promoter-1500bpUpstream-pos19738-21237 (reverese complemented)
aactaaaatctggaagtatatttgtgggcacctttggggcaagatttccaaccaggcaaa
gtgagacttgtatttatttaggtatcacatattgcattgatttctatactatccaactgg
gtggatcaccaaccttgggtggattgctacatgtagaaataatcccatgataagcaaaca
ttcaatgacatgccacagcacaatgcccaacagcctagaaatgataaaaaaggtcaatgt
ggaatataaaccctcccaagtgatttctcagaaggctgctcaaacaaaatggtcttgaga
gacttctcagaagctgctaggagaggtgccatgtgaacctctggaggcaaagtaatctat
aatgtcagggccatgccagaagataggctccaggcccatcatcccctctctggaaacatg
gctgaatcactttggagcagaaggacctaaggtatcctggacctaagttataaagagctt
tataagtcaacaccttattggaagacaaatggcagctaataccacaagcataaaacagat
gctaggttccagcagcagcctccaccattaatagcctaaccacttcattttgtaccacca
actccaccagctttggagtcatcttcaaagtcagccccatgtaaagtgcattatagtagt
ctagccatggcataatgagggcatgagtctcagtgcccatcgccagctccttcctgcacc
gaataaaaaaatttgcaatgactgtttgtctcgcaacatgtctgtgtgtgcatgcatacg
cgtgctttgtgatttccaaacagcaccttggagtttactgttctgtagtccaataaatga
aagctcttgcctatcttcctcacaccctgcttttaaaaaaataattagcgtttatatgc
aacacaattaagtataaatggaactgccatgactgcctttactgctcccccaattgattg
gtatgtgtgtgtctaggaaagttttacacacatttctttctgcctagaaacatacataag
accatctctttctggtcttatgactgaaggactttggatcaactttctttctgatttgtg
ggatttgttgagctcagcctttgaaaagtggagtttttcccccctgtagtcactcaattgt
tcattctcattcctctgcacaagagcccagtgattccctctccaagtaccttggtgtgag
caagcactccgatccagcacgtcaaaagtcatgtgctaggcagaagatgaagttcgaaaa
atgagtctctgtataactgaagtggcaagtgggtaaacgttccttggatgtagccctgag
catgtgataatcagattacagaatgtgtgtatgtgtcaagagagagaagcaaacttgaat
gaggtaccagtaagaaatatgcagggagaacttctcagcctgtgagacggacaaaaactt
ccttgtcaggagttaactgaactaccaggcaggacagcagattttctgtctgaaaggcaa

>RAB27A 1500KB upstream region
cattttcttagaacattatccacgtgcataagaatgcggtcacattttaaagtgcctttt
gccgaacttaagtgctgccaattaataggccctgtgaacctcgtttctgcttatgtcctc
aatcaaaaggagcagacccccctcctttaggcactctccccacttctgccaagggcctctc
atttccatggcaccaaactcagtcacgctgcgctgtggaccagccttccctatggtgctc
cgtgttggcccaggttcaagatgctcttaaataacgcgctaaaaggatttttttctcat
gcactctgttccaccataatagctgaaacaagtctttattaaggagtcatatgatgctga
ttatatcttgatttgtcagttgtacagcaaaaccacagatcttccctgtttgtggcagga
aaaccacaaaccgtggtgcctgttggagttcttcctgaattcagttcctctttaaacact
acacatgcaaggagatgtggagaatttggcaggaaaagaagtggtccaaggccaatggaa
aaacatcaagtatatggaggaagtttaattaaacgtagtataaggaagaactgctttatt
aaacttgccatgcacacaagaaaattcctatgaatattaccacatgggggtctaaccga
atgccctttggaagggcggggggctgcaggtgtctgtctgcctccccacatgccctcagc
cctgcgaggcaacacccaagggtttggtgtctgcaggggcacggacctgtgcagaccagc
caggcgtgcagcagaggcagctcgcgtgcactccttgcagaactgcagttgcagtcagtg
tctgcgctctggagaacagctgtggaccaggatctcctggtttccgaacagagcagccct
atcactttaccagctgaaacagacaagagggtcttgctccaaggcagcgagttgcaggtt
ggtgcctgccttctggcatcctcggtctggatccagacaggagccttcgaggttcaggat
ccttttgaaaaggatgtgaattctttgcactttgggggcttcacacatactccactctg
atgacgtatgaaagaaaacacccaaggtgtgcaatcaaactgatttagcaagagaaatgg
ctgttttagctgcagggactgataaaccctgggcagcaccacttcctacagaagccggcc
ataccgtgacggttttcctcgctaatctttgcagcaacaaagcaagcaacagcagctcct
gattcatcctgcactgttgagtgaatgggcacgttttatccttgaagttgccaggctaca
gaagccttcagccggggacaaggctgcttccaaggactgccagagagttcccttcccctc
ttccacggtacctccctgcagcagtggcggcattcagcctcagggcctcccctggttcct
ctgcccagtcccccccccgcctcctccccaacacaggaaatcctgcttccaactgtgggg

>MLANA 1500KB upstream region
acttctcttttgctgaaagtaagtgacccaatttagattggttaagaggggcataatgga
gagatttaatccagtggattgttatgcccccaattcaaatcctctgtggtaattttaaaa
taatgaaaagggccatgaatttcctatagtataaccagttagatctttaatcaaatagtt
gttcatcaagtagtttaattacgttcaggggacttacccctaaatgtatatcagactgca
tcttgagttcctgaatttgagttgaagttaccagatttagatgcaaagctaaatgtattt
ttaatataagtatttattaaatttatatactgcttctttttgaagaggccctaagtggtg
tatataataagtgacggtaatatgacaccaatgcaaaataccatggaatacagaacaaaa
aaacaccatcggcatacaaagttcatttatagatatgaaaatgctgagaaacgtggaggt
tgagagggattgaatgttgtcatctcacccataaacagtcactgggaattcctaccagta
ccctcacctactactctcgcataagggcatgaattggttggaatctaattgctcaaagac
aaacagaaagtgataataccagaccttcaataaatcctatcacaacaaaacctgaaaaat
ctagaagatgaagaacacatttttagcatcttctccaaagtcctttcaacaaaaaaatgt
aaatgattgtttcaggagactgtaatgcctgattaattgtgctatttctttcatattaat
acttgaattgtttttaatgtgcttgttttctgtttcatttgaggaatatatcaacattgt
ttcgttatgaattattctgaatagagttttaaaagagcaggatgttgatagtaaattact
taatttcctcaccaaagcttttctcccagctattctttctcccagtattactccaaagga
aaaaggcaagtaggaagacgtattctgggggcaaaatgtaagaatcattagaagtttttt
tgcaccctaaatgtgagtgcatgcacagtttataccagattggacaggactggagatggg
tcataatcctggatcttattttgcttttagaaaactagcaaaataagctattgagttgta
taagtaggcactatctgatgacaggttttgaatacagagaaagttctattgcatcttata
atgtctaaaagataaaattgcctcagattgatcttaaatcctggtaatttatatagacac
atccttgcagttttcttgccataatacagaagtgatttttcattgttttctcccaggaat
tttttattcattttcccagtttaggtcatagctctggatttcccagatgttccccacaat
cctgatgaaatttctagatcagccaaggttagctaaatccccttttttgttagtattgcca
tggatttcaggaagactggtccttgtttttttataatatttattacatgtatagttttaaa

>DCT 1500KB upstream region
ttacatgtaatcaggaggacaatcaaaagcaagggaagggaaaatgaaaatctcaaacaa
ttaattaaaacaaactaacataactgaaaagaatacccacatatgtacactctaacagta
acaactatagttatagtacagaactttataaaccttctagagaattttgaaggtaaatcg
gaaaagaggagaacgttcttctgatcatttgcccaaggcaattggatcaaaagattaaga
aggcaagagagcaatcactgaaaataagtttggcccaaatatgtaaacaagcatgaggtt
ttagagaagctggtcctggttcccggtgcactaatgaattggaagacctcttaggaactt
ggaccacgactcatagatggttctgcttctagaaggcttgaatctttataattaccagtc
tgtgagccatctaacaggtggttgatcatctttgctttaaaaagtatttattttatagta
gtaggtatacagttaccatcttagatgctaggcaaacacagaattgtattaataaactga
tgagcctggtcagtaaaatttcttatttgctctctctaaacaacagaaaacgttcctccc
aaatatttaaaatttgctgaatgtggatgctcttagtcttccagtgataagtacaatctg
caagcctctaagtgaagatcactttctttgttgggacatgactgatgatgaaccgttcct
ctttacatcactgtatgagacacatcataaaaatggagattcattttaaacactgtaag
agtcttggtatcatagctagaaaacaggtggcagaaaatgtcatttgaatataacctgaa
tgtagatatcagctcactatgaaggctacaacatggtaatgtgtattgtcttctgccatg
tggtagctcgtgatcctttaaagttttcagagtgaattacaatattcaactatagttgct
gtggaggtccataattttgcaatctcttacaaagctctcaaagaaacttgcacccctcag
cccacagcatgaagtctgaggtcgtggtctcataaagcactgagcagaagaaaagataaa
gcaatgggaatcaaagacgttttgtctccctcgtccagggcttctcccccagcattccat
gggtttctttttgcttgtaatgtcatgtgctaatcataagggttttcaaaaagctaatcc
ttctaattagggtttactgacccccaaatataaaatctccagctacggcctcttggcaaa
caacaaagagctaaactgtttgcctattaaccacattcagtcctggtaatcttggattaa
gcaaagacccctgaaggttaacctagttgcagtgaggaggaaaaattgcttctcgggtca
gttcgaatggggcttgctcatccagctggagatggctaatgataatcttttcactgtatc
ctgcatttagaaccacaggctgagcagcagagttttggaagctctgggaattagatcttg

>AIM1 1500KB upstream region
caggattttgaagatacatgcataagactaaacatttttcaagaaagagagaagtgga
agcagactagaaaaaaatggaaataatagatgatagaaaagaaggcaaaaccacaggttt
gcctgacagacaaatcctaggacctacaaagactgtggcagcagcaaacatgcccactcc
ctttattggtttccttcttaatggaatattattttatgctttttttaaaatggtaggtt
ggcttgagtgtcaaaagaaaataaatgttaaatgtgtttttctgaaacctacatggaatt
tcagttcagattcaactaccactctgcttaagcattactcccaaatcacagcatatcagg
aaaatcctctgtcaactgcaaagacagaagatggccctgtgtattatttttcatcaaaca
tcaatataattttaaattttttgcatttttggagccaatggggaatttttttctatcataa
attgaaaaatggaggggtagcttctatcagggttataatctgggaaagaaggattacttt
taagaagtaatttaaaaaattgtaccatacttacaattcatgagtggaatagttacccca
agattcactatgcttcactccaatgtgcttttgctattcctttactcttttcctgtgtat
acatttttcagatatgcaaagtttagaattacgtttacagtacaagctgctacatgcctg
tatagtcagaaataaatcgctgagagttcagtggtgagaagagtattgccacatgaatgg
tgaccgggtgggcctgttttgcttctgagtccaaactattggccccatggactctgttgt
taaatacaaacatatcaggctttttgcaaaaataaaaatccctttaaaaaatctcctttg
cttcaaagaactcacagcagagaagacagtatgtcctggactaccttaaatgtagcaatt
tgtctttttcatatggtttcttatttatttatatatcaaatttatatagccgcccagttc
actctgggcagggtacaacgaagatataaaacaagagtttcctggatacttgcagccctt
ccttgcacaaactgttgatcatttcgtctccatcagaaagaaacaacaaccccttattact
gccttgcaatttatatcctcggatcagtcgagtctctcccttctcgggtaacacgcagta
tgaaggggagctggagtggcagtcaatttctgcttcaagaccacgaactggcagcccctt
taaacggaacaaaggagggtcccgagccccgagagaagtagttgcagtcgcggaaatagg

agcgagagataaaacttaataaaggcacgaagaggagacaggcgaattcgtggccgtcgc
ttgaaagtttgtacaagccaagctgaggaggtgggaagggtgcggtccgcaccgcggttg
gagcagctgacagggccggcggtgctcccgcagcctttaggaggcgctttggttctcccc

>MC1R 1500KB upstream region
tatccaaatataaattgcttctcagccttttggctaagatcaagggtgcacggtagacgt
aggccgtgactgatccacctggctcaccttgatggcaacggtcaggtgcagcgaccacct
tccttggtttccttctccatcataagtgatcactccggggttccaacaaaggtggcagtt
tggctgctttgccctgggtgcctccacatcagttagcatgatctagagattacggggcat
ttgtacatctggacaggcagaaaaattatcccttgatatgcccctacagatccaatctca
gaagaatttgactactaatcgaataacatgccataataataaagaaacagactcagaagt
agatgatgattctttcaaattcctcatggcagagacctttaccaccacccagttaaaaa
aaaaggggggggtcagaaaactggccgtgctgaaggctaaaagaaggcatcctatcaat
atatggggtgtgtgtagataactcaagaagtgattcagtggcaaatgctgaaccagctaa
tacaggtctcctgtagataggcccagtttgttacttaaggctactctgaacataacagga
accatgctgaaagaaatataggaaagggtacttaaaagaacggcatatcagatatggttg
acgctgtccttgcgacaacctggctcagcagcagcgtggcccagcactctctctgctgcc
acccgtggcccacattccaccactccaccttgggagcccaggtgatgataatccattcct
gtttctggttttgtagcacttcatcttcgggaaaggccatcccactgtcagtgtatttac
agaggcaaaacgcatcctgactttaaggactgtttcataacatctccctgcctttcaaag
tgctcgccgcctgacttatcatcatgtgcatcaccctctgccccccctgcaaaaggaggag
ctgcatctatccctttaaaaaagggaaaagaaagagttctttctttgctgtaaattttg
ctaatctattttggaggcagagacaaattcagcaattataacattaggaaggaagccaag
cagaactttgacatggacacaccacttcccaaggaagatgctgggggatcgctctcctta
gccgcataggctgaagaacaaaggagacctgcagaaaccaatgacaggggaagaaaggcc
aagaagccaccactttaggatccttggaacggtattgcgaccaaagaagtccttgtggaa
gcttgtgtctgcgctgctcgctttgctctcccggaccctttgttggggagcatgtgatct
caccatctgggattttctgggaggaaaagtgctgacagaggaacctgtagcctacaggtg
agtgtttgggacaggctaaaaatacctgcattcatctaaaatcacttcagaagaaacgct
cttccaaaatctgtgtccccccctcttatttctcactagcagcgccacaaagtgtttgag

>PMEL 1500KB upstream region
ttttgtggataatgatacaaacgtaataaagaagaaaaccctcatcaggatgagtagcct
tcttgcagaactatgccagcttttgttttcccccaaagttttgttataaggcgatacagg
aagtcttcatattgctgtggcaaagtggttgatctgtttttggtggtggttttcccaaat
ggcttgtagctgctcatacagatatccatctgtacttagaaaaatcaagacagctaaact
agtcttagattggaagtcaagcatatcagtttagttgaaaagcccccttttataccctgca
gtttcagaatttgtaaagaggtccatccctatacaacagtgtttctcaaccttggcaact
ttaagatgcgtggacttcaactcccagaattccccagccagcatggctggctgnnnnnnn
nnnnnnnnnnnnnnnnnnnnnnnnnnnnnnnnnnnnnnnnnnnnnnnnnnnnnnnnnnnn
nnnnnnnnnnnnnnnnnnnnnnnnnnnnnnnnnnnnnnnnnnnnnnnnnnnnnnnnnnnn
nnnnnnnnnnnnnnnnnnnnnnnnnnnnnnnnnnnnnnnnnnnnnnnnnnnnnnnnnnnn
nnnnnnnnnnnnnnnnnnnnnnnnnnnnnnnnnnnnnnnnnnnnnnnnnnnnnnnnnnnn
nnnnnnnnnnnnnnnnnnnnnncagccagccatgctggctggggattcttggagttgaag
gccacatcttaaagttgccaaggttgagaaacacagctatataatattttgtcttcacct
actgaattactctgtgtcaaattatttttctgtattggagtgggagtggttgtgtgctta
agtgactttcattctaacagatggctcacccatccctctgcattccatgactttgtccag
aaatgtaggaccacagcaaaccctgcctcgcctgctgtccaagaaaccctagaccatgcc
cttgcctgagaattatttttgatttaaattgtggattcctttgcctgctttgacttgctt
catatcactatctgcccaggcatccaatttcaaaggagattttgaagttacaagaatatg
aaatggttgcagatattcatgctcactttatatcagcctgtcctgtttctcctgttctca

gtgctagcacctctctttggaagaagtttttccagaatttgagatgccaccctcgttccc
cacttctagctctagaattgcccaggcatgcttctgtttcttctctctcatctctttctg
tagtgttccctggagccaccagtaccccagtttgagctctggtatattgctcatgtgcag
tgtccatcaacggggcagctggaaatctattcccttatgaggtggcctattggcgctgac
caggtcaggcaaattctggactaccattcagctagcaactggggaatcgccatttgactg
ctcttccacacataaatggctcacatggtgtagacaatgatcacactgagaagtagtcca

>OA1 1500KB upstream region
tgtagacttcaactctcnnnnnnnnnnnnnnnnnnnnnnnnnnnnnnnnnnnnnnnnnnn
nnnnnnnnnnnnnnnnnnnnnnnnnnnnnnnnnnnnnnnnnnnnnnnnnnnnnnnnnnnn
nnnnnnnngagtctagtgcagtgttttaaaacctaagcaatttaaaattgtgtagccttc
aactctcagaattcgggggttgaagtccacacatcttaaagttgccaagttttaaaaac
actggtcaggtgtaaaaaagggttactgaacctgggctgcaaaagtccagcagacaccac
tagacagctgcagcaatacagaaaactctatatcactgccatcatctagcagtcatctgg
atttctgagcccagatgtggtatggtagtccaaaggtaattcaagatgcactggatccat
gccttactttctcttccttctacttcatcatcttctcagagacgcaataacatctttgag
cagtaattagtgggcatgggggtccgatgatctcaccgctcaggcctctgaagtttttca
ggagtgccggcacagctacccgccggcgtgggaaaagctgttttagggcagtcaggagtt
caggaaacaaatctcagagcggtaacagaaccacgtgcagggaggaggatttatgcccca
gcaagtcatgctaagaaaacacataggcacaattgatgagaaacggtaatcctctgtccc
caatttacaaaacgcggaggttgaccctatgcagggagtccttttcagactgcgcctttt
cagaactcggaagagctgctccttttctctggcgcgccgcgccccaagagggctccgatc
cacccgctccagcgagaagccaattcggtttgagtgaagccctgccaacgcctcgaagca
aggggaatgaaagacacgcggttcgcccccccgctctccccccgcgtctcgctgccctag
tacgattttcccccggcggggaggaaaaaaaaaaaggannnnnnnnnnnnnnnnnnnnnn
nnnnnnnnnnnnnnnnnnnnnnnnnnnnnnnnnnnnnnnnnnnnnnnnnnnnnnnnnnnn
nnnnnnnnnnnnnnnnnnnnnnnnnnnnnnnnnnnnnnnnnnnnnnnnnnnnnnnnnnnn
nnnnnnnnnnnnnnnnnnnnnnnnnnnnnnnnnnnnnnnnnnnnnnnnnnnnnnnnnnnn
nnnnnnnnnnnnnnnnnnnnnnnnnnnnnnnnnnnnnnnnnnnnnnnnnnnnnnnnnnnn
nnnnnnnnnnnnnnnnnnnnnnnnnnnnnnnnnnnnnnnnnnnnnnnnnnnnnnnnnaaa
cggagccgggaacgccagtcgggggctttagccgccacttcgccacatgatccggccgcc
gcgtgacccccatctctaagccgaaggaggaattctgtacaaaggaaaggctgctcggag
cgggagaaagtgcgcggccctgcgtcccaccccgacctgccagtctcgcccaaactttcc

CREB
>Python-bivittatus gi|602656083|ref|XP_007433779.1| PREDICTED: cyclic AMP-responsive element-binding protein 1 isoform X1 [Python bivittatus]
MTMESGADNQQSGDAAVTEAENQQMTVPAQPQIATLAQVSMPAAHATSSAPTVTLVQLPNGQTVQVHGVI
QAAQPSVIQSPQVQTVQISTIAESEDSQESVDSVTDSQKRREILSRRPSYRKILNDLSSDAPGVPRIEEE
KSEEEATAPAITTVTVPTPIYQTSSGQYRTGVADIHHQYGNAVHAVAITQGGAIQLANNGTDGVQGLQTL
TMTNAAATQPGTTILQYAQTTDGQQILVPSNQVVVQAASGDVQTYQIRTAPTSTIAPGVVMASSPALPTQ
PAEEAARKREVRLMKNREAARECRRKKKEYVKCLENRVAVLENQNKTLIEELKALKDLYCHKSD

>Homo-sapiens gi|4758054|ref|NP_004370.1| cyclic AMP-responsive element-binding protein 1 isoform A [Homo sapiens]
MTMESGAENQQSGDAAVTEAENQQMTVQAQPQIATLAQVSMPAAHATSSAPTVTLVQLPNGQTVQVHGVI
QAAQPSVIQSPQVQTVQISTIAESEDSQESVDSVTDSQKRREILSRRPSYRKILNDLSSDAPGVPRIEEE
KSEEETSAPAITTVTVPTPIYQTSSGQYIAITQGGAIQLANNGTDGVQGLQTLTMTNAAATQPGTTILQY
AQTTDGQQILVPSNQVVVQAASGDVQTYQIRTAPTSTIAPGVVMASSPALPTQPAEEAARKREVRLMKNR
EAARECRRKKKEYVKCLENRVAVLENQNKTLIEELKALKDLYCHKSD

>Mus-musculus gi|82546874|ref|NP_598589.2| cyclic AMP-responsive element-binding protein 1 isoform A [Mus musculus]
MTMESGADNQQSGDAAVTEAENQQMTVQAQPQIATLAQVSMPAAHATSSAPTVTLVQLPNGQTVQVHGVI
QAAQPSVIQSPQVQTVQISTIAESEDSQESVDSVTDSQKRREILSRRPSYRKILNDLSSDAPGVPRIEEE
KSEEETSAPAITTVTVPTPIYQTSSGQYIAITQGGAIQLANNGTDGVQGLQTLTMTNAAATQPGTTILQY
AQTTDGQQILVPSNQVVVQAASGDVQTYQIRTAPTSTIAPGVVMASSPALPTQPAEEAARKREVRLMKNR
EAARECRRKKKEYVKCLENRVAVLENQNKTLIEELKALKDLYCHKSD

>Gallus-gallus gi|45383261|ref|NP_989781.1| cyclic AMP-responsive element-binding protein 1 [Gallus gallus]
MTMESGAENQQSGDAAVTEAETQQMTVQAQPQIATLAQVSMPAAHATSSAPTVTLVQLPNGQTVQVHGVI
QAAQPSVIQSPQVQTVQISTIAESEDSQESVDSVTDSQKRREILSRRPSYRKILNDLSSDAPGVPRIEEE
KSEEETAAPAIATVTVPTPIYQTSSGQYIAITQGGAIQLSNNGTDGVQGLQTLTMTNAAATQPGTTILQY
AQTTDGQQILVPSNQVVVQAASGDVQTYQIRTAPTSTIAPGVVMASSPALPTQPAEEAARKREVRLMKNR
EAARECRRKKKEYVKCLENRVAVLENQNKTLIEELKALKDLYCHKSD

>Ophiophagus-hannah_PARTIAL gi|565320082|gb|ETE71081.1| Cyclic AMP-responsive element-binding protein 1, partial [Ophiophagus hannah]
MESGAENQQSGDAAVTEAENQQMTVQAQPQIATLAQVSMPAAHATSSAPTVTLVQLPNGQTVQVHGVIQT
AQPSVIQSPQVQTVQISTIAESEDSQESVDSVTDSQKRREILSRRPSYRKILNDLSSDAPGVPRIEEEKS
EEEATAPAITTVTVPTPIYQTSSGQYIAITQGGAIQLANNGTDGVQGLQTLTMTNAAATQPGTTILQYAQ
TTDGQQILVPSNQVVLLQEMFRPTKFALHPQAPLHLVSSWHLPQLFPLNQQKKQHEKEKEAARECRRKKK
EYVKCLENRVAVLENQNKTLIEELKALKDLYCHKSD

>Anolis-carolinensis gi|637343695|ref|XP_008116978.1| PREDICTED: cyclic AMP-responsive element-binding protein 1 [Anolis carolinensis]
MTMESGADNQQSGDAAVTEAENQQMTVSMPAGHGTSSAPTVTLVQLPNGQTVQVHGVIQAAQPSVIQSPQ
VQTVQISTIAESEDSQESVDSVTDSQKRREILSRRPSYRKILNDLSSDAPGVPRIEEEKSEEEAAAPAIT
TVTVPTPIYQTSSGQYIAITQGGAIQLANNGTDGVQGLQTLTMTNAAATQPGTTILQYAQTTDGQQILVP
SNQVVVQAASGDVQTYQIRTAPTSTIAPGVVMASSPALPTQPAEEAARKREVRLMKNREAARECRRKKKE
YVKCLENRVAVLENQNKTLIEELKALKDLYCHKSD

>Alligator-sinensis gi|557293412|ref|XP_006029184.1| PREDICTED: cyclic AMP-responsive element-binding protein 1 [Alligator sinensis]
MTMESGAENQQSGDAAVTEAETQQMTVQAQPQIATLAQVSMPAAHATSSAPTVTLVQLPNGQTVQVHGVI
QAAQPSVIQSPQVQTVQISTIAESEDSQESVDSVTDSQKRREILSRRPSYRKILNDLSSDAPGVPRIEEE
KSEEETSAPAITTVTVPTPIYQTSSGQYIAITQGGAIQLANNGTDGVQGLQTLTMTNAAATQPGTTILQY
AQTTDGQQILVPSNQVVVQAASGDVQTYQIRTAPTSTIAPGVVMASSPALPTQPAEEAARKREVRLMKNR
EAARECRRKKKEYVKCLENRVAVLENQNKTLIEELKALKDLYCHKSD

>Canis-familiaris gi|928182529|ref|XP_013966467.1| PREDICTED: cyclic AMP-responsive element-binding protein 1 isoform X2 [Canis lupus familiaris]
MTMESGAENQQSGDAAVTEAENQQMTVQAQPQIATLAQVSMPAAHATSSAPTVTLVQLPNGQTVQVHGVI
QAAQPSVIQSPQVQTVQISTIAESEDSQESVDSVTDSQKRREILSRRPSYRKILNDLSSDAPGVPRIEEE
KSEEETSAPAITTVTVPTPIYQTSSGQYIAITQGGAIQLANNGTDGVQGLQTLTMTNAAATQPGTTILQY
AQTTDGQQILVPSNQVVVQAASGDVQTYQIRTAPTSTIAPGVVMASSPALPTQPAEEAARKREVRLMKNR
EAARECRRKKKEYVKCLENRVAVLENQNKTLIEELKALKDLYCHKSD

>Equus-caballus gi|149755007|ref|XP_001505170.1| PREDICTED: cyclic AMP-responsive element-binding protein 1 isoformX2 [Equus caballus]
MTMESGAENQQSGDAAVTEAENQQMTVQAQPQIATLAQVSMPAAHATSSAPTVTLVQLPNGQTVQVHGVI
QAAQPSVIQSPQVQTVQSSCKDLKRLFSGTQISTIAESEDSQESVDSVTDSQKRREILSRRPSYRKILND
LSSDAPGVPRIEEEKSEEETSAPAITTVTVPTPIYQTSSGQYIAITQGGAIQLANNGTDGVQGLQTLTMT
NAAATQPATTILQYAQTTDGQQILVPSNQVVVQAASGDVQTYQIRTAPTSTIAPGVVMASSPALPTQPAE
EAARKREVRLMKNREAARECRRKKKEYVKCLENRVAVLENQNKTLIEELKALKDLYCHKSD

LEF1
>Python-bivittatus gi|602628890|ref|XP_007421376.1| PREDICTED: lymphoid enhancer-binding factor 1 [Python bivittatus]
MPQLSGGGGDPELCATDEMIPFKDEGDPQKETIFAEISHPEEEGDLADIKSSLVNESETIPNSNGHGHEV
SRPGQSQEAYEKGREHPEEGKHTDGVLYNKGPSYSGYSGYIMMPNMNNDPYMSNGSLSPPIPRTSNKVPV
VQPSHAVHPLTPLITYSDEHFSPGTHPSHLPSDVNTKQGMTRHPPGPDLSTFYPLSPGSVGQITPPLGWQ
GQPVYSISGGFRQPFPSSLSVDTSMSRFSHHMIPGPPGPHTTGIPHPAIVTPQVKQEHPHTDSDLMHVKP
QHEQRKEQEPKRPHIKKPLNAFMLYMKEMRANVVAECTLKESAAINQILGRRWHALSREEQAKYYELARK
ERQLHMQLYPGWSARDNYGKKKKRKREKLQESTSGTAPRMTAAYI

>Mus-musculus gi|27735019|ref|NP_034833.2| lymphoid enhancer-binding factor 1 isoform a [Mus musculus]
MPQLSGGGGGGGDPELCATDEMIPFKDEGDPQKEKIFAEISHPEEEGDLADIKSSLVNESEIIPASNGHEV
VRQAPSSQEPYHDKAREHPDEGKHPDGGLYNKGPSYSSYSGYIMMPNMNSDPYMSNGSLSPPIPRTSNKV
PVVQPSHAVHPLTPLITYSDEHFSPGSHPSHIPSDVNSKQGMSRHPPAPEIPTFYPLSPGGVGQITPPIG
WQGQPVYPITGGFRQPYPSSLSGDTSMSRFSHHMIPGPPGPHTTGIPHPAIVTPQVKQEHPHTDSDLMHV
KPQHEQRKEQEPKRPHIKKPLNAFMLYMKEMRANVVAECTLKESAAINQILGRRWHALSREEQAKYYELA
RKERQLHMQLYPGWSARDNYGKKKKRKREKLQESTSGTGPRMTAAYI

>Ophiophagus-hannah_PARTIAL gi|565315274|gb|ETE67199.1| Lymphoid enhancer-binding factor 1, partial [Ophiophagus hannah]
MARCECHQYSVFDTRARFTVYAAQALDIRSSNKVPVVQPSHAVHPLTPLITYSDEHFSPGTHPSHLPSDV
STKQGMNRHPPGPDISTFYPLSPGSVGQITPPLGWQGQPVYPISGGFRHPYPSSLSVDTSMSRFSHHMIP
GPPGPHTTGIPHPAIVTPQVKQEHPHTDSDLMHVKPQHEQRKEQEPKRPHIKKPLNAFMLYMKEMRANWH
ALSREEQAKYYELARKERQLHMQLYPGWSARDNYSSSLILFFHFPELPISLHEKYLNAMKSANLGSGVAG

LYCAGNTTVTELYLGCRWYGFYYLPVFFHKIYYGGKKFLNFMSLPYENSLELEI

>Gallus-gallus gi|45384346|ref|NP_990344.1| lymphoid enhancer-binding factor 1 [Gallus gallus]
MPQLPGAGGGGGDPELCATDEMIPFKDEGDPQKEKIFAEISHPEEEGDLADIKSSLVNESEIAPGSGGHE
VSRQTPAQDSYHDKGREHPEEGKHPDGGLYSKGPSYSGYSGYIMMPNMNNDPYMPNGSLSPPIPRTSNKV
PVVQPSHAVHPLTPLITYSDEHFSPGSHPSHIPSDVSSKQGMSRHPPAPDIPTFYPLSPGGVGQITPPLG
WFSHHMIPGPPGPHTTGIPHPAIVTPQVKQEHPHNDSELMHVKPQHEQRKEQEPKRPHIKKPLNAFMLYM
KEMRANVVAECTLKESAAINQILGRRWHALSREEQAKYYELARKERQLHMQLYPGWSARDNYGKKKKRKR
EKLQESASGTGPRMTAAYI

>Homo-sapiens gi|29792286|gb|AAH50632.1| Lymphoid enhancer-binding factor 1 [Homo sapiens]
MPQLSGGGGGGGGDPELCATDEMIPFKDEGDPQKEKIFAEISHPEEEGDLADIKSSLVNESEIIPASNGH
EVARQAQTSQEPYHDKAREHPDDGKHPDGGLYNKGPSYSSYSGYIMMPNMNNDPYMSNGSLSPPIPRTSN
KVPVVQPSHAVHPLTPLITYSDEHFSPGSHPSHIPSDVNSKQGMSRHPPAPDIPTFYPLSPGGVGQITPP
LGWQGQPVYPITGGFRQPYPSSLSVDTSMSRFSHHMIPGPPGPHTTGIPHPAIVTPQVKQEHPHTDSDLM
HVKPQHEQRKEQEPKRPHIKKPLNAFMLYMKEMRANVVAECTLKESAAINQILGRRWHALSREEQAKYYE
LARKERQLHMQLYPGWSARDNYGKKKKRKREKLQESASGTGPRMTAAYI

> Alligator-sinensis gi|557297798|ref|XP_006031200.1| PREDICTED: lymphoid enhancer-binding factor 1 isoform X1 [Alligator sinensis]
MMPNMNNDPYMSNGSLSPPIPRTSNKVPVVQPSHAVHPLTPLITYSDEHFSPGSHPSHMPSDVNSKQGMS
RHPPAPDIPTFYPLSPGGVGQITPPLGWQGQPVYPISSGFRQPYPSSLSVDPSMSRFSHHMIPGPPGPHT
TGIPHPAIVTPQVKQEHPHNDSELMHVKPQHEQRKEQEPKRPHIKKPLNAFMLYMKEMRANVVAECTLKE
SAAINQILGRRWHALSREEQAKYYELARKERQLHMQLYPGWSARDNYGKKKKRKREKLQESASGGKRNTF
STCKTKAATPGPLLEMEAC

>Canis-familiaris gi|74002121|ref|XP_863402.1| PREDICTED: lymphoid enhancer-binding factor 1 isoform X3 [Canis lupus familiaris]
MPQLSGAGGGGGGGDPELCATDEMIPFKDEGDPQKEKIFAEISHPEEEGDLADIKSSLVNESEIIPTSNG
HEVARQAQTSQESYHDKAREHPDDGKHPDGGLYNKGPSYSSYSGYIMMPNMNNDPYMSNGSLSPPIPRTS
NKVPVVQPSHAVHPLTPLITYSDEHFSPGSHPSHLPSDVNSKQGMSRHPPAPEIPTFYPLSPGGVGQITP
PLGWFSHHMIPGPPGPHTTGIPHPAIVTPQVKQEHPHTDSDLMHVKPQHEQRKEQEPKRPHIKKPLNAFM
LYMKEMRANVVAECTLKESAAINQILGRRWHALSREEQAKYYELARKERQLHMQLYPGWSARDNYGKKKK
RKREKLQESTSGGKRNSFPTCKAKAATPGPLLEMEAC

>Thamnophis-sirtalis gi|927194465|ref|XP_013930427.1| PREDICTED: lymphoid enhancer-binding factor 1 isoform X1 [Thamnophis sirtalis]
MPQLSGGGGDPELCATDEMIPFKDEGDPQKETIFAEISHPEEEGDLADIKSSLVNESETIPSSNGHEVSR
QGQSQEVYEKSREHPEEGKHIDGVLYNKGPYSGYSGYIMMPNMNNDPYMSNGSLSPPIPRTSNKVPVVQP
SHAVHPLTPLITYSDEHFSPGTHPSHLPSDVNTKQGMNRHPPGPDISTFYPLSPGSVGQITPPLGWFSHH
MIPGPPGPHTTGIPHPAIVTPQVKQEHPHTDSDLMHVKPQHEQRKEQEPKRPHIKKPLNAFMLYMKEMRA
NVVAECTLKESAAINQILGRRWHALSREEQAKYYELARKERQLHMQLYPGWSARDNYGKKKKRKREKLQE
STSGIFMGTAPRMTAAYI

> Anolis-carolinensis gi|327283645|ref|XP_003226551.1| PREDICTED: lymphoid enhancer-binding factor 1 isoform X2 [Anolis carolinensis]
MPQLSGGGGGGGGGGGGGDPELCATDEMIPFKDEGDPQKEKIFAEISHPEEEGDLADIKSSLVNESETIS
SSNGHEVSRQGQPQEIYHDKGREHPEEGKHTDGGLYGKGPSYSGYSGYIMMPNMNNDPYMSNGSLSPPIP
RTSNKVPVVQPSHAVHPLTPLITYSDEHFSPGTHPSHLPSDVNSKQGMSRHPPGPDMPTFYPLSPGSVGQ

ITPPLGWQGQPVYPISSGFRQPYPSSLSVDTSMSRFSHHMIPGPPGPHTTGIPHPAIVTPQVKQEHPHTD
SDLMHVKPQHEQRKEQEPKRPHIKKPLNAFMLYMKEMRANVVAECTLKESAAINQILGRRWHALSREEQA
KYYELARKERQLHMQLYPGWSARDNYGKKKKRKREKLQESTSGAAPRMTAAYI

>Equus-caballus gi|545210283|ref|XP_005607957.1| PREDICTED: lymphoid enhancer-binding factor 1 isoform X3 [Equus caballus]
MVARQAQTSQEPYHDKAREHPDDGKHPDGGLYSKGPSYSSYSGYIMMPNMNSDPYMSNGSLSPPIPRTSN
KVPVVQPSHAVHPLTPLITYSDEHFSPGSHPSHIPSDVNSKQGMSRHPPAPEIPAFYPLSPGGVGQITPP
LGWQGQPVYPLTGGLRQPYPSSLSVDTPMSRFSHHMIPGPPGPHTTGIPHPAIVTPQVKQEHPHADSDLM
HVKPQHEQRKEQEPKRPHIKKPLNAFMLYMKEMRANVVAECTLKESAAINQILGRRWHALSREEQAKYYE
LARKERQLHMQLYPGWSARDNYGKKKKRKREKLQESTSGGKRSSFPTCKAKAATPGPLLEMEAC

FOXD3

>Python-bivittatus gi|602628796|ref|XP_007421330.1| PREDICTED: forkhead box protein D3 [Python bivittatus]
MTLSGSLSASDMSGQTVLTAEDVDIDVVGEGDEGAEEGGGGGPERGAPAAPPAAGWAAAAAPAAAAASSG
GKPKSSLVKPPYSYIALITMAILQSPQKKLTLSGICEFISNRFPYYREKFPAWQNSIRHNLSLNDCFVKI
PREPGNPGKGNYWTLDPQSEDMFDNGSFLRRRKRFKRHQQEQLREQTALMMHSFGAYSLAAGPAASSSAA
SAQTFLRPPVTAQSGLLAHQPLSLARSTAAIAPILSVPTNILSGQFLQPAASAATVQAKWPAQ

>Homo-sapiens gi|6912372|ref|NP_036315.1| forkhead box protein D3 [Homo sapiens]
MTLSGGGSASDMSGQTVLTAEDVDIDVVGEGDDGLEEKDSDAGCDSPAGPPELRLDEADEVPPAAPHHGQ
PQPPHQQPLTLPKEAAGAGAGPGGDVGAPEADGCKGGVGGEEGGASGGGPGAGSGSAGGLAPSKPKNSLV
KPPYSYIALITMAILQSPQKKLTLSGICEFISNRFPYYREKFPAWQNSIRHNLSLNDCFVKIPREPGNPG
KGNYWTLDPQSEDMFDNGSFLRRRKRFKRHQQEHLREQTALMMQSFGAYSLAAAAGAAGPYGRPYGLHPA
AAAGAYSHPAAAAAAAAAAALQYPYALPPVAPVLPPAVPLLPSGELGRKAAAFGSQLGPGLQLQLNSLGA
AAAAAGTAGAAGTTASLIKSEPSARPSFSIENIIGGGPAAPGGSAVGAGVAGGTGGSGGGSTAQSFLRPP
GTVQSAALMATHQPLSLSRTTATIAPILSVPLSGQFLQPAASAAAAAAAAAQAKWPAQ

>Gallus-gallus gi|45384420|ref|NP_990282.1| forkhead box protein D3 [Gallus gallus]
MTLSGGGSDMSGQTALAAEDVDIDVVGEGDDAPGKDGDGEARSPAALPLPLDEAAEPGEPERAARRAAAA
RQPGPGRPEGGRGGGGGGGGGEEGASGGGAAAAAAAGQSKPKSSLVKPPYSYIALITMAILQSPQKKLTL
SGICEFISNRFPYYREKFPAWQNSIRHNLSLNDCFVKIPREPGNPGKGNYWTLDPQSEDMFDNGSFLRRR
KRFKRHQQEHLRDQTALMMQGFGAYGLAGPYGRPYGLPPGAYPHPAALQYPYIPPVGPMLPPACPLLPSG
ELSRKAFNAQLGPSLQLQLSSLGAAGSIVKSEPSSRPSFSIENIIGGPAASSAPSAQTFLRPPVTVQSGL
VAHQPLALARTTAAIAPILSVPTNIIAGQFLQPPAAVQAKWPAQ

>Canis-familiaris gi|345800141|ref|XP_003434654.1| PREDICTED: forkhead box protein D3 [Canis lupus familiaris]
MTLSGGGSASDMSGQTVLTAEDVDIDVVGEGDDGLEEKDSDAGCDSPVGPPELRLDEADEVTPAAPLHGQ
PQPPHQQPLALPKEATGAGAGPGGEAGAPEADGCKGGVGGEEGGGSGGGGPGAGGGAAGGLAPSKPKNSL
VKPPYSYIALITMAILQSPQKKLTLSGICEFISNRFPYYREKFPAWQNSIRHNLSLNDCFVKIPREPGNP
GKGNYWTLDPQSEDMFDNGSFLRRRKRFKRHQQEHLREQTALMMQSFGAYSLAAAAGAAGPYGRPYGLHP
AAAAGAYSHPAAAAAAAAAAALQYPYAVPPVAPVLPPAVPLLPSGELGRKAAAFGSQLGPGLQLQLNSLG
AAAAAAGTAGAAGTTASLIKSEPSAWPSFSIENIIGGGPRGARGLRQRAAGGPGGRADGRDRGPGGGPRR
PLHSWSARPAPGPAPLPNPGLCLSSISSPPSPTPQHRPSAASTPSRTPRLAEQTRRPPARPPGIAFRTGF
RKAKE

>Thamnophis-sirtalis gi|927119627|ref|XP_013931405.1| PREDICTED: forkhead box protein D3 [Thamnophis sirtalis]
MTLSGNLSANDMSGQTVLTAEDVDIDVVGEEDDCLEDSGSSSSSLGGSPVEEKGSEEGSAPGAPSGAAAA
AGAAAAASSGGKPKSSLVKPPYSYIALITMAILQSPQKKLTLSGICEFISNRFPYYREKFPAWQNSIRHN
LSLNDCFVKIPREPGNPGKGNYWTLDPQSEDMFDNGSFLRRRKRFKRHQQEQLREQTALMMHSFGAYSLA
AAAAAAAASSSGPYGRAYGLHHGAYPHPAALQYPYIPPVGPMLPPAVPLLPSGELSRKAFNAQLSPSLQL
HLSGLGAVTGSGPAAGGVKAEPSGRPSFSIENIIGAGAGPAVSSSSAVSAQTFLRTPVTAQSGLLAHQPL
SLARTTAAIAPILSVPTNILSGQFLQPAASAGAVQAKWPAQ

>Anolis-carolinensis gi|637293528|ref|XP_008107538.1| PREDICTED: forkhead box protein D3 [Anolis carolinensis]
MTLSGSLSASDMSGQTVLTAEDVDIDVVGDEGDEAGTEEEARDGAGGLLEGPLPMPLPSQPPCLEEEAPE
AKQPAGEEGEKGAAGEDGAGSASSSSSSSSSSSSSAASKPKSSLVKPPYSYIALITMAILQSPQKKLTLS
GICEFISGRFPYYREKFPAWQNSIRHNLSLNDCFVKIPREPGNPGKGNYWTLDPQSEDMFDNGSFLRRRK
RFKRHQQEQLREQTALMMHSFGAYSLAAAAAAVAGGGGSPYGRAYGAYAHPAAVPYPYIPPVGPMLPPAV
PLLPASGAELSRKAFNAQLGLQLSGLGSGPIKAEPSASSRPSFSIENIIGGVGAAAAAAAAAAASSSSTPA
SSSSSISQTFLRPPVSVQSSLLAHQPLSLSRSTAAIGPLLSVPTNLLSGQFLPPSAAKWPAQ

>Chrysemys-picta-bellii gi|530581942|ref|XP_005284821.1| PREDICTED: forkhead box protein D3 [Chrysemys picta bellii]
MTLSGSSSTSDMSGQTVLTAEDVDIDVVGEGDEGLEKDSDGEASSPAGLHLEEAEELLLPKEAGAGGERS
GSGSPAEATPSGAADGGQGGRQRKPKNSLVKPPYSYIALITMAILQSPQKKLTLSGICEFISNRFPYYRE
KFPAWQNSIRHNLSLNDCFVKIPREPGNPGKGNYWTLDPQSEDMFDNGSFLRRRKRFKRHQQEHLREQTA
LMMQSFGAYSLASPYGRPYGLHPGAYPHPAAAALQYPYIPPVGPMLPPAVPLLPSSELSRKAFNSQLSPS
LQLQLNSLSAASSLIKSEPSSRPSFSIENIIGVSATSSGSSAQTFLRPPVTVQSALMAHQPLSLTRTTAA
IAPILSVPTNIISGQFLQPAASAAAVQAKWPAQ

POU3F2
>Python-bivittatus gi|602631326|ref|XP_007422568.1| PREDICTED: POU domain, class 3, transcription factor 2 [Python bivittatus]
MDGSILAKHAFDLRKASSALSETKRSIWESWVGGSPPPPPHHHHHEAHSDEDTPTSDDLEQFAKQFKQRR
IKLGFTQADVGLALGTLYGNVFSQTTICRFEALQLSFKNMCKLKPLLNKWLEEADSSSGSPTSIDKIAAQ
GRKRKKRTSIEVSVKGALESHFLKCPKPSAQEITSLADSLQLEKEVVRVWFCNRRQKEKRMTPPGGALPG
AEDVYGASRDTPPPHHGVQTPVQ

>Alligator-sinensis gi|557270480|ref|XP_006020515.1| PREDICTED: POU domain, class 3, transcription factor 2, partial [Alligator sinensis]
HAHHPHEPHSDEDTPTSDDLEQFAKQFKQRRIKLGFTQADVGLALGTLYGNVFSQTTICRFEALQLSFKN
MCKLKPLLNKWLEEADSSSGSPTSIDKIAAQGRKRKKRTSIEVSVKGALESHFLKCPKPSAQEITSLADS
LQLEKEVVRVWFCNRRQKEKRMTPPGGTLPGAEDVYGASRDTPPHHGVQTPVQ

>Canis-familiaris gi|928150842|ref|XP_013973738.1| PREDICTED: POU domain, class 3, transcription factor 2 [Canis lupus familiaris]
MQDQERSNGGGGDGSPWSTSPLGQPDIKPSVVVQQGGRGDELHGPGALQQQHQQQQQQQQQQQQQQQQQQ
QQQRPPHLVHHAANHHPGPGAWRSAAAAAHLPPSMGASNGGLLYSQPSFTVNGMLGAGGQPAGLHHHGLR
DAHDEPHHADHHPHPHSHPHQQPPPPPPPQGPPGHPGAHHDPHSDEDTPTSDDLEQFAKQFKQRRIKLGF
TQADVGLALGTLYGNVFSQTTICRFEALQLSFKNMCKLKPLLNKWLEEADSSSGSPTSIDKIAAQGRKRK
KRTSIEVSVKGALESHFLKCPKPSAQEITSLADSLQLEKEVVRVWFCNRRQKEKRMTPPGGTLPGAEDVY
GGSRDTPPHHGVQTPVQ

```
>Thamnophis-sirtalis gi|927123025|ref|XP_013907311.1| PREDICTED: POU
domain, class 3, transcription factor 2 [Thamnophis sirtalis]
MATTASNHYSLLTSSSPMVHAEPPGSMQPGAGYRDAQALVQADYALQSNGHPLSHAHQWITALSHGGGGG
GGGGGGGGGGGGGGNGGDSPWSPSPLGQQDIKPSVVQAGGRGDELQQQQQHHQQSQQQGRPPHLVHHAGS
HHAAVAAAVAWRTGGSAHLPPGMAAANGGGQGGLLYAQPPGFTVNGMLGSGQPGMHHHSLRDAHEEPPPP
PQQQQQQQQPPPHHPDHLAQQQQQQQQHPHHHPHGGPPPPPPPHHHHHEAHSDEDTPTSDDLEQFAKQFK
QRRIKLGFTQADVGLALGTLYGNVFSQTTICRFEALQLSFKNMCKLKPLLNKWLEEADSSSGSPTSIDKI
AAQGRKRKKRTSIEVSVKGALESHFLKCPKPSAQEITSLADSLQLEKEVVRVWFCNRRQKEKRMTPPGGA
LPGAEDVYGASRDTPPPHHGVQTPVQ


>Anolis-carolinensis gi|637253021|ref|XP_003215570.2| PREDICTED: POU
domain, class 3, transcription factor 2 [Anolis carolinensis]
MATTASNHYSLLAASSPMVHAEPPGSMQPGAGYRDAVQADYAALQSNGHPLSHAHQWIAALSHGGGGGGG
GGGGGGGGGGGGGGGGGSSGGDSPWSTSPDIKPSEPPPPPPPPPPPHHPDHLSQQQQQQQQQHHAPPPPH
HHHPHHPAHHPHHHEAHSDEDTPTSDDLEQFAKQFKQRRIKLGFTQADVGLALGTLYGNVFSQTTICRFE
ALQLSFKNMCKLKPLLNKWLEEADSSSGSPTSIDKIAAQGRKRKKRTSIEVSVKGALESHFLKCPKPSAQ
EITSLADSLQLEKEVVRVWFCNRRQKEKRMTPPGGTLPGAEDVYGASRDTPPPHHGEQDWGDALPLPSNH
PLTTLLQSCL



>Mus-musculus gi|6679423|ref|NP_032925.1| POU domain, class 3,
transcription factor 2 [Mus musculus]
MATAASNHYSLLTSSASIVHAEPPGGMQQGAGGYREAQSLVQGDYGALQSNGHPLSHAHQWITALSHGGG
GGGGGGGGGGGGGGGGGGDGSPWSTSPLGQPDIKPSVVVQQGGRGDELHGPGALQQQHQQQQQQQQQQQQ
QQQQQQQQQQQRPPHLVHHAANHHPGPGAWRSAAAAAHLPPSMGASNGGLLYSQPSFTVNGMLGAGGQPA
GLHHHGLRDAHDEPHHADHHPHPHSHPHQQPPPPPPPQGPPGHPGAHHDPHSDEDTPTSDDLEQFAKQFK
QRRIKLGFTQADVGLALGTLYGNVFSQTTICRFEALQLSFKNMCKLKPLLNKWLEEADSSSGSPTSIDKI
AAQGRKRKKRTSIEVSVKGALESHFLKCPKPSAQEITSLADSLQLEKEVVRVWFCNRRQKEKRMTPPGGT
LPGAEDVYGGSRDTPPHHGVQTPVQ


>Ophiophagus-hannah_PARTIAL gi|565323504|gb|ETE74074.1| POU domain, class
3, transcription factor 2, partial [Ophiophagus hannah]
FAKQFKQRRIKLGFTQADVGLALGTLYGNVFSQTTICRFEALQLSFKNMCKLKPLLNKWLEEADSSSGSP
TSIDKIAAQGRKRKKRTSIEVSVKGALESHFLKCPKPSAQEITSLADSLQLEKEVVRVWFCNRRQKEKRM
TPPGGALPGAEDVYGASRDTPPPHHGEEGAGGRGGRAQASSL


>Homo-sapiens gi|119568876|gb|EAW48491.1| POU domain, class 3,
transcription factor 2 [Homo sapiens]
MATAASNHYSLLTSSASIVHAEPPGGMQQGAGGYREAQSLVQGDYGALQSNGHPLSHAHQWITALSHGGG
GGGGGGGGGGGGGGGGGGDGSPWSTSPLGQPDIKPSVVVQQGGRGDELHGPGALQQQHQQQQQQQQQQQQ
QQQQQQQQQRPPHLVHHAANHHPGPGAWRSAAAAAHLPPSMGASNGGLLYSQPSFTVNGMLGAGGQPAGL
HHHGLRDAHDEPHHADHHPHPHSHPHQQPPPPPPPQGPPGHPGAHHDPHSDEDTPTSDDLEQFAKQFKQR
RIKLGFTQADVGLALGTLYGNVFSQTTICRFEALQLSFKNMCKLKPLLNKWLEEADSSSGSPTSIDKIAA
QGRKRKKRTSIEVSVKGALESHFLKCPKPSAQEITSLADSLQLEKEVVRVWFCNRRQKEKRMTPPGGTLP
GAEDVYGGSRDTPPHHGVQTPVQ


# MITF
```

>Python-bivittatus gi|602653814|ref|XP_007432759.1| PREDICTED: microphthalmia-associated transcription factor-like isoform X1 [Python bivittatus]
MTSRILLRQQLMREQMQEQERREQRQKQQAAQFMQQRGAVSQTPAINVSLPTSLPPATQVPMEVLKVQTH
LENPTKYHIQQAQRQQVKQYLSTTLANKHNNQALSLPCPNQPSDHVMPPGTGSSAPNSPMAMLTLNSNCE
KEGFYKFEEQSNRAENECRTLNTHSRVSCMQMDDVIDDIISLESSYNEEIFGLMDPALQIANTLPVSGNL
VDLYGNQGLPPPGLNISNSCPANLPNIKRELTGLGRLASQPSGEDFEGAGYTGVHEKGGISSFPVLLMEN
ILTDGGLTECSPLSSESEARALAKERQKKDNHNLIERRRRFNINDRIKELGTLIPKSNDPDMRWNKGTIL
KASVDYIRKLQREQQRTKELENRQKKLEHANRHLLLRIQELEMQARAHGLSIIPSTGLCSPEMVNRLIKQ
EPILDNCSQEILQQHHPELSSTTTLDLTDGTITFNGSLANVTESAPSTYAVPTKMGSKLEDMLMDDTLSP
AGVTDPLLSSVSPGASKTSSRRSSVSMEDTDHAC

>Canis-familiaris gi|50979188|ref|NP_001003337.1| microphthalmia-associated transcription factor [Canis lupus familiaris]
MLEMLEYNHYQVQTHLENPTKYHIQQAQRQQVKQYLSTTLANKHANQVLSLPCPNQPGDHVMPPVPGSSA
PNSPMAMLTLNSNCEKEGFYKFEEQNRAESECPTMNTHSRASCMQMDDVIDDIISLESSYNEEILGLMDP
ALQMANTLPVSGNLIDLYGNQGLPPPGLTISNSCPANLPNIKRELTACIFPTESEARALAKERQKKDNHN
LIERRRRFNINDRIKELGTLIPKSNDPDMRWNKGTILKASVDYIRKLQREQQRAKELENRQKKLEHANRH
LLLRIQELEMQARAHGLSLIPSTGLCSPDLVNRIIKQEPTLENCNQDLLQHHADLPCTTTLDLTDGSITF
NNNLGAGTESSQAYSVPTKMGSKLEDILMDDTLSPVGVTDPLLSSVSPGASKTSSRRSSMSMEETDHAC

>Equus-caballus gi|255522885|ref|NP_001157346.1| microphthalmia-associated transcription factor [Equus caballus]
MGHVENTSVVFPRAIFSTWEKETMELTLCLSLRSSSEEHPGASKPPISSSSMTSRILLRQQLMREQMQEQ
ERREQQQKLQAAQFMQQRVPVSQTPAINVSVPTTLPSATQVPMEVLKVQTHLENPTKYHIQQAQRQQVKQ
YLSTTLANKHANQVLSLPCPNQPGDHVMPPVPGSSAPNSPMAMLTLNSNCEKEGFYKFEEQNRAESECPS
MNTHSRASCMQMDDVIDDIISLESSYNEEILGLMDPALQMANTLPVSGNLIDLYSNQGLPPPGLTISNSC
PANLPNIKRELTESEARALAKERQKKDNHNLIERRRRFNINDRIKELGTLIPKSNDPDMRWNKGTILKAS
VDYIRKLQREQQRAKELENRQKKLEHANRHLLLRIQELEMQARAHGLSLIPSTGLCSPDLVNRIIKQEPA
LENCNQDLLQHHADLTCTTTLDLTDGTISFNNNLGTGTESNQAYSIPAKMGSKLEDILMDDTLSPVGVTD
PLLSSVSPGASKTSSRRSSMSMEETEHAC

>Ophiophagus-hannah_PARTIAL gi|565313510|gb|ETE65831.1| Microphthalmia-associated transcription factor, partial [Ophiophagus hannah]
MTSRILLRQQLMREQMQEQERREQRQKQQAAQFMQQRGAVSQTPAINVSLPASLPPATQVPMEVLKVQTH
LENPTKYHIQQAQRQQVKQYLSTTLANKHNNQALSLPCPNQPVDHVMPPGTGSSAPNSPMAMLTLNSNCE
KEGFYKFEEQNNRAENECQSLNTHPRVSCMQMDDVIDDIISLESSYNEEIFGLMDPALQIANTLPVSGNL
VDLYGNQGLPPPGLNISNSCPANLPNIKRELTGYTLYLPPTKVEAGIFARYVSQSIYEVECSREISKFTL
QLLPFNIILNLITSLTCISYVFPIESEARALAKERQKKDNHNLIERRRRFNINDRIKELGTLIPKSNDPD
MRWNKGTILKASVDYIRKLQREQQRTKELENRQKKLEHANRHLLLRIQVC

>Gallus-gallus gi|46048817|ref|NP_990360.1| microphthalmia-associated transcription factor [Gallus gallus]
MTSRILLRQQLMREQMQEQERREQQQKQQAAQFMQQRVPVSQTPAINVSVPASLPPATQVPMEVLKVQTH
LENPTKYHIQQAQRQQVKQYLSTTLANKHANQALSLPCPNQPGDHVMPPGTGSSAPNSPMAMLTLNSNCE
KEGFYKFEEQSRVESECPALNTHSRASCMQMDDVIDDIISLESSYNEEILGLMDPALQMANTLPVSGNLI
DLYGNQSMPPPGLNISNSCPANLPNIKRELTESEARALAKERQKKDNHNLIERRRRFNINDRIKELGTLI
PKSNDPDMRWNKGTILKASVDYIRKLQREQQRTKELENRQKKLEHANRHLLLRIQELEMQARAHGLSLVP
STGICSPDMVNRVIKQEPVLDNCNQDLMPHHTDLSCTTTLDLTDGTITFSDNLGNVTEPTGTYSVPAKMG
SKLEDILMDDTLSPVGVTDPLLSSVSPGASKTSSRRSSVSMEDTDHAC

>Mus-musculus gi|148666943|gb|EDK99359.1| microphthalmia-associated transcription factor [Mus musculus]
MLEMLEYSHYQVQTHLENPTKYHIQQAQRHQVKQYLSTTLANKHASQVLSSPCPNQPGDHAMPPVPGSSA
PNSPMAMLTLNSNCEKEAFYKFEEQSRAESECPGMNTHSRASCMQMDDVIDDIISLESSYNEEILGLMDP
ALQMANTLPVSGNLIDLYSNQGLPPPGLTISNSCPANLPNIKRELTACIFPTESEARALAKERQKKDNHN
LIERRRRFNINDRIKELGTLIPKSNDPDMRWNKGTILKASVDYIRKLQREQQRAKDLENRQKKLEHANRH
LLLRVQELEMQARAHGLSLIPSTGLCSPDLVNRIIKQEPVLENCSQELVQHQADLTCTTTLDLTDGTITF
TNNLGTMPESSPAYSIPRKMGSNLEDILMDDALSPVGVTDPLLSSVSPGASKTSSRRSSMSAEETEHAC

>Gallus-gallus gi|725825443|gb|AIY34493.1| microphthalmia-associated transcription factor [Gallus gallus]
MTSRILLRQQLMREQMQEQERREQQQKQQAAQFMQQRVPVSQTPAINVSVPASLPPATQVPMEVLKVQTH
LENPTKYHIQQAQRQQVKQYLSTTLANKHANQALSLPCPNQPGDHVMPPGTGSSAPNSPMAMLTLNSNCE
KEGFYKFEEQSRVESECPALNTHSRASCMQMDDVIDDIISLESSYNEEILGLMDPALQMANTLPVSGNLI
DLYGNQSMPPPGLNISNSCPANLPNIKRELTESEARALAKERQKKDNHNLIERRRRFNINDRIKELGTLI
PKSNDPDMRWNKGTILKASVDYIRKLQREQQRTKELENRQKKLEHANRHLLLRIQELEMQARAHGLSLVP
STGICSPDMVNRVIKQEPVLDNCNQDLMPHHTDLSCTTTLDLTDGTITFSDNLGNVTEPTGTYSVPAKMG
SKLEDILMDDTLSPVGVTDPLLSSVSPGASKTSSRRSSVSMEDTDHAC

>Alligator-mississippiensis gi|564263951|ref|XP_006270831.1| PREDICTED: microphthalmia-associated transcription factor isoform X1 [Alligator mississippiensis]
MTSRILLRQQLMREQMQEQERREQQQKQQAAQFMQQRVPVSQTPAINVSVPSSLPPATQVPMEVLKVQTH
LENPTKYHIQQAQRQQVKQYLSTTLANKHANQALSLPCPNQPGDHVMPSGTGSSAPNSPMAMLTLNSNCE
KEGLYKFEEQSRLESECPALNTHSRTSCMQMDDVIDDIISLESSYSEEILGLMDPALQMANTLPVSGNLI
DLYGNHTSMPPGLNISNSCPANLPNIKRELTACIFPTESEARALAKERQKKDNHNLIERRRRFNINDRIK
ELGTLIPKSNDPDMRWNKGTILKASVDYIRKLQREQQRTKELENRQKKLEHANRHLLLRIQELEMQARAH
GLSLVPSTGLCSPDVNRVIKQESVIDNCNPDIVQHRTDLSCTTTLDLTDGTITFSDNLGNVTDSSSAYSV
PTKMGSKLEDILMDDTLSPVGVTDPLLSSVSPGASKTSSRRSSMSMEDTDHTC

>Chrysemys-picta-bellii gi|641792697|ref|XP_008177703.1| PREDICTED: microphthalmia-associated transcription factor isoform X1 [Chrysemys picta bellii]
MLNNLPRTLWEQVSMQGKEVEKFSAFSPKIGLLEHASFWAALSCGARNKTESSSSYGSSSLSVKHRPRWG
PEPSLGFQGAIAVQIHNDDSGKTLTYQVQLQILSSSEHPGASKPPLSSSSMTSRILLRQQLMREQMQEQE
RREQQQKQQAAQFMQQRVPLSQTPAINVSVPSNLPPPTQVPMEVLKVQTHLENPTKYHIQQAQRQQVKQY
LSTTLANKHTNQALSLPCPNQPGDHVMPPGTGSSAPNSPMAMLTLNSNCEKEGFYKFEEQSRVESECPAL
NTHTRASCMQMDDVIDDIISLESSYNEEILGLMDPALQMANTLPVSGNLIDLYGNQGMPPSGLNVSNSCP
ANLPNIKRELTACIFPTESEARALAKERQKKDNHNLIERRRRFNINDRIKELGTLIPKSNDPDMRWNKGT
ILKASVDYIRKLQREQQRTKELENRQKKLEHANRHLLLRIQELEMQARAHGLSLVPSTGLCSPDVVNRVI
KQEPVIDNCNQDVLQHRSDLSCTTTLDLTDGTITFNDNLGTYSVPTKAGSKLEDILMDDTLSPVGVTDPL
LSSVSPGASKTSSRRSSMSMEDTDHAC

LEF-1
>Python-bivittatus gi|602628890|ref|XP_007421376.1| PREDICTED: lymphoid enhancer-binding factor 1 [Python bivittatus]
MPQLSGGGGDPELCATDEMIPFKDEGDPQKETIFAEISHPEEEGDLADIKSSLVNESETIPNSNGHGHEV
SRPGQSQEAYEKGREHPEEGKHTDGVLYNKGPSYSGYSGYIMMPNMNNDPYMSNGSLSPPIPRTSNKVPV

VQPSHAVHPLTPLITYSDEHFSPGTHPSHLPSDVNTKQGMTRHPPGPDLSTFYPLSPGSVGQITPPLGWQ
GQPVYSISGGFRQPFPSSLSVDTSMSRFSHHMIPGPPGPHTTGIPHPAIVTPQVKQEHPHTDSDLMHVKP
QHEQRKEQEPKRPHIKKPLNAFMLYMKEMRANVVAECTLKESAAINQILGRRWHALSREEQAKYYELARK
ERQLHMQLYPGWSARDNYGKKKKRKREKLQESTSGTAPRMTAAYI

>Mus-musculus gi|27735019|ref|NP_034833.2| lymphoid enhancer-binding
factor 1 isoform a [Mus musculus]
MPQLSGGGGGGDPELCATDEMIPFKDEGDPQKEKIFAEISHPEEEGDLADIKSSLVNESEIIPASNGHEV
VRQAPSSQEPYHDKAREHPDEGKHPDGGLYNKGPSYSSYSGYIMMPNMNSDPYMSNGSLSPPIPRTSNKV
PVVQPSHAVHPLTPLITYSDEHFSPGSHPSHIPSDVNSKQGMSRHPPAPEIPTFYPLSPGGVGQITPPIG
WQGQPVYPITGGFRQPYPSSLSGDTSMSRFSHHMIPGPPGPHTTGIPHPAIVTPQVKQEHPHTDSDLMHV
KPQHEQRKEQEPKRPHIKKPLNAFMLYMKEMRANVVAECTLKESAAINQILGRRWHALSREEQAKYYELA
RKERQLHMQLYPGWSARDNYGKKKKRKREKLQESTSGTGPRMTAAYI

>Ophiophagus-hannah_PARTIAL gi|565315274|gb|ETE67199.1| Lymphoid
enhancer-binding factor 1, partial [Ophiophagus hannah]
MARCECHQYSVFDTRARFTVYAAQALDIRSSNKVPVVQPSHAVHPLTPLITYSDEHFSPGTHPSHLPSDV
STKQGMNRHPPGPDISTFYPLSPGSVGQITPPLGWQGQPVYPISGGFRHPYPSSLSVDTSMSRFSHHMIP
GPPGPHTTGIPHPAIVTPQVKQEHPHTDSDLMHVKPQHEQRKEQEPKRPHIKKPLNAFMLYMKEMRANWH
ALSREEQAKYYELARKERQLHMQLYPGWSARDNYSSSLILFFHFPELPISLHEKYLNAMKSANLGSGVAG
LYCAGNTTVTELYLGCRWYGFYYLPVFFHKIYYGGKKFLNFMSLPYENSLELEI

>Gallus-gallus gi|45384346|ref|NP_990344.1| lymphoid enhancer-binding
factor 1 [Gallus gallus]
MPQLPGAGGGGGDPELCATDEMIPFKDEGDPQKEKIFAEISHPEEEGDLADIKSSLVNESEIAPGSGGHE
VSRQTPAQDSYHDKGREHPEEGKHPDGGLYSKGPSYSGYSGYIMMPNMNNDPYMPNGSLSPPIPRTSNKV
PVVQPSHAVHPLTPLITYSDEHFSPGSHPSHIPSDVSSKQGMSRHPPAPDIPTFYPLSPGGVGQITPPLG
WFSHHMIPGPPGPHTTGIPHPAIVTPQVKQEHPHNDSELMHVKPQHEQRKEQEPKRPHIKKPLNAFMLYM
KEMRANVVAECTLKESAAINQILGRRWHALSREEQAKYYELARKERQLHMQLYPGWSARDNYGKKKKRKR
EKLQESASGTGPRMTAAYI

>Homo-sapiens gi|29792286|gb|AAH50632.1| Lymphoid enhancer-binding factor
1 [Homo sapiens]
MPQLSGGGGGGGGDPELCATDEMIPFKDEGDPQKEKIFAEISHPEEEGDLADIKSSLVNESEIIPASNGH
EVARQAQTSQEPYHDKAREHPDDGKHPDGGLYNKGPSYSSYSGYIMMPNMNNDPYMSNGSLSPPIPRTSN
KVPVVQPSHAVHPLTPLITYSDEHFSPGSHPSHIPSDVNSKQGMSRHPPAPDIPTFYPLSPGGVGQITPP
LGWQGQPVYPITGGFRQPYPSSLSVDTSMSRFSHHMIPGPPGPHTTGIPHPAIVTPQVKQEHPHTDSDLM
HVKPQHEQRKEQEPKRPHIKKPLNAFMLYMKEMRANVVAECTLKESAAINQILGRRWHALSREEQAKYYE
LARKERQLHMQLYPGWSARDNYGKKKKRKREKLQESASGTGPRMTAAYI

> Alligator-sinensis gi|557297798|ref|XP_006031200.1| PREDICTED: lymphoid
enhancer-binding factor 1 isoform X1 [Alligator sinensis]
MMPNMNNDPYMSNGSLSPPIPRTSNKVPVVQPSHAVHPLTPLITYSDEHFSPGSHPSHMPSDVNSKQGMS
RHPPAPDIPTFYPLSPGGVGQITPPLGWQGQPVYPISSGFRQPYPSSLSVDPSMSRFSHHMIPGPPGPHT
TGIPHPAIVTPQVKQEHPHNDSELMHVKPQHEQRKEQEPKRPHIKKPLNAFMLYMKEMRANVVAECTLKE
SAAINQILGRRWHALSREEQAKYYELARKERQLHMQLYPGWSARDNYGKKKKRKREKLQESASGGKRNTF
STCKTKAATPGPLLEMEAC

>Canis-familiaris gi|74002121|ref|XP_863402.1| PREDICTED: lymphoid
enhancer-binding factor 1 isoform X3 [Canis lupus familiaris]
MPQLSGAGGGGGGDPELCATDEMIPFKDEGDPQKEKIFAEISHPEEEGDLADIKSSLVNESEIIPTSNG
HEVARQAQTSQESYHDKAREHPDDGKHPDGGLYNKGPSYSSYSGYIMMPNMNNDPYMSNGSLSPPIPRTS

NKVPVVQPSHAVHPLTPLITYSDEHFSPGSHPSHLPSDVNSKQGMSRHPPAPEIPTFYPLSPGGVGQITP
PLGWFSHHMIPGPPGPHTTGIPHPAIVTPQVKQEHPHTDSDLMHVKPQHEQRKEQEPKRPHIKKPLNAFM
LYMKEMRANVVAECTLKESAAINQILGRRWHALSREEQAKYYELARKERQLHMQLYPGWSARDNYGKKKK
RKREKLQESTSGGKRNSFPTCKAKAATPGPLLEMEAC

>Thamnophis-sirtalis gi|927194465|ref|XP_013930427.1| PREDICTED: lymphoid enhancer-binding factor 1 isoform X1 [Thamnophis sirtalis]
MPQLSGGGGDPELCATDEMIPFKDEGDPQKETIFAEISHPEEEGDLADIKSSLVNESETIPSSNGHEVSR
QGQSQEVYEKSREHPEEGKHIDGVLYNKGPYSGYSGYIMMPNMNNDPYMSNGSLSPPIPRTSNKVPVVQP
SHAVHPLTPLITYSDEHFSPGTHPSHLPSDVNTKQGMNRHPPGPDISTFYPLSPGSVGQITPPLGWFSHH
MIPGPPGPHTTGIPHPAIVTPQVKQEHPHTDSDLMHVKPQHEQRKEQEPKRPHIKKPLNAFMLYMKEMRA
NVVAECTLKESAAINQILGRRWHALSREEQAKYYELARKERQLHMQLYPGWSARDNYGKKKKRKREKLQE
STSGIFMGTAPRMTAAYI

> Anolis-carolinensis gi|327283645|ref|XP_003226551.1| PREDICTED: lymphoid enhancer-binding factor 1 isoform X2 [Anolis carolinensis]
MPQLSGGGGGGGGGGGGGDPELCATDEMIPFKDEGDPQKEKIFAEISHPEEEGDLADIKSSLVNESETIS
SSNGHEVSRQGQPQEIYHDKGREHPEEGKHTDGGLYGKGPSYSGYSGYIMMPNMNNDPYMSNGSLSPPIP
RTSNKVPVVQPSHAVHPLTPLITYSDEHFSPGTHPSHLPSDVNSKQGMSRHPPGPDMPTFYPLSPGSVGQ
ITPPLGWQGQPVYPISSGFRQPYPSSLSVDTSMSRFSHHMIPGPPGPHTTGIPHPAIVTPQVKQEHPHTD
SDLMHVKPQHEQRKEQEPKRPHIKKPLNAFMLYMKEMRANVVAECTLKESAAINQILGRRWHALSREEQA
KYYELARKERQLHMQLYPGWSARDNYGKKKKRKREKLQESTSGAAPRMTAAYI

>Equus-caballus gi|545210283|ref|XP_005607957.1| PREDICTED: lymphoid enhancer-binding factor 1 isoform X3 [Equus caballus]
MVARQAQTSQEPYHDKAREHPDDGKHPDGGLYSKGPSYSSYSGYIMMPNMNSDPYMSNGSLSPPIPRTSN
KVPVVQPSHAVHPLTPLITYSDEHFSPGSHPSHIPSDVNSKQGMSRHPPAPEIPAFYPLSPGGVGQITPP
LGWQGQPVYPLTGGLRQPYPSSLSVDTPMSRFSHHMIPGPPGPHTTGIPHPAIVTPQVKQEHPHADSDLM
HVKPQHEQRKEQEPKRPHIKKPLNAFMLYMKEMRANVVAECTLKESAAINQILGRRWHALSREEQAKYYE
LARKERQLHMQLYPGWSARDNYGKKKKRKREKLQESTSGGKRSSFPTCKAKAATPGPLLEMEAC
